# Global, regional, and national trends for childhood myocarditis from 1990 to 2021: health inequality and age-period-cohort analysis for the Global Burden of Disease Study 2021

**DOI:** 10.3389/fped.2025.1566010

**Published:** 2025-12-05

**Authors:** Hui-Juan Fang, Bao-Peng Liu, Bo Han, Jie Tian

**Affiliations:** 1 Department of Cardiology, National Clinical Research Center for Child Health and Disorders, Ministry of Education Key Laboratory of Child Development and Disorders, China International Science and Technology Cooperation Base of Child Development and Critical Disorders, Chongqing Key Laboratory of Pediatric Metabolism and Inflammatory Diseases, Key Laboratory of Children’s Important Organ Development and Diseases of Chongqing Municipal Health Commission, National Clinical Key Cardiovascular Specialty, Children’s Hospital of Chongqing Medical University, Chongqing, China; 2 Department of Pediatrics, Shandong Provincial Clinical Research Center for Children’s Health and Disease Office, Shandong Provincial Hospital Affiliated to Shandong First Medical University, Jinan, China; 3 Department of Epidemiology, School of Public Health, Cheeloo College of Medicine, Shandong University, Jinan, Shandong, China

**Keywords:** childhood myocarditis, health inequality, age–period–cohort analysis, global burden, prevalence

## Abstract

**Background:**

Childhood myocarditis (CM) may have a profound impact on lifelong health worldwide. This study aimed to assess the trends and explore the health inequality along with age–period–cohort (APC) analysis for CM from 1990 to 2021.

**Methods:**

This study is based on the database for Global Burden of Disease (GBD) 2021. The age-standardized rate (ASR) of incidence, prevalence, mortality, and disability-adjusted life years (DALYs) was used to assess CM burden. Estimated annual percentage changes (EAPC) in ASR from 1990 to 2021 were used to calculate the time trends. Health inequalities related to the sociodemographic index (SDI) and APC effect for CM burden were studied in this study.

**Findings:**

A significant decreasing trend for ASR of incidence, prevalence, mortality, and DALYs from 1990 to 2021 was found for CM globally. The increased EAPC of prevalence for CM was still high in high SDI regions. Higher ASR of prevalence and incidence and lower ASR of mortality and DALYs were significantly associated with higher SDI levels. A significant increase in SDI-related health inequalities was found from 1990 to 2021 for ASR prevalence. Higher risk for CM prevalence was found in the younger ages, recent periods, and birth cohort among high SDI regions. Non-optimal temperature was also significantly associated with elevated risk of mortality and DALYs for CM.

**Interpretation:**

Although CM burden has been decreasing in recent years, the disproportionate CM burden globally warrants caution. Effective methods should be used to decrease CM burden in the future.

## Introduction

Myocarditis, an inflammatory condition of the myocardium, arises from diverse infectious and non-infectious etiologies, including viral infections, autoimmune disorders, hypersensitivity reactions, pharmacologic agents, and environmental toxins (1). This condition represents a clinically significant contributor to global disease burden, disproportionately affecting pediatric and young adult populations. Clinical manifestations in children range from subclinical presentations to severe complications such as heart failure, life-threatening arrhythmias, cardiogenic shock, and sudden cardiac death (2, 3). Early diagnosis of myocarditis remains challenging due to non-specific symptoms and the absence of definitive biomarkers. Furthermore, an incomplete understanding of its pathogenic mechanisms has hindered the development of targeted therapies; approximately 9%–30% of pediatric cases progress to dilated cardiomyopathy.

Myocarditis carries a high likelihood of progression to dilated cardiomyopathy and heart failure in the long term, rendering pediatric myocarditis a persistent clinical challenge in both diagnosis and management (2, 4). Elucidating its epidemiological characteristics and disease burden could inform improved clinical strategies. To date, limited global evidence on the age-standardized childhood myocarditis (CM) burden, including incidence, prevalence, and mortality, remains limited. A Global Burden of Disease (GBD) study analyzing trends from 1990 to 2019 reported declining age-standardized incidence and mortality rates for myocarditis and cardiomyopathy among children and adolescents (<19 years), with males exhibiting lower rates than females (5). However, other analyses focused solely on myocarditis have not quantified burden indices in pediatric populations (6–11). Further research leveraging updated GBD datasets is warranted to assess the evolving global, regional, and national CM burden.

Upon previous evidence on epidemiological characteristics for CM, this study seeks to delineate the global, regional, and national time trends for CM burden, including incidence, prevalence, disability-adjusted life years (DALYs), and mortality in total and by sex, investigate the health inequality related to CM burden, and examine the age–period–cohort (APC) effect on CM burden taking advantage of update database, GBD 2021.

## Methods

### Data source

The data used in the present study were derived from GBD 2021 (https://vizhub.healthdata.org/gbd-results/), including an epidemiological index of 371 diseases and injuries along with 88 risk factors (12–14). GBD 2021 was newly updated and included 21 GBD regions and 204 countries/territories from 1990 to 2021. More details of the GBD 2021 database resources and validation can be found on the Global Health Data Exchange (GHDx) web tool (http://ghdx.healthdata.org/). In this study, we extracted sex- and location-specific estimates of incidence, prevalence, DALYs, and mortality with the corresponding 95% uncertainty intervals (UIs) for CM from 1990 to 2021. We also divided the estimates according to different classifications, namely, 21 GBD regions and 204 countries/territories.

GBD 2021 complies with Guidelines for Accurate and Transparent Health Estimates Reporting (GATHER). More than 10,000 collaborators from 150 countries or territories provided, reviewed, or analyzed the data to generate the index of GBD 2021 (13). No ethical approval was required for the present study because no individual information was included.

### Disease definitions

After a systematic review of myocarditis in past cycles, GBD 2021 only uses hospital admission incidence data to estimate myocarditis incidence and prevalence (13). Vital registration data were used to model deaths due to myocarditis in the GBD 2021 (14). Myocarditis was diagnosed using International Classification of Diseases (ICD) codes. Death by myocarditis was coded by B33.2, I40–I41.9, and I51.4 for ICD-10 and 422–422.9 for ICD-9. Non-fatal causes of myocarditis were widely coded by B33.2–B33.20, B33.22–B33.24, D86.85, I40–I41.8, and I51.4–I51.6 for ICD-10 and 074.2, 074.23, 422–422.99, and 429.0–429.1 for ICD-9 (15). More details can also be found in the GHDx (https://ghdx.healthdata.org/record/ihme-data/gbd-2021-cause-icd-code-mappings).

### Estimation framework

The burden indices including incidence, prevalence, DALYs, and mortality were used in the present study and were calculated by employing complicated modeling techniques. The details for the estimation framework can be found in previous studies (12, 14). Shortly, incidence and prevalence were estimated by Disease Modeling Meta-Regression, version 2.1 (DisMod-MR 2.1), using a Bayesian disease modeling meta-regression tool, considering the effect of missing raw epidemiological data and geospatial data. Cause of death estimates for most diseases and injuries were modeled via the Cause of Death Ensemble model (CoDEm), which incorporated vital registration and verbal autopsy data, including those with non-specific codes. DALYs were calculated by the sum of years of life lost (YLL) and years lived with disability (YLD) after correction for comorbidity. The workflow diagram for the global CM burden is shown in Supplementary Figure S1.

### Sociodemographic index and risk factors for CM

The sociodemographic index (SDI), which was a composite metric that represented development levels, was calculated by the geometric mean of three factors: the lag-distributed income per capita, average years of schooling, and the fertility rate in females younger than 25 years for a given location (13). SDI scores ranged from 0 to 1, and a higher score indicated greater development. GBD 2021 provided the data for SDI (16) and the corresponding classifications (low, low–medium, medium, medium–high, and high) for countries/territories and regions, which were used in the present study.

Risk factors for CM burden were also reported in this study. In the present database, there were four levels and an overarching aggregate of all risk factors combined for mortality and DALYs. More details about the methodological description can be found in previous studies (12).

### Statistical analysis

#### Description of the time trends for CM burden

The global, regional, and national burden indices were described by age-standardized rate (ASR) for incidence, prevalence, DALYs, and mortality per 100,000 populations, respectively, by calculating GBD 2021 data released on the GHDx. Estimated annual percentage changes (EAPC) with corresponding 95% CI in ASR from 1990 to 2021 were used to calculate the time trends for CM (17). The calculation involved using a log-linear regression model to explore the natural logarithm of the ASR trend by calendar year. More details can be found in previous studies (17, 18). For the time trend of CM burden, a negative EAPC means decreasing, and a positive EAPC means increasing.

#### Cross-country inequality analysis

Relevant analyses were performed to explore the associations of regional and national levels of SDI with ASR. Two standard metrics, namely, the slope index of inequality and the concentration index which could reflect absolute and relative gradient inequality, were applied to measure the inequality of ASR across countries/territories (19, 20). The slope index of inequality was measured by regressing national ASR on SDI, and the concentration index was measured by numerically integrating the areas under the Lorenz concentration curve (21).

#### The analysis for the APC effect for CM burden

The APC (APC) model was used to explore the trend of ASR by age, period, and birth cohort to solve the completely linear association of age, period, and cohort (cohort = period − age), to obtain an independent effect (18, 22, 23). Details of usage and methods are reported in previous studies (24, 25). Age effects gave the estimation of ASR over the period. Period/cohort effects gave the estimations by calculating the ASR in each period/cohort relative to the reference period/cohort. In addition, local drift (%, per year), which could give the estimation of the time trends of ASR, within each age group, was also estimated in this study.

## Results

### Number of cases and ASR for CM from 1990 to 2021

**Figure 1:**
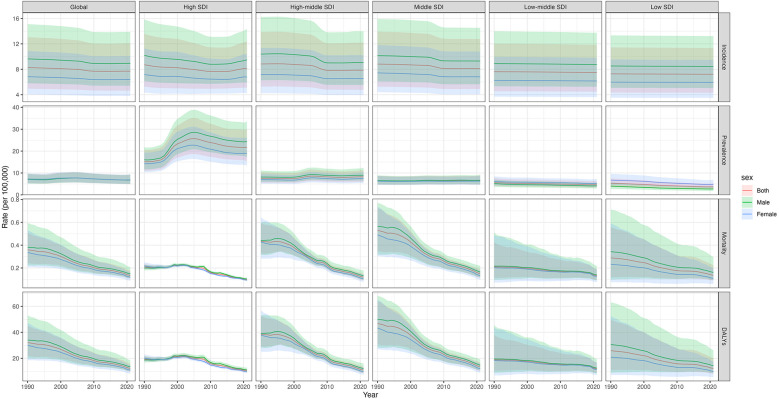
ASR of incidence, prevalence, mortality, and DALYs for childhood myocarditis from 1990 to 2021. ASR, age-standardized rate; DALYs, disability-adjusted life years; SDI, sociodemographic index.

Globally, the ASR of incidence, prevalence, mortality, and DALYs were 7.70 (95% UI: 4.64–12.10), 6.81 (95% UI: 4.90–9.32), 0.13 (95% UI: 0.10–0.18), and 12.24 (95% UI: 9.68–15.97), respectively, per 100,000 population in 2021. The males had a higher ASR of incidence, mortality, and DALYs than that of females, except for prevalence in 1990 and 2021 (Supplementary Tables S1–S4). The highest ASR of incidence and prevalence for CM was found in the high SDI region, while the highest ASR of mortality and DALYs was found in the middle SDI region in 2021 (Figure 1). Correspondingly, the highest ASR of incidence, prevalence, mortality, and DALYs was found in the region of high-income Asia Pacific, high-income North America, and the Caribbean in 2021 (Supplementary Tables S1–S4). As shown in the Supplementary Tables S1–S4, CM burden in 2021 is unequal when considering the distribution of countries/territories. Nationally, a higher ASR of incidence was found in Japan, Brunei Darussalam, and the Republic of Korea. A higher ASR of prevalence for CM was found in Singapore, Canada, and New Zealand. Haiti, Guyana, and Tokelau had a higher ASR of mortality and DALYs. Details on the number of CM cases are shown in Supplementary Tables S1–S4.

### Time trends in ASR for CM from 1990 to 2021

The time trends in ASR of incidence, prevalence, mortality, and DALYs are shown in Figure 1, which indicates a significant decreasing trend for CM burdens from 1990 to 2021 globally. Moreover, we also calculated the EAPC of ASR for CM from 1990 to 2021 (Table 1, Supplementary Table S5). Globally, the ASR of incidence, prevalence, mortality, and DALYs for CM decreased by an average of 0.3% (95%CI: 0.26%–0.33%), 0.16% (0.01%–0.31%), 3.19% (3.05%–3.33%), and 3.13% (2.99%–3.27%), respectively, per year from 1990 to 2021. Additionally, we found that the females had a faster decreasing trend for ASR of prevalence, mortality, and DALYs than the males.

**Table 1:** Estimated average percent change of ASR of incidence, prevalence, mortality, and DALYs for childhood myocarditis from 1990 to 2021.

Characteristics	EAPC, % (95% CI)			
	Incidence	Prevalence	Mortality	DALYs
Global	−0.30 (−0.33 to −0.26)	−0.16 (−0.31 to −0.01)	−3.19 (−3.33 to −3.05)	−3.13 (−3.27 to −2.99)
Sex				
Male	−0.31 (−0.35 to −0.28)	−0.10 (−0.26 to 0.07)	−3.16 (−3.31 to −3.02)	−3.11 (−3.25 to −2.97)
Female	−0.27 (−0.30 to −0.24)	−0.22 (−0.36 to −0.08)	−3.23 (−3.37 to −3.08)	−3.15 (−3.29 to −3.02)
SDI level				
High	−0.33 (−0.42 to −0.23)	1.20 (0.66 to 1.74)	−2.43 (−2.92 to −1.94)	−2.04 (−2.51 to −1.56)
High–middle	−0.54 (−0.62 to −0.46)	0.35 (0.21 to 0.48)	−4.41 (−4.71 to −4.12)	−4.32 (−4.61 to −4.03)
Middle	−0.36 (−0.40 to −0.31)	0.10 (0.07 to 0.13)	−4.13 (−4.33 to −3.94)	−4.10 (−4.28 to −3.91)
Low–middle	−0.05 (−0.06 to −0.05)	−0.56 (−0.59 to −0.53)	−1.23 (−1.36 to −1.10)	−1.23 (−1.36 to −1.11)
Low	−0.02 (−0.03 to −0.02)	−1.29 (−1.33 to −1.25)	−2.41 (−2.53 to −2.29)	−2.41 (−2.53 to −2.29)
Region				
Andean Latin America	0.03 (0.02 to 0.04)	−1.30 (−1.48 to −1.12)	−5.00 (−5.23 to −4.77)	−4.89 (−5.12 to −4.66)
Australasia	−0.09 (−0.11 to −0.06)	−0.57 (−1.52 to 0.39)	−4.24 (−4.84 to −3.63)	−3.76 (−4.37 to −3.14)
Caribbean	0.01 (0.01 to 0.01)	0.64 (0.50 to 0.79)	0.57 (0.45 to 0.69)	0.57 (0.45 to 0.69)
Central Asia	0.02 (0.02 to 0.02)	0.08 (−0.19 to 0.35)	−0.76 (−1.66 to 0.15)	−0.74 (−1.62 to 0.15)
Central Europe	−0.06 (−0.09 to −0.02)	0.42 (0.24 to 0.60)	−4.41 (−4.7 to −4.11)	−4.11 (−4.38 to −3.83)
Central Latin America	0	1.05 (0.94 to 1.16)	1.20 (0.88 to 1.51)	1.17 (0.87 to 1.48)
Central sub-Saharan Africa	0	−1.36 (−1.45 to −1.28)	−3.13 (−3.33 to −2.92)	−3.15 (−3.35 to −2.94)
East Asia	−0.93 (−1.08 to −0.78)	1.01 (0.87 to 1.15)	−4.56 (−4.93 to −4.18)	−4.53 (−4.90 to −4.16)
Eastern Europe	0.01 (0.01 to 0.01)	−1.19 (−1.40 to −0.97)	−2.87 (−3.74 to −1.98)	−2.74 (−3.55 to −1.93)
Eastern sub-Saharan Africa	−0.02 (−0.02 to −0.02)	−2.54 (−2.68 to −2.40)	−3.90 (−4.00 to −3.80)	−3.90 (−4.00 to −3.81)
High-income Asia Pacific	0.27 (0.24 to 0.30)	2.07 (1.60 to 2.53)	−3.19 (−3.33 to −3.05)	−2.11 (−2.57 to −1.64)
High-income North America	−0.71 (−0.96 to −0.45)	0.83 (0.37 to 1.29)	−2.64 (−3.14 to −2.14)	−1.6 (−2.04 to −1.17)
North Africa and the Middle East	−0.06 (−0.06 to −0.05)	−0.45 (−0.51 to −0.39)	−1.92 (−2.37 to −1.47)	−2.72 (−2.93 to −2.51)
Oceania	0	−0.07 (−0.08 to −0.06)	−2.75 (−2.97 to −2.54)	0.07 (0.01 to 0.12)
South Asia	0.01 (0.01 to 0.01)	−0.44 (−0.49 to −0.40)	−0.69 (−0.87 to −0.51)	−0.7 (−0.87 to −0.52)
South-East Asia Region	0.03 (0.02 to 0.03)	−0.13 (−0.18 to −0.07)	−1.49 (−1.61 to −1.37)	−1.48 (−1.60 to −1.37)
Southern Latin America	−0.08 (−0.08 to −0.07)	−1.03 (−1.31 to −0.74)	−5.67 (−6.15 to −5.20)	−5.53 (−5.99 to −5.07)
Southern sub-Saharan Africa	0 (−0.01 to 0)	−0.52 (−0.72 to −0.32)	−1.23 (−1.40 to −1.06)	−1.22 (−1.39 to −1.05)
Tropical Latin America	0	0.36 (0.18 to 0.53)	−2.04 (−2.64 to −1.42)	−1.98 (−2.58 to −1.39)
Western Europe	−0.15 (−0.21 to −0.08)	1.59 (0.68 to 2.50)	−3.17 (−4.14 to −2.19)	−2.50 (−3.43 to −1.56)
Western sub-Saharan Africa	0.01 (0 to 0.01)	−0.69 (−0.74 to −0.65)	−2.80 (−3.04 to −2.56)	−2.77 (−3.00 to −2.54)

ASR, age-standardized rate; DALY, disability-adjusted life years; EAPC, estimated average percent change; SDI, sociodemographic index.

Importantly, we observed that ASR of prevalence presented an increasing trend in the high, high–middle, and middle SDI regions and a decreasing trend in the low–middle and low SDI regions. Other metrics also presented a decreasing trend regardless of global region or different SDI regions. Regionally, the most significant decreases in ASR of mortality and DALYs were observed in Southern Latin America, while the most significant increases in ASR of mortality and DALYs were observed in Central Latin America. The most significant decreases in ASR of incidence and prevalence were observed in East Asia and Eastern sub-Saharan Africa, respectively, while the most significant increases in ASR of incidence and prevalence were observed in high-income Asia Pacific (Figure 1, Table 1**)**.

**Figure 2:**
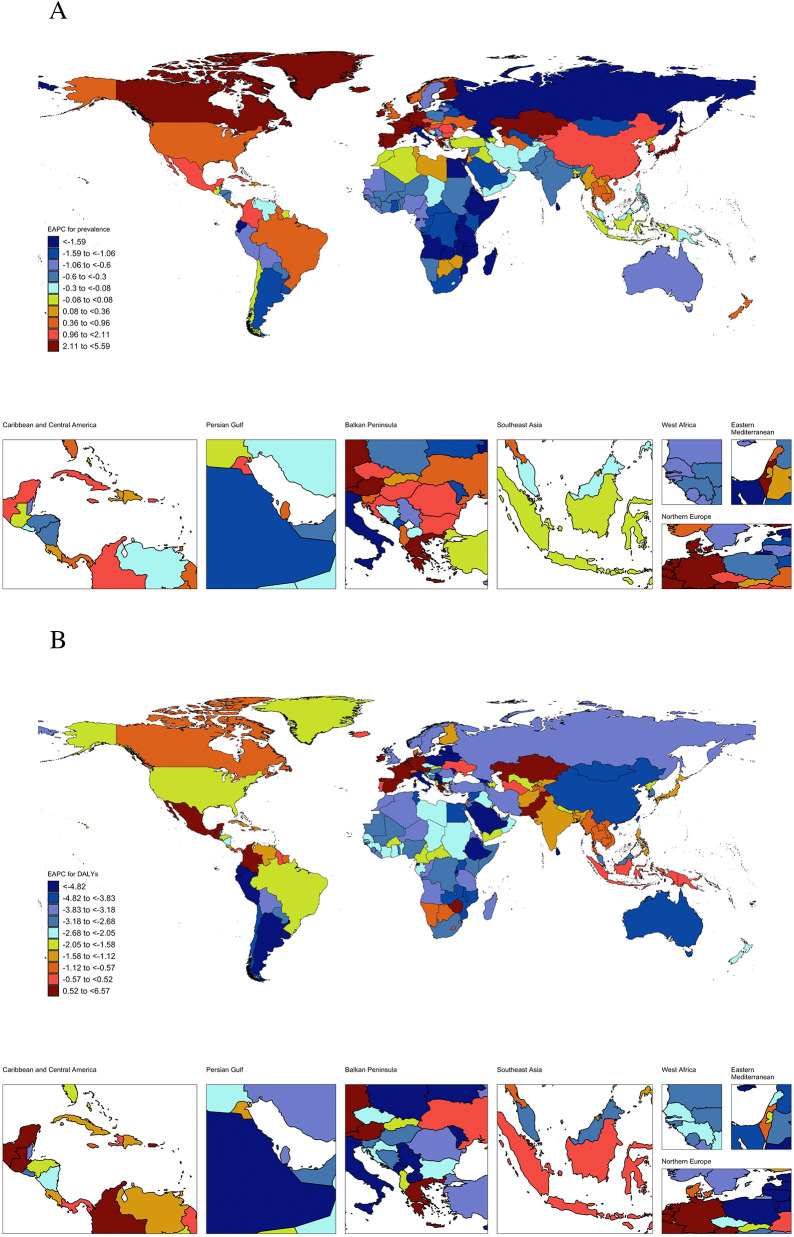
Estimated average percent change of ASR of prevalence **(A)** and DALYs **(B)** for childhood myocarditis from 1990 to 2021 by country or territories (ASR, age-standardized rate; DALY, disability-adjusted life years).

Nationally, CM burden varied significantly across 204 countries and territories (Figure 2, Supplementary Figure S2, Supplementary Table S5). The most significant decrease in ASR of incidence was observed in China, while the most significant increase in ASR of incidence was observed in Taiwan (China). The most significant decrease in ASR of prevalence was observed in Ethiopia, while the most significant increase in ASR of prevalence was observed in Ireland. The most significant decreases in EAPC of mortality and DALYs were observed in Estonia, while the most significant increases in ASR of mortality and DALYs were observed in Mauritius.

### Cross-region and cross-country inequality analysis for CM

**Figure 3:**
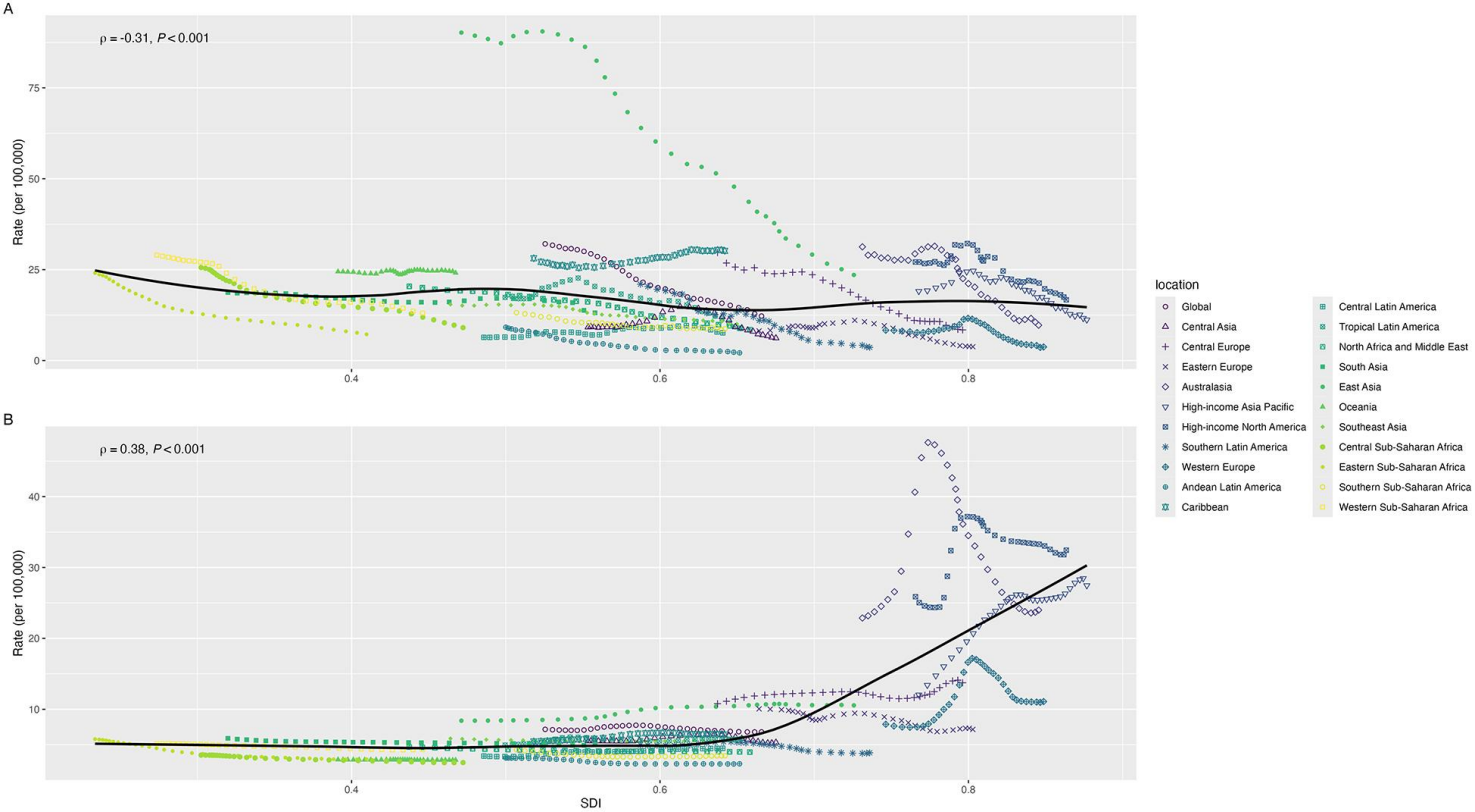
The associations of SDI with ASR of DALYs **(A)** and prevalence **(B)** for childhood myocarditis from 1990 to 2021 by regions (ASR: age-standardized rate, DALYs: disability-adjusted life years, SDI: sociodemographic index).

**Figure 4:**
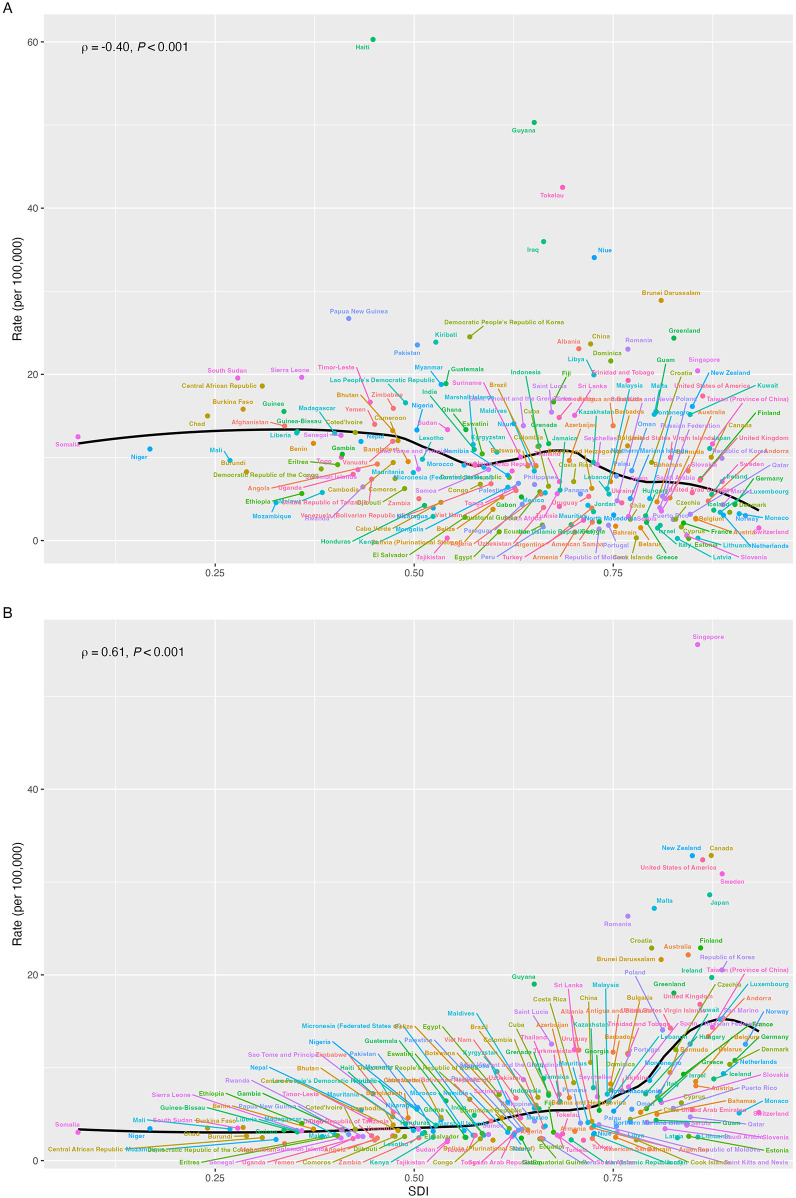
The associations of SDI with ASR of DALYs **(A)** and prevalence **(B)** and for childhood myocarditis from 1990 to 2021 by countries or territories (ASR: age-standardized rate, DALYs: disability-adjusted life years, SDI: sociodemographic index).

Regionally, we found various associations of SDI values with the ASR of incidence, prevalence, mortality, and DALYs of CM (Figure 3, Supplementary Figure S3). The ASR of DALYs fluctuatingly decreased as the SDI values increased, with a slightly negative correlation index (*ρ* = −0.42, *P* < 0.001). The ASR of prevalence was initially stable until the SDI value was approximately 0.6 and kept increasing as SDI values increased; the correlation relationship was statistically significant (*ρ* = 0.38, *P* < 0.001). Conversely, the ASR of mortality decreased as SDI values increased (*ρ* = −0.35, *P* < 0.001). Although there was a positive association between the SDI values and the ASR of incidence, we did not find the trend statistically significant (*ρ* < 0.001, *P* = 0.795). The ASR of incidence, prevalence, mortality, and DALYs for CM among different regions mostly presented a similar trend from 1990 to 2021 as SDI values increased. Nationally, we also found similar trends that SDI values were negatively associated with the ASR of mortality and DALYs and positively associated with the ASR of incidence and prevalence of CM (Figure 4, Supplementary Figure S4).

**Figure 5:**
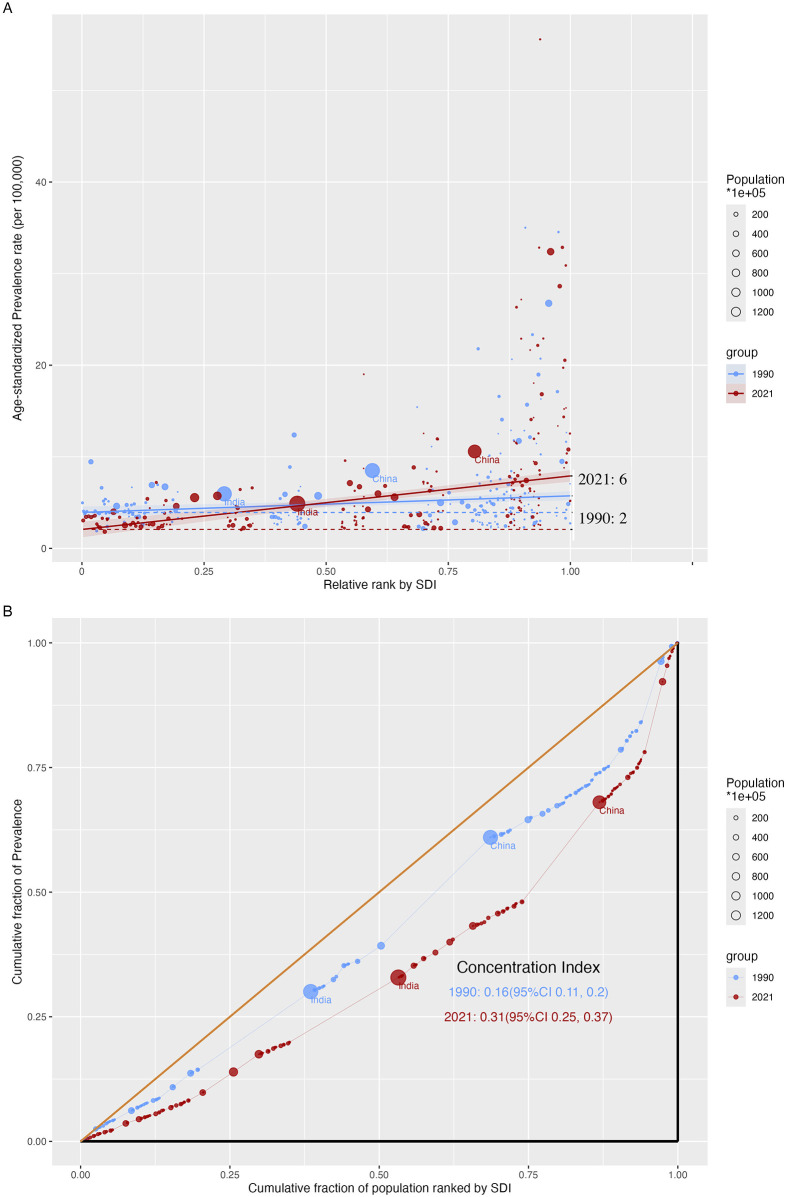
SDI-related health inequality regression **(A)** and concentration **(B)** curves for ASR of prevalence of childhood myocarditis from 1990 to 2021 (ASR, age-standardized rate; SDI, sociodemographic index).

We found absolute and relative SDI-related health inequality in CM burden (Figure 5, Supplementary Figures S5–S7). Higher ASR of prevalence were disproportionately distributed in countries/territories with high SDI values. Other metrics, namely, ASR of incidence, mortality, and DALYs, were nearly uniformly distributed as the SDI values increased. Figure 5A shows that there was a higher excess of ASR of prevalence in 2021 than in 1990, indicating a higher SDI-related health inequality for CM. However, lower SDI-related health inequality was found in the ASR of DALYs as lower excesses were found in 2021 than in 1990 (Supplementary Figure S6). Moreover, relative gradient inequality, namely, the imbalanced distribution of ASR of prevalence among 204 countries/territories with different SDI, was found in the present study, with both significant concentration index in 1990 (0.16, 95% CI: 0.11–0.20) and 2021 (0.31, 95% CI: 0.25–0.37) (Figure 5B). A relatively lower inequality was also found in the ASR of incidence for CM, but not significantly for mortality and DALYs.

### APC analysis for CM

**Figure 6:**
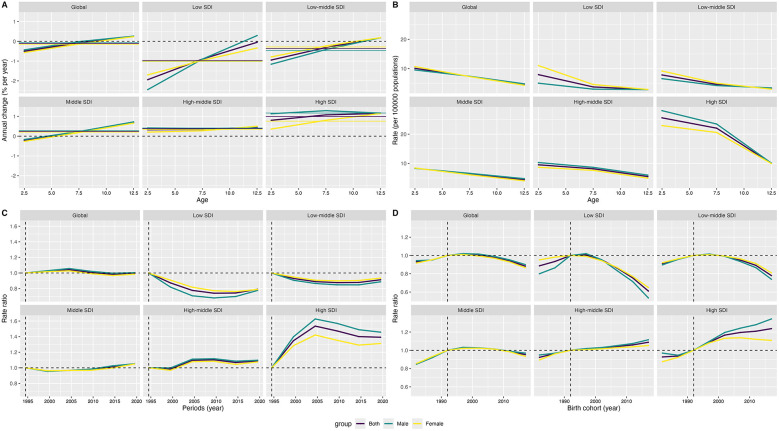
Local drifts **(A)**, age **(B)**, period **(C)** and cohort **(D)** effects on prevalence rate of childhood myocarditis from 1990 to 2021 (SDI: sociodemographic index).

The annual changes in the prevalence, DALYs, mortality, and incidence within each age group for CM and local drifts are shown in Figure 6A and Supplementary Figures S8A, S9A, and S10A. Globally, the changes in prevalence for CM increased as age increased, and similar trends were also found in different SDI regions. The changes in DALYs and mortality shared similar increasing trends both worldwide and in different SDI regions. Differently, the annual changes in incidence for CM were stable in the global region, and decreasing trends were found in the high–middle and high SDI regions, indicating the annual change was larger among older children.

Figures 6B–D and Supplementary Figures S8B–D, S9B–D, and S10B–D show the effect of age, period, and birth cohort on the prevalence, DALYs, mortality, and incidence of CM. The prevalence, mortality, and DALYs decreased as age increased in the global region and different SDI regions; children aged 0–4 years had a higher prevalence, mortality, and DALYs related to CM than those aged 5–14 years. Differently, an increasing trend for incidence of CM was found in the global region and low, low–middle, and middle SDI regions. However, as age increased, the trend of incidence initially decreased and then increased in the high–middle and high SDI regions. Interestingly, the females, who had a decreasing trend of incidence in the high–middle and high SDI regions, were different from the males and the total populations under 14 years old. From the perspective of periods, compared with the earliest period (1992 to 1996), recent periods had an elevated risk in the high SDI regions and a decreased risk in the low SDI regions for prevalence of CM. When focusing on mortality, DALYs, and incidence, the recent periods all had a decreased risk in global and different SDI regions. From the perspective of birth cohort, the risk of prevalence of CM initially increased and then decreased in the global region, as well as the low, low–middle, and middle SDI regions. An increasing risk of prevalence of CM was found in the high–middle and high SDI regions. As for incidence, DALYs, and mortality, the risk was almost decreasing globally, and different regions by SDI share similar trends.

### Risk factors for CM

Analyses of risk factors for CM were performed, and non-optimal temperature including low and high temperature were found to be associated with the ASR of DALYs (Supplementary Table S6) and mortality (Supplementary Table S7). In 2021, low temperature, high temperature, and non-optimal temperature accounted for 0.55, 0.32, and 0.84, respectively, per 100,000 population for ASR of DALYs. The corresponding EAPC of ASR of DALYs from 1990 to 2021 for non-optimal temperature, low temperature, and high temperature were −3.20%, −3.98%, and −1.06%, respectively. Similar trends were also found in the ASR of mortality. Interestingly, the EAPC of ASR of DALYs and mortality was increasing in the low–middle and low SDI regions related to high temperature.

## Discussion

### Clinical implications and conclusions

Using the most up-to-date database from GBD 2021, updated in 2024, the present study systematically and firstly reported the incidence, prevalence, mortality, and DALYs and further explored health inequality and the effect of age, period, and cohort effect on CM burden. Under the decreasing trend from 1990 to 2021 for CM burdens globally, different regions or countries/territories showed various trends. The increased EAPC of prevalence for CM remained higher in high SDI regions than in other regions, which should be paid enough attention to. SDI-related health inequality across countries/territories and regions was significant for CM burden in 2021. Children aged 0–4 years had a higher prevalence, mortality, and DALYs related to CM than those aged 5–14 years. A higher risk for CM prevalence was found in the younger ages, recent periods, and birth cohort among high SDI regions. Non-optimal temperature should also be used with caution to prevent excess DALYs and mortality. Considering the huge effect of CM on adult health, necessary and specific interventions should be performed worldwide.

### Age and differences in CM burden

APC models in the present study gave some information that the prevalence, mortality, and DALYs rates of CM were higher in the younger population. These findings align with prior research focusing on myocarditis and cardiomyopathy in children, taking advantage of GBD 2019 data (5). However, the global incidence rate for CM was higher in the older pediatric age groups, potentially reflecting a better prognosis. The clinicians should prioritize rigorous monitoring of younger children with myocarditis, given their disproportionately elevated mortality risk.

In the present study, we found a higher incidence, mortality, and DALYs of CM in males, but a steeper EAPC decline of prevalence, mortality, DALYs, and rate in females. This evidence indicated that the males were at a high position for CM burden with lower EAPC. Although previous limited evidence from clinics (26) also supports the findings in this study, we obtained enough materials for explaining the inequality between sex, and more studies are needed to explore the differences. Previous studies in adults attributed the differences in burden for sex hormones (27), and the effect of hormones on CM remains unclear, considering not enough differences between males and females under 14 years. Nevertheless, the findings for CM burden between different sexes give some evidence for clinics.

### SDI-related differences in CM burden

SDI-related health inequality analyses had emphasized that higher ASR of prevalence and incidence and lower ASR of mortality and DALYs were significantly associated with higher SDI levels. The EAPC also shared similar trends with ASR in the present study. A previous study also mentioned the trends in the burdens of myocarditis and cardiomyopathy in children (5). The main reason for explaining the trends is the development of improved diagnostics, healthcare access, and management for CM in recent years (2, 4, 28, 29). Effective screening could bring more cases, and better management is an effective method for decreasing the mortality, which is attributed to downward DALYs. Moreover, environmental factors such as air pollution, particularly provided by automobile exhaust, might be related to higher incidence and prevalence of CM in the region with higher SDI levels. Globally, more actions should be performed in the low SDI regions to increase the accuracy rate of diagnoses and decrease mortality related to CM.

### Period and cohort effect

The present study found that a higher risk of prevalence and a lower risk of incidence, mortality, and DALYs in recent period and birth cohort among high SDI regions. These results indicated that the amount in storage for CM cases was larger in higher SDI regions and that more valid treatment was needed for curing CM. Additionally, from the perspective of the global region and low SDI region, the risk of all the indices of CM burden was lower for recent period and cohort, indicating an improving situation for CM diagnosis and treatment. More studies are needed to verify the details of CM burden considering different reasons for CM related to different SDI (4).

### Risk factors in CM burden

Previous studies have reported that non-optimal temperature was highly associated with increased risk of myocarditis-specific mortality in the whole age groups (30). This study added more evidence that non-optimal temperature was also significantly associated with elevated risk of mortality and DALYs for CM. An epidemiological study from China also reported that ambient temperature was related to hospitalization for cardiovascular diseases among the population aged under 4 years (31). However, limited evidence from animals has been reported until now, and more studies are needed to explore the mechanism of non-optimal temperature with CM.

### Strengths and limitations

This is the first study to use up-to-date GBD data to explore the global, regional, and national CM burden from 1990 to 2021. SDI-related inequality and APC models provided us with more hints for the management of CM. Robust design, large sample size, and complicated methods for GBD 2021 could give more accurate and recent information. Some limitations should be mentioned when expanding the results. The underreported cases in underdeveloped countries/territories might affect the estimates of CM burden and affect the trend comparisons. Although GBD 2021 adopted a series of models to generate data globally, these estimations might not have a good representation of real-world conditions. Although GBD 2021 adopted a scientific method for estimating the burden of diseases, some potential biases of different levels of diagnosis and different medical policies might affect the cross-country comparisons.

## Conclusions

The present study offers a comprehensive finding for CM burden. Although CM burden has been decreasing in recent years, the disproportions of CM burden globally should be approached with caution. High SDI regions should formulate detailed measures and strategies to manage cases, and low SDI regions should adopt more methods to decrease the mortality for CM.

## Data Availability

The datasets presented in this study can be found in online repositories. The names of the repository/repositories and accession number(s) can be found below: http://ghdx.healthdata.org/gbd-results-tool.
