# Supplementary material for: Global, regional, and national trends for childhood myocarditis from 1990 to 2021: health inequality and age-period-cohort analysis for the Global Burden of Disease Study 2021
Source: Front Pediatr. 2025 Dec 5;13:1566010. doi: 10.3389/fped.2025.1566010 (PMC12714953; doi:10.3389/fped.2025.1566010)
Supplement: Supplementary file 1 [file Supplementaryfile1.docx]

**Additional files**

**Table S1:** Number cases and ASR of incidence for childhood myocarditis from 1990 to 2021 by SDI level, region, sex and countries/territories

**Table S2:** Number cases and ASR of prevalence for childhood myocarditis from 1990 to 2021 by SDI level, region, sex and countries/territories

**Table S3:** Number cases and ASR of mortality for childhood myocarditis from 1990 to 2021 by SDI level, region, sex and countries/territories

**Table S4:** Number cases and ASR of DALYs for childhood myocarditis from 1990 to 2021 by SDI level, region, sex and countries/territories

**Table S5:** Estimated average percent change of ASR of incidence, prevalence, mortality and DALYs for childhood myocarditis from 1990 to 2021 by countries/territories

**Table S6:** Main risk factors for estimated average percent change of ASR of DALYs for childhood myocarditis from 1990 to 2021

**Table S7:** Main risk factors for estimated average percent change of ASR of mortality for childhood myocarditis from 1990 to 2021

**Figure S1**: Workflow diagram for global burden of childhood myocarditis

**Figure S2:** Estimated average percent change of ASR of incidence (A) and mortality (B) for childhood myocarditis from 1990 to 2021 by countries/territories

**Figure S3:** The associations of SDI with ASR of incidence (A) and mortality (B) for childhood myocarditis from 1990 to 2021 by regions

**Figure S4:** The associations of SDI with ASR of incidence (A) and mortality (B) in 2021 for childhood myocarditis by countries/territories

**Figure S5:** SDI-related health inequality regression (A) and concentration (B) curves for ASR of mortality of childhood myocarditis from 1990 to 2021

**Figure S6:** SDI-related health inequality regression (A) and concentration (B) curves for ASR of DALYs of childhood myocarditis from 1990 to 2021

**Figure S7:** SDI-related health inequality regression (A) and concentration (B) curves for ASR of incidence of childhood myocarditis from 1990 to 2021

**Figure S8:** Local drifts (A), age (B), period (C) and cohort (D) effects on DALYs rate of childhood myocarditis from 1990 to 2021

**Figure S9:** Local drifts (A), age (B), period (C) and cohort (D) effects on mortality rate of childhood myocarditis from 1990 to 2021

**Figure S10:** Local drifts (A), age (B), period (C) and cohort (D) effects on incidence rate of childhood myocarditis from 1990 to 2021

**Table S1: Number cases and ASR of incidence for childhood myocarditis from 1990 to 2021 by SDI level, region, sex and countries/territories**

| **Characteristics** | **Number of cases (per 1,000) (95% UI)** | | **ASR (per 100, 000) (95% UI)** | |
| --- | --- | --- | --- | --- |
|  | **1990** | **2021** | **1990** | **2021** |
| **Global** | 143.8 (93.13 to 214.67) | 155.45 (100.31 to 232.31) | 8.28 (4.95 to 13.06) | 7.7 (4.64 to 12.10) |
| **Age (years)** |  |  |  |  |
| **<5** | 47.64 (33.12 to 67.55) | 46.8 (32.64 to 66.14) |  |  |
| **5-9** | 47 (26.83 to 79.61) | 51.43 (29.39 to 86.82) |  |  |
| **10-14** | 49.16 (26.26 to 79.57) | 57.22 (31.12 to 91.99) |  |  |
| **Sex** |  |  |  |  |
| Male | 85.98 (55.87 to 127.67) | 93.18 (60.26 to 138.77) | 9.64 (5.8 to 15.11) | 8.94 (5.41 to 13.9) |
| Female | 57.82 (37.17 to 86.83) | 62.27 (39.63 to 93.81) | 6.84 (4.05 to 10.89) | 6.38 (3.8 to 10.09) |
| **SDI level** |  |  |  |  |
| High | 16.22 (10.59 to 23.75) | 14.1 (9.58 to 20.15) | 8.72 (5.27 to 13.57) | 8.17 (5.1 to 12.39) |
| High-middle | 24.22 (15.61 to 35.73) | 18.17 (11.87 to 26.88) | 8.84 (5.28 to 13.87) | 7.86 (4.78 to 12.19) |
| Middle | 50.97 (32.86 to 76.29) | 46.2 (29.56 to 69.47) | 8.82 (5.27 to 13.93) | 8.11 (4.89 to 12.77) |
| Low-middle | 35.78 (23.01 to 53.84) | 43.69 (27.65 to 66.31) | 7.61 (4.55 to 12.03) | 7.5 (4.48 to 11.85) |
| Low | 16.5 (10.69 to 24.53) | 33.17 (21.27 to 49.63) | 7.28 (4.34 to 11.44) | 7.22 (4.31 to 11.35) |
| **Region** |  |  |  |  |
| Andean Latin America | 0.95 (0.61 to 1.43) | 1.17 (0.75 to 1.75) | 6.41 (3.79 to 10.11) | 6.44 (3.84 to 10.16) |
| Australasia | 0.31 (0.2 to 0.46) | 0.39 (0.25 to 0.57) | 6.82 (4.08 to 10.70) | 6.74 (4.05 to 10.53) |
| Caribbean | 0.76 (0.49 to 1.16) | 0.77 (0.49 to 1.18) | 6.66 (3.91 to 10.67) | 6.67 (3.92 to 10.68) |
| Central Asia | 1.97 (1.28 to 2.97) | 2.2 (1.42 to 3.34) | 7.94 (4.72 to 12.58) | 7.98 (4.75 to 12.65) |
| Central Europe | 2.4 (1.57 to 3.5) | 1.44 (0.95 to 2.11) | 8.09 (4.90 to 12.46) | 8.12 (4.91 to 12.50) |
| Central Latin America | 4.55 (2.89 to 6.85) | 4.52 (2.81 to 6.86) | 7.06 (4.18 to 11.25) | 7.06 (4.18 to 11.23) |
| Central Sub-Saharan Africa | 1.73 (1.13 to 2.53) | 4.06 (2.63 to 6.01) | 6.92 (4.12 to 10.85) | 6.93 (4.12 to 10.86) |
| East Asia | 34.04 (21.77 to 50.2) | 22.28 (14.53 to 32.91) | 10.31 (6.10 to 16.27) | 8.35 (5.09 to 12.91) |
| Eastern Europe | 4.52 (2.9 to 6.91) | 3.15 (2 to 4.89) | 8.77 (5.25 to 13.73) | 8.8 (5.26 to 13.79) |
| Eastern Sub-Saharan Africa | 6.54 (4.25 to 9.7) | 12.93 (8.3 to 19.33) | 7.29 (4.35 to 11.46) | 7.25 (4.33 to 11.41) |
| High-income Asia Pacific | 4.28 (2.8 to 6.32) | 2.89 (1.92 to 4.24) | 12.17 (7.51 to 18.88) | 12.98 (8.11 to 20.03) |
| High-income North America | 4.24 (2.64 to 6.48) | 4.09 (2.83 to 5.76) | 6.88 (3.99 to 10.96) | 6.14 (3.84 to 9.20) |
| North Africa and Middle East | 7.6 (4.79 to 11.63) | 9.85 (6.14 to 15.25) | 5.44 (3.18 to 8.65) | 5.35 (3.13 to 8.55) |
| Oceania | 0.25 (0.17 to 0.37) | 0.48 (0.32 to 0.7) | 9.37 (5.60 to 14.51) | 9.37 (5.60 to 14.51) |
| South Asia | 33.45 (21.45 to 50.81) | 39.8 (24.97 to 60.88) | 7.75 (4.61 to 12.24) | 7.78 (4.63 to 12.28) |
| South-East Asia Region | 17.07 (11.15 to 25.6) | 17.4 (11.3 to 26.27) | 9.98 (6.06 to 15.58) | 10.04 (6.08 to 15.65) |
| Southern Latin America | 0.9 (0.59 to 1.33) | 0.86 (0.56 to 1.27) | 6.05 (3.60 to 9.47) | 5.93 (3.60 to 9.12) |
| Southern Sub-Saharan Africa | 1.55 (0.99 to 2.33) | 1.81 (1.15 to 2.75) | 7.50 (4.47 to 11.8) | 7.5 (4.47 to 11.78) |
| Tropical Latin America | 4.02 (2.49 to 6.16) | 3.75 (2.34 to 5.71) | 7.45 (4.39 to 11.94) | 7.45 (4.39 to 11.94) |
| Western Europe | 6.28 (4.29 to 8.96) | 5.9 (4.06 to 8.37) | 8.92 (5.53 to 13.67) | 8.77 (5.50 to 13.32) |
| Western Sub-Saharan Africa | 6.37 (4.16 to 9.43) | 15.71 (10.1 to 23.47) | 7.33 (4.38 to 11.52) | 7.35 (4.39 to 11.55) |
| **Countries or territories** |  |  |  |  |
| Afghanistan | 227 (143.9 to 348.22) | 742.68 (467.11 to 1149.23) | 5.31 (3.11 to 8.52) | 5.29 (3.10 to 8.49) |
| Albania | 83.6 (56.2 to 120.7) | 33.36 (22.22 to 48.56) | 7.49 (4.55 to 11.52) | 7.50 (4.56 to 11.53) |
| Algeria | 564.88 (352.48 to 880.97) | 697.34 (434.89 to 1088.43) | 5.28 (3.09 to 8.47) | 5.26 (3.08 to 8.45) |
| American Samoa | 1.74 (1.16 to 2.56) | 1.31 (0.84 to 1.99) | 9.20 (5.49 to 14.28) | 9.17 (5.46 to 14.2) |
| Andorra | 0.78 (0.52 to 1.14) | 0.82 (0.55 to 1.2) | 8.42 (5.24 to 12.81) | 8.37 (5.21 to 12.74) |
| Angola | 322.96 (211.61 to 470.02) | 1048.89 (679.41 to 1549.01) | 6.92 (4.11 to 10.84) | 6.91 (4.11 to 10.83) |
| Antigua and Barbuda | 1.21 (0.77 to 1.86) | 1.13 (0.72 to 1.75) | 6.62 (3.90 to 10.62) | 6.63 (3.90 to 10.63) |
| Argentina | 574.2 (368.45 to 849.61) | 578.42 (366.61 to 877.23) | 5.66 (3.34 to 8.87) | 5.66 (3.35 to 8.87) |
| Armenia | 92.34 (60.14 to 134.96) | 53.44 (34.1 to 79.72) | 8.89 (5.33 to 13.9) | 8.98 (5.36 to 14.01) |
| Australia | 253.93 (164.84 to 370.53) | 318.81 (205.45 to 468.62) | 6.70 (4.03 to 10.48) | 6.70 (4.03 to 10.49) |
| Austria | 125.23 (91.88 to 167.62) | 122.3 (85.57 to 171.25) | 9.36 (6.25 to 13.65) | 9.49 (6.04 to 14.25) |
| Azerbaijan | 192.58 (124.28 to 292.11) | 191.18 (120.93 to 295.58) | 7.97 (4.74 to 12.64) | 8.05 (4.79 to 12.76) |
| Bahamas | 5.37 (3.4 to 8.3) | 5.48 (3.44 to 8.57) | 6.62 (3.89 to 10.62) | 6.62 (3.90 to 10.60) |
| Bahrain | 8.49 (5.32 to 13.2) | 15.76 (9.77 to 24.61) | 5.26 (3.08 to 8.45) | 5.26 (3.08 to 8.45) |
| Bangladesh | 3373.64 (2188.9 to 4958.09) | 3194.98 (2020.67 to 4776.21) | 6.95 (4.13 to 10.88) | 6.94 (4.13 to 10.87) |
| Barbados | 4.16 (2.64 to 6.43) | 3.17 (2 to 4.91) | 6.62 (3.89 to 10.62) | 6.64 (3.90 to 10.63) |
| Belarus | 186.74 (121.64 to 283.01) | 123.1 (78.94 to 188.07) | 7.76 (4.68 to 12.23) | 7.77 (4.68 to 12.24) |
| Belgium | 129.96 (88.01 to 184.46) | 136.35 (92.12 to 194.84) | 7.25 (4.52 to 11.01) | 7.24 (4.51 to 10.99) |
| Belize | 5.41 (3.45 to 8.29) | 8.22 (5.2 to 12.7) | 6.62 (3.89 to 10.62) | 6.62 (3.89 to 10.61) |
| Benin | 166.64 (108.78 to 243.56) | 418.67 (271.73 to 615.21) | 6.96 (4.14 to 10.90) | 6.93 (4.12 to 10.86) |
| Bermuda | 0.79 (0.5 to 1.21) | 0.57 (0.36 to 0.87) | 6.62 (3.89 to 10.62) | 6.63 (3.90 to 10.62) |
| Bhutan | 18.23 (11.83 to 26.89) | 13.03 (8.28 to 19.39) | 6.96 (4.14 to 10.9) | 6.92 (4.12 to 10.85) |
| Bolivia (Plurinational State of) | 172.7 (111.56 to 261.12) | 225.5 (143.64 to 344.44) | 6.46 (3.82 to 10.25) | 6.46 (3.82 to 10.26) |
| Bosnia and Herzegovina | 82.01 (54.53 to 119.42) | 36.75 (24.41 to 53.67) | 7.47 (4.54 to 11.49) | 7.47 (4.54 to 11.48) |
| Botswana | 40.72 (26.39 to 60.18) | 48.53 (31.04 to 72.14) | 6.91 (4.11 to 10.83) | 6.93 (4.12 to 10.86) |
| Brazil | 3912.77 (2419.18 to 5990.48) | 3615.17 (2259.23 to 5513.81) | 7.48 (4.40 to 11.98) | 7.49 (4.41 to 12.00) |
| Brunei Darussalam | 9.85 (6.79 to 13.93) | 10.25 (6.95 to 14.69) | 10.84 (6.69 to 16.65) | 10.86 (6.70 to 16.67) |
| Bulgaria | 130.39 (86.88 to 190.03) | 73.22 (48.72 to 106.76) | 7.47 (4.54 to 11.48) | 7.47 (4.54 to 11.49) |
| Burkina Faso | 326.93 (213.46 to 477.27) | 712.34 (463.68 to 1042.99) | 6.98 (4.15 to 10.94) | 6.92 (4.12 to 10.84) |
| Burundi | 179.34 (117.22 to 261.69) | 403.02 (261.5 to 594.69) | 6.92 (4.11 to 10.84) | 6.91 (4.11 to 10.83) |
| Cabo Verde | 10.83 (7.01 to 15.96) | 10 (6.32 to 14.97) | 6.92 (4.12 to 10.85) | 6.93 (4.12 to 10.86) |
| Cambodia | 432.61 (286.18 to 638.27) | 479.88 (311.72 to 714.52) | 9.33 (5.65 to 14.48) | 9.37 (5.68 to 14.53) |
| Cameroon | 335.69 (219.43 to 489.47) | 932.16 (604.14 to 1378.55) | 6.95 (4.13 to 10.88) | 6.94 (4.13 to 10.87) |
| Canada | 354.69 (236.02 to 529.44) | 383.3 (253.27 to 578.58) | 6.14 (3.68 to 9.68) | 6.14 (3.68 to 9.68) |
| Central African Republic | 83.86 (54.85 to 122.23) | 157.86 (102.51 to 232.82) | 6.93 (4.12 to 10.86) | 6.93 (4.12 to 10.86) |
| Chad | 200.41 (131.98 to 290.73) | 618.68 (403.43 to 904.46) | 6.93 (4.12 to 10.86) | 6.93 (4.12 to 10.86) |
| Chile | 277.99 (184.55 to 414.1) | 240.58 (164.51 to 340.85) | 7.00 (4.21 to 10.88) | 6.59 (4.20 to 9.81) |
| China | 33088.61 (21137.16 to 48844.7) | 21603.18 (14078.21 to 31928.87) | 10.38 (6.14 to 16.41) | 8.34 (5.07 to 12.90) |
| Colombia | 771.6 (493.51 to 1181.41) | 708.42 (449.88 to 1092.2) | 6.62 (3.89 to 10.62) | 6.63 (3.90 to 10.63) |
| Comoros | 14.67 (9.55 to 21.51) | 16.7 (10.69 to 24.82) | 6.94 (4.13 to 10.87) | 6.94 (4.13 to 10.87) |
| Congo | 72.5 (47.12 to 106.7) | 134.07 (85.29 to 199.78) | 6.91 (4.11 to 10.83) | 6.92 (4.12 to 10.85) |
| Cook Islands | 0.61 (0.39 to 0.91) | 0.35 (0.22 to 0.52) | 9.17 (5.46 to 14.21) | 9.14 (5.45 to 14.17) |
| Costa Rica | 74.39 (47.55 to 114.08) | 68.03 (42.86 to 105.41) | 6.63 (3.9 to 10.63) | 6.63 (3.91 to 10.62) |
| Cote d’Ivoire | 392.52 (256.2 to 573.4) | 800.64 (519.1 to 1179.62) | 6.95 (4.14 to 10.89) | 6.95 (4.14 to 10.89) |
| Croatia | 67.7 (43.88 to 99.58) | 39.78 (27.08 to 56.35) | 6.84 (4.10 to 10.58) | 6.63 (4.15 to 9.95) |
| Cuba | 166.36 (106.36 to 254.94) | 118.93 (75.03 to 184.18) | 6.64 (3.90 to 10.64) | 6.64 (3.91 to 10.64) |
| Cyprus | 17.22 (11.93 to 23.88) | 19.16 (13.32 to 26.44) | 8.79 (5.53 to 13.39) | 8.78 (5.53 to 13.38) |
| Czechia | 183.78 (121.66 to 275.28) | 142.43 (95.45 to 211.44) | 8.27 (5.02 to 12.82) | 8.27 (5.02 to 12.82) |
| Democratic People's Republic of Korea | 497.34 (340.32 to 712.63) | 399.23 (266.34 to 580.71) | 8.34 (5.10 to 12.85) | 8.36 (5.11 to 12.87) |
| Democratic Republic of the Congo | 1213.56 (793.91 to 1768.44) | 2631.04 (1701.92 to 3895.74) | 6.92 (4.12 to 10.85) | 6.93 (4.13 to 10.86) |
| Denmark | 68.56 (47.42 to 98) | 73.99 (51.15 to 105.7) | 7.83 (4.87 to 12.04) | 7.82 (4.87 to 12.03) |
| Djibouti | 12.13 (7.89 to 17.84) | 28.96 (18.69 to 42.91) | 6.98 (4.15 to 10.93) | 7.01 (4.17 to 10.96) |
| Dominica | 1.65 (1.06 to 2.54) | 0.92 (0.58 to 1.45) | 6.65 (3.91 to 10.67) | 6.63 (3.91 to 10.63) |
| Dominican Republic | 177.83 (113.86 to 271.3) | 194.86 (124.43 to 299.46) | 6.61 (3.88 to 10.6) | 6.63 (3.9 to 10.63) |
| Ecuador | 243.35 (157.35 to 363.26) | 324.27 (208.74 to 477.37) | 6.29 (3.74 to 9.79) | 6.37 (3.81 to 9.98) |
| Egypt | 1156.94 (726.9 to 1790.93) | 1935.09 (1206.88 to 3016.86) | 5.26 (3.08 to 8.45) | 5.27 (3.09 to 8.46) |
| El Salvador | 143 (91.43 to 219.06) | 121.11 (76.92 to 186.94) | 6.63 (3.89 to 10.63) | 6.64 (3.91 to 10.64) |
| Equatorial Guinea | 13.52 (8.88 to 19.67) | 41.44 (26.25 to 61.95) | 6.94 (4.13 to 10.88) | 7.04 (4.18 to 11.00) |
| Eritrea | 110.1 (71.61 to 161.31) | 175.17 (113.58 to 258.81) | 6.96 (4.14 to 10.90) | 6.95 (4.14 to 10.89) |
| Estonia | 27.12 (17.76 to 40.95) | 16.89 (10.92 to 25.59) | 7.76 (4.68 to 12.22) | 7.77 (4.68 to 12.23) |
| Eswatini | 26.53 (17.22 to 39.06) | 28.75 (18.42 to 42.73) | 6.91 (4.11 to 10.83) | 6.95 (4.14 to 10.89) |
| Ethiopia | 1891.47 (1218.32 to 2852.5) | 3474.87 (2221.18 to 5292.68) | 7.85 (4.65 to 12.42) | 7.85 (4.66 to 12.41) |
| Fiji | 25.99 (16.82 to 38.87) | 25.17 (16.28 to 37.62) | 9.23 (5.51 to 14.29) | 9.22 (5.51 to 14.28) |
| Finland | 80.89 (55.46 to 115.8) | 69.94 (47.45 to 100.78) | 8.47 (5.29 to 12.9) | 8.46 (5.29 to 12.88) |
| France | 976.36 (661.24 to 1405.96) | 956.87 (644.55 to 1385.41) | 8.39 (5.23 to 12.78) | 8.39 (5.22 to 12.77) |
| Gabon | 28 (18.19 to 41.14) | 44.18 (28.21 to 65.77) | 6.91 (4.11 to 10.83) | 6.90 (4.11 to 10.81) |
| Gambia | 31.64 (20.64 to 46.23) | 68.71 (44.51 to 101.55) | 6.92 (4.12 to 10.85) | 6.92 (4.12 to 10.85) |
| Georgia | 98.37 (63.17 to 145.37) | 53.1 (33.51 to 78.98) | 7.17 (4.23 to 11.2) | 7.21 (4.23 to 11.23) |
| Germany | 1189.22 (825.18 to 1697.6) | 1095.74 (760.14 to 1569.69) | 9.20 (5.78 to 13.98) | 9.2 (5.78 to 13.98) |
| Ghana | 463.85 (301.81 to 679.59) | 892.14 (577.9 to 1320.01) | 6.96 (4.14 to 10.9) | 6.94 (4.13 to 10.87) |
| Greece | 153.13 (103.86 to 220.75) | 107.32 (73.42 to 155) | 7.78 (4.79 to 11.99) | 7.83 (4.84 to 12.11) |
| Greenland | 0.87 (0.58 to 1.28) | 0.72 (0.48 to 1.08) | 6.17 (3.71 to 9.73) | 6.15 (3.69 to 9.69) |
| Grenada | 2.21 (1.41 to 3.39) | 1.46 (0.93 to 2.26) | 6.63 (3.9 to 10.62) | 6.64 (3.91 to 10.64) |
| Guam | 3.86 (2.56 to 5.66) | 3.4 (2.22 to 5.04) | 9.27 (5.53 to 14.36) | 9.28 (5.55 to 14.38) |
| Guatemala | 266.98 (171.29 to 406.71) | 328.51 (207.88 to 508.44) | 6.61 (3.88 to 10.6) | 6.62 (3.9 to 10.61) |
| Guinea | 190.06 (125.17 to 275.69) | 417.45 (270.63 to 615.54) | 6.98 (4.15 to 10.94) | 6.94 (4.13 to 10.87) |
| Guinea-Bissau | 33.31 (21.7 to 48.76) | 62.02 (40.22 to 91.5) | 6.95 (4.14 to 10.9) | 6.93 (4.12 to 10.86) |
| Guyana | 19.65 (12.73 to 29.6) | 14.27 (9.12 to 21.87) | 6.69 (3.94 to 10.73) | 6.68 (3.93 to 10.71) |
| Haiti | 181.94 (118 to 272.93) | 291.73 (186.94 to 444.25) | 6.74 (3.97 to 10.8) | 6.71 (3.95 to 10.75) |
| Honduras | 145.77 (93.25 to 222.49) | 218.05 (138.76 to 335.88) | 6.63 (3.89 to 10.63) | 6.63 (3.90 to 10.63) |
| Hungary | 160.74 (106.22 to 235.81) | 104.02 (69.32 to 151.18) | 7.46 (4.54 to 11.48) | 7.47 (4.54 to 11.49) |
| Iceland | 5.3 (3.6 to 7.62) | 5.62 (3.81 to 8.11) | 8.41 (5.24 to 12.82) | 8.41 (5.23 to 12.81) |
| India | 25623.27 (16376.01 to 39163.75) | 29226.62 (18278.11 to 44779.53) | 7.87 (4.67 to 12.45) | 7.88 (4.68 to 12.46) |
| Indonesia | 7142.83 (4598.12 to 10741.22) | 7109.67 (4580.3 to 10687.89) | 10.51 (6.37 to 16.38) | 10.51 (6.37 to 16.39) |
| Iran (Islamic Republic of) | 1444.59 (897.68 to 2197.97) | 1165.03 (722.75 to 1791.04) | 5.72 (3.32 to 9.08) | 5.71 (3.31 to 9.07) |
| Iraq | 468.08 (298.99 to 704.26) | 724.12 (450.81 to 1126.74) | 5.70 (3.36 to 9.08) | 5.34 (3.14 to 8.56) |
| Ireland | 80.71 (54.21 to 117.42) | 82.02 (55.17 to 119.04) | 8.39 (5.22 to 12.77) | 8.38 (5.22 to 12.76) |
| Israel | 128.13 (86.88 to 184.32) | 220.62 (149.95 to 316.73) | 8.40 (5.23 to 12.79) | 8.40 (5.23 to 12.79) |
| Italy | 941.57 (606.5 to 1404.58) | 631.63 (439.31 to 877.41) | 10.27 (6.21 to 16.04) | 8.37 (5.35 to 12.43) |
| Jamaica | 55.44 (35.27 to 85.46) | 39.18 (24.72 to 60.69) | 6.62 (3.89 to 10.61) | 6.63 (3.9 to 10.62) |
| Japan | 2979.3 (1923.11 to 4427.89) | 2146.93 (1429.15 to 3152.48) | 12.87 (7.91 to 20.14) | 13.96 (8.74 to 21.49) |
| Jordan | 85.62 (53.65 to 132.93) | 194.37 (120.48 to 302.72) | 5.27 (3.09 to 8.46) | 5.26 (3.08 to 8.45) |
| Kazakhstan | 412.17 (265.78 to 627.47) | 431.48 (278.09 to 656.88) | 7.94 (4.72 to 12.60) | 7.97 (4.74 to 12.64) |
| Kenya | 866.43 (557.87 to 1311.25) | 1474.53 (927.02 to 2258.25) | 7.82 (4.64 to 12.37) | 7.84 (4.65 to 12.40) |
| Kiribati | 2.72 (1.81 to 4) | 3.87 (2.51 to 5.78) | 9.26 (5.53 to 14.36) | 9.21 (5.50 to 14.27) |
| Kuwait | 29.08 (18.2 to 45.23) | 45.12 (27.98 to 70.39) | 5.28 (3.09 to 8.47) | 5.29 (3.10 to 8.48) |
| Kyrgyzstan | 132.47 (85.75 to 199.99) | 180.73 (116.01 to 276.23) | 7.94 (4.72 to 12.59) | 7.96 (4.74 to 12.63) |
| Lao People's Democratic Republic | 171.47 (113.17 to 252.94) | 214.62 (139.95 to 318.5) | 9.34 (5.66 to 14.5) | 9.35 (5.67 to 14.51) |
| Latvia | 43.58 (28.54 to 63.16) | 22.91 (14.95 to 33.64) | 7.67 (4.57 to 11.86) | 7.68 (4.57 to 11.88) |
| Lebanon | 55.14 (34.62 to 85.38) | 68.11 (42.23 to 106.2) | 5.30 (3.11 to 8.51) | 5.28 (3.10 to 8.48) |
| Lesotho | 47.14 (30.56 to 69.7) | 43.88 (27.84 to 65.42) | 6.92 (4.12 to 10.85) | 6.93 (4.12 to 10.85) |
| Liberia | 78.27 (51.43 to 113.82) | 151.73 (97.82 to 224.8) | 6.99 (4.16 to 10.95) | 6.94 (4.13 to 10.87) |
| Libya | 98.85 (62.45 to 152.16) | 80.94 (50.33 to 126) | 5.45 (3.21 to 8.72) | 5.32 (3.12 to 8.53) |
| Lithuania | 65.8 (43.02 to 96.58) | 32.4 (20.89 to 47.82) | 7.92 (4.72 to 12.36) | 7.93 (4.73 to 12.37) |
| Luxembourg | 6.18 (4.3 to 8.56) | 9.4 (6.53 to 13.08) | 9.36 (5.87 to 14.18) | 9.37 (5.87 to 14.19) |
| Madagascar | 375.85 (244.84 to 549.79) | 812.31 (523.05 to 1205.19) | 6.94 (4.13 to 10.87) | 6.92 (4.12 to 10.85) |
| Malawi | 311.26 (204.57 to 452.33) | 563.39 (359.96 to 837.24) | 6.91 (4.11 to 10.84) | 6.91 (4.11 to 10.83) |
| Malaysia | 614.53 (401 to 912.67) | 716.56 (463.62 to 1069.54) | 9.37 (5.68 to 14.53) | 9.38 (5.69 to 14.54) |
| Maldives | 9.76 (6.46 to 14.4) | 9.42 (6.09 to 14.11) | 9.36 (5.67 to 14.51) | 9.38 (5.69 to 14.54) |
| Mali | 283.43 (186.3 to 412.03) | 796.21 (518.12 to 1166.36) | 6.94 (4.13 to 10.88) | 6.93 (4.12 to 10.86) |
| Malta | 7.28 (4.92 to 10.5) | 5.37 (3.65 to 7.72) | 8.40 (5.23 to 12.79) | 8.42 (5.24 to 12.82) |
| Marshall Islands | 2.02 (1.32 to 3.03) | 1.61 (1.04 to 2.41) | 9.24 (5.52 to 14.3) | 9.20 (5.50 to 14.25) |
| Mauritania | 63.62 (41.48 to 93) | 128.23 (82.84 to 190.01) | 6.94 (4.13 to 10.88) | 6.93 (4.12 to 10.86) |
| Mauritius | 31.01 (20.07 to 46.24) | 19.52 (12.63 to 29.18) | 9.34 (5.66 to 14.5) | 9.35 (5.67 to 14.50) |
| Mexico | 2497.97 (1567.25 to 3797.92) | 2424.75 (1494.01 to 3708.79) | 7.47 (4.40 to 11.98) | 7.48 (4.41 to 11.98) |
| Micronesia (Federated States of) | 4.26 (2.77 to 6.35) | 2.83 (1.82 to 4.24) | 9.27 (5.54 to 14.36) | 9.2 (5.49 to 14.25) |
| Monaco | 0.3 (0.2 to 0.43) | 0.41 (0.28 to 0.59) | 8.45 (5.26 to 12.88) | 8.37 (5.21 to 12.74) |
| Mongolia | 71.18 (46.04 to 107.63) | 86.02 (55.3 to 131.3) | 7.94 (4.72 to 12.59) | 7.96 (4.74 to 12.63) |
| Montenegro | 12.09 (8.04 to 17.6) | 8.36 (5.57 to 12.16) | 7.47 (4.54 to 11.49) | 7.48 (4.55 to 11.51) |
| Morocco | 519.48 (326.67 to 803.57) | 518.95 (322.97 to 807.93) | 5.32 (3.12 to 8.54) | 5.27 (3.09 to 8.46) |
| Mozambique | 426.01 (276.93 to 624.68) | 983.2 (636.82 to 1454.22) | 6.91 (4.11 to 10.84) | 6.91 (4.11 to 10.84) |
| Myanmar | 1381.47 (897.61 to 2056.84) | 1463.99 (948.71 to 2179.97) | 9.34 (5.66 to 14.49) | 9.35 (5.67 to 14.51) |
| Namibia | 41.39 (26.91 to 60.87) | 57.25 (36.61 to 85.1) | 6.91 (4.11 to 10.84) | 6.92 (4.12 to 10.84) |
| Nauru | 0.39 (0.26 to 0.57) | 0.37 (0.24 to 0.55) | 9.23 (5.52 to 14.30) | 9.24 (5.52 to 14.32) |
| Nepal | 581.39 (378.09 to 852.02) | 643.22 (411.4 to 955.34) | 6.95 (4.13 to 10.88) | 6.95 (4.13 to 10.88) |
| Netherlands | 228.27 (155.18 to 328.26) | 222.77 (150.71 to 321.44) | 8.40 (5.23 to 12.79) | 8.40 (5.23 to 12.79) |
| New Zealand | 59.6 (36.99 to 88.77) | 68.16 (43.71 to 100.44) | 7.43 (4.3 to 11.88) | 6.91 (4.15 to 10.8) |
| Nicaragua | 120.15 (76.75 to 184.05) | 131.94 (83.8 to 203.58) | 6.62 (3.89 to 10.62) | 6.64 (3.9 to 10.64) |
| Niger | 278.18 (182.67 to 404.69) | 877.02 (571.22 to 1283.66) | 6.92 (4.12 to 10.86) | 6.93 (4.12 to 10.86) |
| Nigeria | 3016.5 (1958.93 to 4535.19) | 7899.16 (5060.25 to 12015.03) | 7.80 (4.62 to 12.34) | 7.80 (4.63 to 12.35) |
| Niue | 0.07 (0.05 to 0.11) | 0.04 (0.02 to 0.05) | 9.25 (5.53 to 14.31) | 9.27 (5.54 to 14.34) |
| North Macedonia | 39.41 (26.21 to 57.38) | 24.54 (16.28 to 35.86) | 7.47 (4.54 to 11.49) | 7.47 (4.54 to 11.49) |
| Northern Mariana Islands | 1.1 (0.73 to 1.62) | 1.05 (0.67 to 1.58) | 9.09 (5.42 to 14.08) | 9.19 (5.48 to 14.24) |
| Norway | 70.17 (45.47 to 103.09) | 80.95 (51.48 to 121.8) | 8.79 (5.22 to 13.85) | 8.81 (5.24 to 13.88) |
| Oman | 43.76 (27.54 to 67.73) | 63.97 (39.8 to 100.15) | 5.28 (3.09 to 8.48) | 5.26 (3.08 to 8.44) |
| Pakistan | 3851.6 (2476.07 to 5847.24) | 6722.61 (4280.77 to 10288.93) | 7.87 (4.67 to 12.45) | 7.87 (4.67 to 12.44) |
| Palau | 0.42 (0.27 to 0.63) | 0.3 (0.19 to 0.45) | 9.24 (5.53 to 14.31) | 9.2 (5.49 to 14.26) |
| Palestine | 50.26 (31.7 to 77.59) | 98.82 (61.41 to 154.12) | 5.27 (3.09 to 8.47) | 5.26 (3.08 to 8.45) |
| Panama | 55.37 (35.29 to 85.23) | 76.88 (48.73 to 118.84) | 6.63 (3.90 to 10.63) | 6.64 (3.91 to 10.63) |
| Papua New Guinea | 159.93 (106.53 to 234.96) | 367.42 (244.95 to 539.36) | 9.42 (5.63 to 14.59) | 9.4 (5.62 to 14.56) |
| Paraguay | 110.17 (70.39 to 168.44) | 133.93 (85.02 to 206.66) | 6.63 (3.90 to 10.63) | 6.64 (3.90 to 10.63) |
| Peru | 535.86 (342.82 to 815.41) | 618.18 (394.4 to 944.46) | 6.45 (3.82 to 10.25) | 6.48 (3.84 to 10.3) |
| Philippines | 2640.89 (1727.72 to 3932.9) | 3581.77 (2297 to 5404.52) | 10.5 (6.37 to 16.37) | 10.49 (6.30 to 16.51) |
| Poland | 931.65 (604.35 to 1392.67) | 573.63 (373.71 to 852.74) | 9.67 (5.83 to 14.95) | 9.68 (5.83 to 14.95) |
| Portugal | 168 (113.81 to 246.02) | 109.92 (75.66 to 159.42) | 8.18 (5.11 to 12.64) | 8.19 (5.12 to 12.60) |
| Puerto Rico | 66.49 (42.19 to 102.51) | 30.16 (18.73 to 47.53) | 6.63 (3.9 to 10.63) | 6.64 (3.91 to 10.63) |
| Qatar | 6.49 (4.09 to 10) | 25.72 (16.11 to 39.96) | 5.27 (3.09 to 8.47) | 5.25 (3.08 to 8.43) |
| Republic of Korea | 1224.21 (818.52 to 1765.21) | 649.31 (427.43 to 946.76) | 10.84 (6.68 to 16.64) | 10.81 (6.67 to 16.57) |
| Republic of Moldova | 95.85 (62.9 to 144.77) | 40.87 (26.32 to 62.32) | 7.76 (4.68 to 12.22) | 7.77 (4.68 to 12.24) |
| Romania | 363.63 (236.32 to 534.83) | 192.26 (124.3 to 284.89) | 6.49 (3.89 to 10.13) | 6.35 (3.79 to 9.92) |
| Russian Federation | 3090.03 (1981.68 to 4744.73) | 2341.66 (1486.83 to 3636.63) | 8.89 (5.30 to 13.94) | 8.9 (5.31 to 13.96) |
| Rwanda | 232.41 (151.31 to 340.46) | 344.22 (222.04 to 509.92) | 6.91 (4.11 to 10.83) | 6.92 (4.12 to 10.85) |
| Saint Kitts and Nevis | 0.94 (0.6 to 1.45) | 0.66 (0.42 to 1.02) | 6.62 (3.89 to 10.61) | 6.62 (3.9 to 10.61) |
| Saint Lucia | 3.42 (2.18 to 5.26) | 1.99 (1.26 to 3.09) | 6.62 (3.89 to 10.61) | 6.64 (3.91 to 10.65) |
| Saint Vincent and the Grenadines | 2.74 (1.73 to 4.24) | 1.67 (1.05 to 2.6) | 6.62 (3.90 to 10.61) | 6.63 (3.91 to 10.62) |
| Samoa | 6.62 (4.33 to 9.82) | 7.35 (4.8 to 10.9) | 9.29 (5.55 to 14.4) | 9.21 (5.50 to 14.26) |
| San Marino | 0.34 (0.23 to 0.49) | 0.36 (0.24 to 0.53) | 8.39 (5.22 to 12.78) | 8.42 (5.24 to 12.81) |
| Sao Tome and Principe | 3.92 (2.54 to 5.8) | 5.42 (3.44 to 8.09) | 6.94 (4.13 to 10.87) | 6.93 (4.12 to 10.85) |
| Saudi Arabia | 346.93 (217.88 to 537.58) | 402.94 (249.85 to 628.04) | 5.33 (3.13 to 8.56) | 5.28 (3.10 to 8.48) |
| Senegal | 250.63 (163.54 to 366.16) | 441.97 (285.93 to 653.89) | 6.92 (4.12 to 10.85) | 6.95 (4.14 to 10.89) |
| Serbia | 161.63 (107.38 to 236.8) | 100.51 (66.49 to 148.09) | 7.42 (4.50 to 11.40) | 7.49 (4.54 to 11.49) |
| Seychelles | 2.22 (1.44 to 3.31) | 2.19 (1.42 to 3.27) | 9.35 (5.67 to 14.51) | 9.35 (5.67 to 14.51) |
| Sierra Leone | 126.09 (83.45 to 182.21) | 246.42 (160.05 to 362.78) | 7.02 (4.18 to 10.99) | 6.92 (4.12 to 10.84) |
| Singapore | 70.68 (47.98 to 101) | 87.3 (59.79 to 124.99) | 10.92 (6.74 to 16.78) | 10.76 (6.65 to 16.53) |
| Slovakia | 112.51 (74.72 to 166.03) | 72.73 (48.76 to 106.44) | 8.46 (5.16 to 13.01) | 8.47 (5.16 to 13.03) |
| Slovenia | 29.32 (19.2 to 43.43) | 22.1 (14.49 to 32.53) | 7.06 (4.25 to 10.89) | 7.06 (4.25 to 10.9) |
| Solomon Islands | 14.38 (9.51 to 21.18) | 23.95 (15.66 to 35.52) | 9.26 (5.53 to 14.36) | 9.22 (5.51 to 14.29) |
| Somalia | 271.17 (176.75 to 396.22) | 712.84 (464.33 to 1043.08) | 7.01 (4.17 to 10.97) | 6.96 (4.14 to 10.9) |
| South Africa | 1061.54 (678.05 to 1618.47) | 1197.62 (756.06 to 1834.27) | 7.81 (4.64 to 12.36) | 7.83 (4.65 to 12.39) |
| South Sudan | 181.99 (118.4 to 266.6) | 298.93 (194.08 to 440.24) | 6.97 (4.15 to 10.92) | 6.96 (4.14 to 10.91) |
| Spain | 583.26 (393.55 to 851.77) | 457.7 (316.58 to 650.48) | 7.68 (4.79 to 11.76) | 7.27 (4.65 to 10.91) |
| Sri Lanka | 521.06 (337.08 to 777.42) | 479.92 (310.18 to 718.77) | 9.39 (5.69 to 14.56) | 9.35 (5.67 to 14.5) |
| Sudan | 472.06 (298.75 to 725.62) | 878.66 (547.72 to 1367.14) | 5.36 (3.15 to 8.60) | 5.28 (3.10 to 8.47) |
| Suriname | 8.63 (5.49 to 13.31) | 9.58 (6.06 to 14.82) | 6.62 (3.89 to 10.60) | 6.64 (3.90 to 10.64) |
| Sweden | 166.16 (112.85 to 236.86) | 192.33 (127.43 to 284.79) | 10.73 (6.58 to 16.35) | 10.61 (6.51 to 16.62) |
| Switzerland | 78.03 (53.92 to 113.51) | 89.53 (61.39 to 130.13) | 6.77 (4.18 to 10.42) | 6.76 (4.18 to 10.41) |
| Syrian Arab Republic | 314.01 (197.1 to 486.73) | 199.06 (124.32 to 311.43) | 5.33 (3.13 to 8.55) | 5.26 (3.08 to 8.44) |
| Taiwan (Province of China) | 452.06 (299.83 to 677.38) | 273.14 (188 to 399.02) | 8.21 (4.99 to 12.76) | 9.3 (6.09 to 13.93) |
| Tajikistan | 182.65 (119.11 to 273.56) | 284.56 (183.81 to 431.01) | 7.94 (4.72 to 12.60) | 7.98 (4.75 to 12.66) |
| Thailand | 1587.41 (1026.37 to 2375.05) | 923.12 (593.9 to 1387.42) | 9.36 (5.68 to 14.51) | 9.37 (5.69 to 14.53) |
| Timor-Leste | 30.97 (20.68 to 45.34) | 48.85 (31.83 to 72.5) | 9.41 (5.70 to 14.58) | 9.38 (5.68 to 14.54) |
| Togo | 121.37 (78.89 to 178.08) | 229.47 (148.17 to 340.12) | 6.94 (4.13 to 10.87) | 6.94 (4.13 to 10.88) |
| Tokelau | 0.06 (0.04 to 0.08) | 0.04 (0.02 to 0.06) | 9.22 (5.50 to 14.26) | 9.34 (5.59 to 14.45) |
| Tonga | 3.85 (2.52 to 5.71) | 3.58 (2.34 to 5.32) | 9.22 (5.51 to 14.29) | 9.20 (5.49 to 14.27) |
| Trinidad and Tobago | 27 (17.12 to 41.77) | 18.25 (11.48 to 28.31) | 6.64 (3.91 to 10.63) | 6.64 (3.91 to 10.63) |
| Tunisia | 164.09 (102.38 to 255.46) | 146.89 (91.11 to 229.27) | 5.28 (3.09 to 8.47) | 5.28 (3.09 to 8.47) |
| Turkey | 1134.11 (719.37 to 1743.03) | 1033.8 (651.86 to 1611.78) | 5.51 (3.22 to 8.77) | 5.51 (3.22 to 8.77) |
| Turkmenistan | 118.38 (76.75 to 178.22) | 121.4 (78.11 to 185.08) | 7.94 (4.72 to 12.59) | 7.97 (4.74 to 12.65) |
| Tuvalu | 0.32 (0.22 to 0.47) | 0.34 (0.22 to 0.51) | 9.32 (5.57 to 14.46) | 9.24 (5.51 to 14.32) |
| Uganda | 575.92 (379.63 to 835.09) | 1372.89 (890.73 to 2025.53) | 6.92 (4.11 to 10.85) | 6.94 (4.13 to 10.87) |
| Ukraine | 1014.79 (650.36 to 1558.71) | 576.23 (363.95 to 896.11) | 8.89 (5.30 to 13.94) | 8.90 (5.31 to 13.97) |
| United Arab Emirates | 30.6 (19.21 to 47.55) | 70.62 (43.73 to 110.75) | 5.29 (3.10 to 8.49) | 5.26 (3.08 to 8.45) |
| United Kingdom | 1070.99 (711.48 to 1531.56) | 1201.97 (802.03 to 1720.75) | 9.80 (5.91 to 15.34) | 10.34 (6.39 to 15.95) |
| United Republic of Tanzania | 829.4 (540.63 to 1212.18) | 1684.88 (1092.39 to 2490.3) | 6.92 (4.11 to 10.84) | 6.92 (4.12 to 10.84) |
| United States of America | 3888.48 (2398.96 to 5941.05) | 3710.8 (2568.23 to 5187.73) | 6.95 (4.01 to 11.09) | 6.14 (3.86 to 9.15) |
| United States Virgin Islands | 2.12 (1.36 to 3.26) | 0.9 (0.57 to 1.4) | 6.63 (3.90 to 10.63) | 6.65 (3.91 to 10.65) |
| Uruguay | 51.49 (33.74 to 75.66) | 41.49 (26.84 to 61.91) | 6.27 (3.77 to 9.73) | 6.28 (3.78 to 9.74) |
| Uzbekistan | 674.19 (437.36 to 1014.15) | 801.09 (518.44 to 1210.31) | 7.94 (4.72 to 12.59) | 7.99 (4.75 to 12.67) |
| Vanuatu | 6.31 (4.19 to 9.27) | 10.75 (7.03 to 15.97) | 9.30 (5.56 to 14.42) | 9.25 (5.52 to 14.32) |
| Venezuela (Bolivarian Republic of) | 469.79 (300.35 to 720.23) | 441.16 (280.57 to 679.83) | 6.63 (3.89 to 10.63) | 6.63 (3.90 to 10.63) |
| Viet Nam | 2479.12 (1612.79 to 3691.51) | 2329.5 (1505.1 to 3481.12) | 9.36 (5.68 to 14.52) | 9.40 (5.70 to 14.56) |
| Yemen | 373.49 (235.4 to 576.68) | 729.28 (454.72 to 1135.03) | 5.34 (3.13 to 8.57) | 5.27 (3.09 to 8.47) |
| Zambia | 257.56 (168.15 to 375.66) | 571.52 (368.89 to 847.11) | 6.91 (4.11 to 10.83) | 6.92 (4.12 to 10.84) |
| Zimbabwe | 331.96 (215.36 to 490.11) | 435.29 (280.67 to 645.27) | 6.91 (4.11 to 10.83) | 6.92 (4.12 to 10.84) |

ASR: age-standardized rate**,** SDI: sociodemographic index, UI: uncertain interval.

*Number of cases for countries or territories is actual data not divided by 1000.

**Table S2: Number cases and ASR of prevalence for childhood myocarditis from 1990 to 2021 by SDI level, region, sex and countries/territories**

| **Characteristics** | **Number of cases (95% UI)** | | **ASR (per 100, 000) (95% UI)** | |
| --- | --- | --- | --- | --- |
|  | **1990** | **2021** | **1990** | **2021** |
| **Global** | 125.42 (96.07 to 160.71) | 135.27 (102.35 to 173.82) | 7.17 (5.26 to 9.70) | 6.81 (4.90 to 9.32) |
| **Age (years)** |  |  |  |  |
| <5 | 63.75 (48.81 to 83.84) | 58.67 (44.33 to 78.15) |  |  |
| 5-9 | 39.53 (27.87 to 54.99) | 47.46 (32.65 to 66.45) |  |  |
| 10-14 | 22.13 (15.35 to 30.63) | 29.14 (20.1 to 40.55) |  |  |
| **Sex** |  |  |  |  |
| Male | 63.6 (49.19 to 80.15) | 69.49 (52.8 to 88.53) | 7.08 (5.20 to 9.54) | 6.77 (4.93 to 9.23) |
| Female | 61.81 (47.48 to 80.45) | 65.77 (49.07 to 87.53) | 7.28 (5.26 to 9.90) | 6.86 (4.89 to 9.50) |
| **SDI level** |  |  |  |  |
| High | 27.76 (21.58 to 35.87) | 36.36 (27 to 47.77) | 15.16 (11.05 to 20.60) | 21.72 (15.45 to 29.88) |
| High-middle | 20.6 (15.77 to 26.22) | 18.65 (13.96 to 24.89) | 7.59 (5.54 to 10.34) | 8.24 (5.89 to 11.38) |
| Middle | 36.98 (28.34 to 47.04) | 36.01 (26.81 to 46.45) | 6.42 (4.68 to 8.74) | 6.47 (4.62 to 8.93) |
| Low-middle | 27.08 (20.83 to 34.95) | 26.82 (20.32 to 34.48) | 5.63 (4.06 to 7.65) | 4.69 (3.35 to 6.44) |
| Low | 12.88 (9.68 to 17.1) | 17.33 (12.84 to 22.53) | 5.34 (3.69 to 7.47) | 3.74 (2.58 to 5.23) |
| **Region** |  |  |  |  |
| Andean Latin America | 0.47 (0.36 to 0.6) | 0.42 (0.3 to 0.56) | 3.13 (2.19 to 4.29) | 2.31 (1.56 to 3.24) |
| Australasia | 1.03 (0.78 to 1.35) | 1.34 (0.98 to 1.78) | 22.87 (16.07 to 32.36) | 23.99 (16.72 to 33.97) |
| Caribbean | 0.63 (0.48 to 0.81) | 0.73 (0.55 to 0.94) | 5.47 (3.88 to 7.54) | 6.42 (4.5 to 8.99) |
| Central Asia | 1.4 (1.08 to 1.78) | 1.48 (1.15 to 1.87) | 5.53 (3.97 to 7.57) | 5.33 (3.84 to 7.21) |
| Central Europe | 3.1 (2.35 to 3.98) | 2.38 (1.76 to 3.15) | 10.79 (7.82 to 14.82) | 13.74 (9.73 to 19.22) |
| Central Latin America | 2.19 (1.7 to 2.8) | 2.81 (2.17 to 3.63) | 3.40 (2.42 to 4.63) | 4.50 (3.24 to 6.17) |
| Central Sub-Saharan Africa | 0.94 (0.66 to 1.28) | 1.46 (1.05 to 1.97) | 3.53 (2.29 to 5.17) | 2.48 (1.63 to 3.57) |
| East Asia | 27.61 (20.98 to 35.58) | 27.58 (20.19 to 37.52) | 8.39 (5.98 to 11.58) | 10.55 (7.34 to 14.96) |
| Eastern Europe | 5.16 (3.96 to 6.55) | 2.51 (1.85 to 3.23) | 10.06 (7.24 to 13.65) | 7.16 (5.16 to 9.82) |
| Eastern Sub-Saharan Africa | 5.57 (4.04 to 7.54) | 5.3 (3.87 to 7.03) | 5.79 (3.94 to 8.29) | 2.96 (2.02 to 4.16) |
| High-income Asia Pacific | 4.12 (3.12 to 5.27) | 6.01 (4.46 to 8.06) | 12.04 (8.63 to 16.43) | 27.46 (19.38 to 38.42) |
| High-income North America | 15.94 (12.06 to 21.08) | 20.45 (14.94 to 27.33) | 25.87 (18.57 to 35.64) | 32.44 (22.5 to 45.16) |
| North Africa and Middle East | 6.65 (5.18 to 8.43) | 7.2 (5.59 to 9.25) | 4.66 (3.39 to 6.35) | 3.97 (2.85 to 5.35) |
| Oceania | 0.08 (0.06 to 0.11) | 0.14 (0.1 to 0.2) | 2.90 (1.94 to 4.13) | 2.84 (1.89 to 4.09) |
| South Asia | 26 (19.9 to 33.61) | 24.77 (18.58 to 32.1) | 5.90 (4.22 to 8.11) | 5.03 (3.57 to 6.94) |
| South-East Asia Region | 9.99 (7.6 to 12.72) | 9.58 (7.26 to 12.37) | 5.88 (4.28 to 7.87) | 5.60 (3.99 to 7.61) |
| Southern Latin America | 0.74 (0.57 to 0.93) | 0.54 (0.42 to 0.69) | 4.97 (3.61 to 6.77) | 3.87 (2.83 to 5.21) |
| Southern Sub-Saharan Africa | 0.9 (0.64 to 1.19) | 0.83 (0.6 to 1.12) | 4.29 (2.92 to 6.12) | 3.45 (2.29 to 4.91) |
| Tropical Latin America | 2.62 (2.01 to 3.29) | 2.93 (2.24 to 3.72) | 4.96 (3.63 to 6.72) | 5.87 (4.24 to 7.98) |
| Western Europe | 5.49 (4.3 to 6.88) | 7.34 (5.6 to 9.45) | 7.89 (5.91 to 10.42) | 11.09 (8.13 to 15.12) |
| Western Sub-Saharan Africa | 4.81 (3.42 to 6.5) | 9.47 (6.72 to 12.57) | 5.15 (3.35 to 7.52) | 4.31 (2.82 to 6.23) |
| **Countries or territories*** |  |  |  |  |
| Afghanistan | 83.54 (61.72 to 112.6) | 259.68 (184.55 to 355.2) | 1.91 (1.29 to 2.72) | 1.83 (1.23 to 2.61) |
| Albania | 114.02 (85.08 to 148.93) | 51.81 (38.12 to 69.56) | 10.13 (7.04 to 14.51) | 11.9 (8.3 to 16.97) |
| Algeria | 254.73 (194.63 to 328.7) | 319.65 (241.49 to 414.28) | 2.38 (1.66 to 3.27) | 2.40 (1.68 to 3.32) |
| American Samoa | 0.44 (0.3 to 0.62) | 0.34 (0.22 to 0.5) | 2.31 (1.42 to 3.56) | 2.38 (1.48 to 3.61) |
| Andorra | 1.43 (1.1 to 1.85) | 1.23 (0.94 to 1.59) | 16.31 (11.68 to 22.71) | 13.37 (9.5 to 18.45) |
| Angola | 201.09 (138.75 to 279.11) | 372.65 (265.44 to 503.97) | 4.00 (2.56 to 5.85) | 2.43 (1.60 to 3.49) |
| Antigua and Barbuda | 2.26 (1.66 to 3.01) | 1.91 (1.39 to 2.56) | 12.52 (8.57 to 17.64) | 11.68 (7.97 to 16.82) |
| Argentina | 482.89 (369.14 to 612.85) | 349.77 (264.58 to 445.06) | 4.82 (3.44 to 6.58) | 3.53 (2.55 to 4.77) |
| Armenia | 52.29 (40.61 to 66.57) | 18.78 (13.74 to 25.62) | 4.99 (3.59 to 6.64) | 3.16 (2.16 to 4.44) |
| Australia | 870.34 (661.08 to 1149.98) | 1021.01 (749.74 to 1371.36) | 23.34 (16.27 to 33.1) | 22.16 (15.28 to 31.63) |
| Austria | 40.21 (31.21 to 51.24) | 103.17 (81.22 to 129.52) | 3.01 (2.16 to 4.09) | 8.06 (5.93 to 10.85) |
| Azerbaijan | 158.88 (122.83 to 202.82) | 158.4 (119.64 to 203.42) | 6.47 (4.64 to 8.84) | 6.77 (4.79 to 9.33) |
| Bahamas | 3.84 (2.97 to 5.01) | 3.8 (2.84 to 4.96) | 4.80 (3.42 to 6.59) | 5.00 (3.53 to 6.91) |
| Bahrain | 4.01 (3.08 to 5.16) | 7.37 (5.6 to 9.61) | 2.42 (1.72 to 3.32) | 2.52 (1.78 to 3.45) |
| Bangladesh | 2339.06 (1790.45 to 3048.26) | 2054.41 (1577.62 to 2706.05) | 4.62 (3.23 to 6.40) | 4.60 (3.18 to 6.32) |
| Barbados | 3.54 (2.67 to 4.62) | 3.7 (2.76 to 4.93) | 5.78 (4.09 to 8.06) | 8.25 (5.71 to 11.82) |
| Belarus | 181.73 (136.95 to 234.99) | 81.06 (60.44 to 106.07) | 7.56 (5.40 to 10.36) | 5.20 (3.68 to 7.07) |
| Belgium | 52.38 (40.16 to 65.98) | 158.91 (119.52 to 213.05) | 2.94 (2.09 to 3.96) | 8.52 (5.97 to 12.10) |
| Belize | 4.6 (3.54 to 5.86) | 5.53 (4.22 to 7.12) | 5.57 (4.00 to 7.67) | 4.61 (3.25 to 6.34) |
| Benin | 99.36 (67.28 to 139.35) | 212.83 (148.81 to 292.59) | 3.88 (2.42 to 5.88) | 3.40 (2.20 to 5.06) |
| Bermuda | 0.72 (0.55 to 0.92) | 0.76 (0.57 to 1) | 6.06 (4.27 to 8.33) | 9.49 (6.72 to 13.32) |
| Bhutan | 14.26 (11 to 18.46) | 8.67 (6.68 to 11.05) | 5.36 (3.81 to 7.40) | 4.73 (3.31 to 6.42) |
| Bolivia (Plurinational State of) | 72.14 (55.07 to 94.45) | 76.73 (54.3 to 104.92) | 2.65 (1.84 to 3.69) | 2.20 (1.46 to 3.14) |
| Bosnia and Herzegovina | 45.48 (35.32 to 57.91) | 20.35 (15.82 to 25.88) | 4.21 (3.09 to 5.60) | 4.23 (3.11 to 5.61) |
| Botswana | 20.56 (14.89 to 27.59) | 22.88 (16.42 to 30.44) | 3.46 (2.31 to 5.04) | 3.30 (2.19 to 4.73) |
| Brazil | 2552.47 (1960.77 to 3210) | 2863.92 (2188.35 to 3631.68) | 4.99 (3.66 to 6.78) | 5.97 (4.30 to 8.12) |
| Brunei Darussalam | 19.06 (14.39 to 25.68) | 20.16 (14.85 to 27.21) | 20.64 (14.17 to 29.70) | 21.65 (14.77 to 30.95) |
| Bulgaria | 100.55 (76.89 to 129.42) | 110.33 (80.07 to 148.4) | 5.91 (4.20 to 8.12) | 11.53 (7.88 to 16.49) |
| Burkina Faso | 198.47 (135.22 to 277.62) | 384.69 (263.09 to 523.64) | 4.02 (2.52 to 6.07) | 3.57 (2.24 to 5.31) |
| Burundi | 136.21 (94.21 to 193.95) | 156.76 (113.88 to 210.42) | 4.83 (3.12 to 7.27) | 2.66 (1.77 to 3.79) |
| Cabo Verde | 10.84 (7.14 to 16.01) | 6.42 (4.37 to 9) | 6.67 (4.09 to 10.47) | 4.66 (2.93 to 7.02) |
| Cambodia | 163.48 (122.36 to 212.87) | 196.42 (147.43 to 257.95) | 3.47 (2.41 to 4.83) | 3.85 (2.69 to 5.27) |
| Cameroon | 198.66 (136.88 to 276.33) | 440.84 (306.61 to 599.83) | 3.85 (2.47 to 5.83) | 3.25 (2.10 to 4.76) |
| Canada | 971.87 (715.7 to 1295.79) | 1971.43 (1430.14 to 2663.82) | 17.11 (11.73 to 24.91) | 32.86 (22.12 to 47.99) |
| Central African Republic | 42.41 (30.08 to 57.47) | 56.69 (40.64 to 76.1) | 3.31 (2.17 to 4.84) | 2.47 (1.63 to 3.52) |
| Chad | 116.98 (81.43 to 163.99) | 329.88 (229.55 to 451.79) | 3.75 (2.37 to 5.66) | 3.50 (2.19 to 5.21) |
| Chile | 196.61 (153.63 to 246.99) | 155.79 (121.58 to 198.69) | 4.91 (3.55 to 6.62) | 4.44 (3.24 to 5.97) |
| China | 26988.71 (20475.58 to 34758.65) | 26857.42 (19638.37 to 36541.96) | 8.49 (6.06 to 11.73) | 10.58 (7.35 to 15.04) |
| Colombia | 422.7 (321.16 to 545.36) | 709.49 (533.12 to 936.08) | 3.61 (2.55 to 4.91) | 6.82 (4.77 to 9.70) |
| Comoros | 9.1 (6.48 to 12.36) | 6.29 (4.5 to 8.42) | 4.13 (2.70 to 6.06) | 2.63 (1.73 to 3.73) |
| Congo | 33.61 (24.06 to 45.27) | 45.13 (31.59 to 61.91) | 3.14 (2.07 to 4.56) | 2.35 (1.52 to 3.36) |
| Cook Islands | 0.15 (0.1 to 0.22) | 0.09 (0.05 to 0.13) | 2.25 (1.35 to 3.49) | 2.24 (1.34 to 3.48) |
| Costa Rica | 80.11 (62.2 to 101.56) | 83.03 (62.96 to 110.47) | 7.08 (5.02 to 9.67) | 8.39 (5.96 to 11.88) |
| Cote d’Ivoire | 222.37 (151.16 to 305.25) | 398.51 (278.61 to 541.75) | 3.71 (2.36 to 5.67) | 3.37 (2.18 to 5.05) |
| Croatia | 113.92 (84.62 to 148.12) | 133.8 (95.99 to 180.77) | 11.80 (8.20 to 16.52) | 22.89 (15.4 to 32.97) |
| Cuba | 127.99 (98.74 to 163.29) | 124.6 (92.88 to 161.9) | 5.15 (3.62 to 7.02) | 7.25 (5.06 to 10.16) |
| Cyprus | 15.01 (11.48 to 18.99) | 13.52 (10.36 to 17.57) | 7.70 (5.54 to 10.45) | 6.22 (4.45 to 8.45) |
| Czechia | 128.03 (95.49 to 166.02) | 201.56 (148.66 to 271.03) | 6.01 (4.29 to 8.21) | 11.96 (8.23 to 16.95) |
| Democratic People's Republic of Korea | 387.93 (297.75 to 503.56) | 307.48 (229.56 to 403.21) | 6.34 (4.52 to 8.64) | 6.61 (4.66 to 9.18) |
| Democratic Republic of the Congo | 643.37 (452.21 to 869.29) | 958.74 (688.11 to 1291.78) | 3.45 (2.22 to 5.09) | 2.52 (1.65 to 3.62) |
| Denmark | 36.26 (28.28 to 45.38) | 96.32 (72.48 to 128.39) | 4.21 (3.10 to 5.57) | 10.36 (7.26 to 14.59) |
| Djibouti | 7.7 (5.46 to 10.61) | 10.73 (7.7 to 14.36) | 4.33 (2.85 to 6.38) | 2.60 (1.74 to 3.70) |
| Dominica | 2.07 (1.59 to 2.71) | 0.98 (0.73 to 1.28) | 8.42 (5.95 to 11.87) | 7.61 (5.39 to 10.41) |
| Dominican Republic | 89.01 (68.1 to 114.83) | 101.3 (76.63 to 130.53) | 3.28 (2.28 to 4.47) | 3.45 (2.41 to 4.75) |
| Ecuador | 142.54 (111.19 to 181.68) | 117.95 (85.93 to 159.44) | 3.69 (2.64 to 5.00) | 2.34 (1.59 to 3.29) |
| Egypt | 2856.96 (2141.18 to 3762.01) | 2645.17 (1968.49 to 3462.3) | 12.38 (8.64 to 17.57) | 7.13 (5.00 to 9.89) |
| El Salvador | 56.76 (42.72 to 75.11) | 45.79 (33.51 to 61.89) | 2.63 (1.80 to 3.66) | 2.52 (1.71 to 3.51) |
| Equatorial Guinea | 7.22 (5.1 to 9.77) | 13.63 (9.53 to 18.77) | 3.47 (2.30 to 5.07) | 2.34 (1.53 to 3.36) |
| Eritrea | 86.13 (59.74 to 120.09) | 68.09 (49.26 to 91.17) | 5.14 (3.32 to 7.63) | 2.68 (1.77 to 3.81) |
| Estonia | 14.17 (10.91 to 18.01) | 5.64 (4.08 to 7.72) | 4.07 (2.97 to 5.43) | 2.62 (1.80 to 3.69) |
| Eswatini | 15.23 (10.76 to 20.85) | 12.06 (8.72 to 16.07) | 3.87 (2.53 to 5.72) | 2.93 (1.93 to 4.21) |
| Ethiopia | 2464.81 (1777.83 to 3285.78) | 1783.75 (1353.47 to 2301.25) | 9.46 (6.50 to 13.24) | 4.00 (2.85 to 5.49) |
| Fiji | 8.01 (5.71 to 10.8) | 8.33 (6.03 to 11.13) | 2.85 (1.89 to 4.09) | 3.06 (2.07 to 4.35) |
| Finland | 96.63 (75.25 to 123.77) | 184.55 (138.44 to 248.75) | 10.26 (7.44 to 13.95) | 22.91 (15.84 to 32.74) |
| France | 321.34 (236.92 to 426.27) | 1028.51 (799.8 to 1316.74) | 2.77 (1.94 to 3.85) | 9.30 (6.75 to 12.7) |
| Gabon | 13.31 (9.58 to 17.61) | 15.45 (10.89 to 21.02) | 3.20 (2.10 to 4.64) | 2.42 (1.60 to 3.47) |
| Gambia | 20.15 (13.86 to 28.09) | 33.27 (23.12 to 44.81) | 4.14 (2.59 to 6.26) | 3.33 (2.13 to 4.90) |
| Georgia | 114.84 (86.85 to 148.66) | 40.01 (30.69 to 52.29) | 8.43 (5.98 to 11.8) | 5.42 (3.87 to 7.40) |
| Germany | 624.83 (491.92 to 776.55) | 1280.34 (977.25 to 1659.03) | 4.85 (3.56 to 6.41) | 10.8 (7.73 to 14.73) |
| Ghana | 228.93 (163.28 to 301.14) | 322.76 (226.87 to 434.12) | 3.30 (2.19 to 4.81) | 2.49 (1.59 to 3.66) |
| Greece | 45.13 (31.75 to 61.24) | 75.02 (57.78 to 95.12) | 2.30 (1.55 to 3.3) | 5.60 (4.04 to 7.56) |
| Greenland | 1.72 (1.3 to 2.23) | 2.1 (1.6 to 2.79) | 11.45 (8.16 to 15.89) | 18.04 (12.62 to 25.6) |
| Grenada | 2.27 (1.71 to 2.97) | 1.49 (1.14 to 1.97) | 6.75 (4.75 to 9.40) | 7.04 (4.87 to 9.90) |
| Guam | 1.95 (1.51 to 2.46) | 1.61 (1.24 to 2.07) | 4.63 (3.32 to 6.24) | 4.34 (3.06 to 5.99) |
| Guatemala | 221.31 (170.12 to 284.57) | 307.95 (226.52 to 408.27) | 5.3 (3.78 to 7.33) | 6.43 (4.41 to 9.18) |
| Guinea | 114.02 (78.1 to 158.39) | 202.05 (141.06 to 277.05) | 3.88 (2.44 to 5.92) | 3.28 (2.11 to 4.87) |
| Guinea-Bissau | 19.44 (13.47 to 26.94) | 28.13 (19.68 to 38) | 3.87 (2.48 to 5.86) | 3.09 (1.99 to 4.55) |
| Guyana | 45.84 (33.54 to 60.26) | 40.48 (29.49 to 54.49) | 15.43 (10.51 to 22.47) | 19.01 (12.76 to 27.98) |
| Haiti | 196.81 (145.86 to 259.86) | 316.19 (231.09 to 411.95) | 6.94 (4.82 to 9.91) | 7.20 (5.03 to 10.23) |
| Honduras | 73.41 (56.71 to 94.36) | 98.36 (74.24 to 130.1) | 3.29 (2.32 to 4.53) | 3.02 (2.10 to 4.14) |
| Hungary | 90.53 (69.75 to 114.21) | 98.67 (73.72 to 130.73) | 4.42 (3.22 to 5.89) | 7.19 (5.08 to 9.92) |
| Iceland | 2.24 (1.72 to 2.86) | 5.82 (4.4 to 7.65) | 3.55 (2.57 to 4.81) | 8.8 (6.18 to 12.34) |
| India | 19633.92 (14982.74 to 25198.6) | 17322.36 (12897.43 to 22488.85) | 5.95 (4.24 to 8.19) | 4.88 (3.44 to 6.75) |
| Indonesia | 3838.42 (2873.26 to 4941) | 3714.9 (2726.49 to 4788.74) | 5.74 (4.12 to 7.87) | 5.58 (3.96 to 7.73) |
| Iran (Islamic Republic of) | 608.42 (449.59 to 796.28) | 446.98 (314.65 to 593.13) | 2.4 0(1.67 to 3.34) | 2.24 (1.52 to 3.10) |
| Iraq | 754.72 (575.71 to 986.39) | 1151.58 (870.61 to 1487.37) | 8.88 (6.28 to 12.53) | 8.84 (6.27 to 12.12) |
| Ireland | 39.77 (31.48 to 50.57) | 189.84 (138.7 to 253.8) | 4.19 (3.08 to 5.51) | 19.72 (13.6 to 28.21) |
| Israel | 52.96 (41.19 to 67.13) | 171.5 (131.07 to 223.06) | 3.48 (2.53 to 4.67) | 6.52 (4.64 to 9.00) |
| Italy | 1096.55 (832.05 to 1423.97) | 515.23 (384.13 to 670.73) | 12.14 (8.75 to 16.28) | 6.85 (4.91 to 9.37) |
| Jamaica | 38.41 (29.48 to 49.03) | 35.79 (27.17 to 46.78) | 4.63 (3.31 to 6.34) | 6.36 (4.50 to 8.86) |
| Japan | 2125.11 (1647.85 to 2709.72) | 4320.39 (3168.26 to 5781.78) | 9.50 (7.01 to 12.75) | 28.63 (19.88 to 39.9) |
| Jordan | 42.8 (32.92 to 55.49) | 100.37 (75.02 to 128.79) | 2.60 (1.83 to 3.54) | 2.83 (1.98 to 3.86) |
| Kazakhstan | 183.29 (137.16 to 240.33) | 427.11 (327.9 to 559.29) | 3.53 (2.48 to 4.90) | 7.85 (5.50 to 11.00) |
| Kenya | 391.33 (277.06 to 524.85) | 469.84 (322.04 to 652.21) | 3.43 (2.27 to 4.95) | 2.52 (1.63 to 3.63) |
| Kiribati | 1.06 (0.8 to 1.37) | 1.6 (1.22 to 2.08) | 3.61 (2.50 to 4.99) | 3.81 (2.68 to 5.33) |
| Kuwait | 36.31 (27.67 to 48.5) | 102.6 (74.39 to 137.43) | 6.43 (4.58 to 8.97) | 12.55 (8.68 to 17.74) |
| Kyrgyzstan | 137.14 (101.78 to 179.75) | 218.84 (162.93 to 291.48) | 8.02 (5.61 to 11.28) | 9.58 (6.71 to 13.52) |
| Lao People's Democratic Republic | 71.86 (54.29 to 92.59) | 91.05 (67.12 to 119.31) | 3.85 (2.68 to 5.32) | 3.96 (2.76 to 5.49) |
| Latvia | 18.01 (13.47 to 23.29) | 7.32 (5.23 to 9.9) | 3.16 (2.23 to 4.29) | 2.46 (1.65 to 3.47) |
| Lebanon | 59.08 (45.74 to 74.95) | 79.11 (61.26 to 102.6) | 5.55 (4.01 to 7.55) | 6.34 (4.56 to 8.73) |
| Lesotho | 22.79 (16.53 to 30.59) | 18.18 (12.86 to 24.48) | 3.32 (2.22 to 4.82) | 2.90 (1.92 to 4.15) |
| Liberia | 47.44 (32.78 to 66.87) | 70.92 (49.02 to 95.9) | 3.96 (2.51 to 6.00) | 3.24 (2.08 to 4.84) |
| Libya | 66.22 (52.14 to 83.34) | 52.1 (39.98 to 67.13) | 3.65 (2.66 to 4.95) | 3.76 (2.68 to 5.16) |
| Lithuania | 26.48 (19.74 to 34.11) | 10.38 (7.38 to 14.14) | 3.19 (2.24 to 4.28) | 2.54 (1.71 to 3.62) |
| Luxembourg | 2.9 (2.3 to 3.63) | 15.14 (11.34 to 20.3) | 4.40 (3.24 to 5.84) | 15.22 (10.69 to 21.85) |
| Madagascar | 277.57 (194.69 to 387.03) | 327.47 (232.74 to 444.29) | 4.85 (3.14 to 7.27) | 2.79 (1.83 to 4.00) |
| Malawi | 255.87 (175.17 to 368.02) | 209.16 (150.36 to 285.23) | 5.16 (3.29 to 7.96) | 2.59 (1.72 to 3.75) |
| Malaysia | 515.96 (396.44 to 649.14) | 536.77 (407.15 to 703.97) | 7.77 (5.6 to 10.54) | 7.16 (5.11 to 9.94) |
| Maldives | 6.69 (5.16 to 8.57) | 5.57 (4.22 to 7.28) | 6.16 (4.39 to 8.39) | 5.64 (4.01 to 7.74) |
| Mali | 171.04 (115.76 to 238.67) | 408.79 (282.45 to 556.28) | 3.89 (2.43 to 5.97) | 3.41 (2.19 to 5.09) |
| Malta | 6.59 (5.2 to 8.17) | 17.3 (12.76 to 23.71) | 7.69 (5.66 to 10.31) | 27.17 (18.16 to 39.4) |
| Marshall Islands | 0.6 (0.42 to 0.83) | 0.48 (0.34 to 0.65) | 2.75 (1.82 to 3.99) | 2.73 (1.8 to 3.97) |
| Mauritania | 37.15 (25.73 to 51.17) | 60.28 (42.55 to 82.17) | 3.83 (2.38 to 5.79) | 3.24 (2.10 to 4.82) |
| Mauritius | 8.4 (5.65 to 11.8) | 8.39 (6.15 to 10.95) | 2.54 (1.62 to 3.76) | 4.10 (2.90 to 5.55) |
| Mexico | 951.7 (694.82 to 1275.95) | 1181.16 (869.97 to 1531.47) | 2.85 (1.96 to 3.96) | 3.72 (2.62 to 5.11) |
| Micronesia (Federated States of) | 1.28 (0.9 to 1.74) | 0.84 (0.59 to 1.16) | 2.79 (1.85 to 4.03) | 2.74 (1.82 to 3.96) |
| Monaco | 0.24 (0.19 to 0.31) | 0.25 (0.2 to 0.32) | 7.05 (5.22 to 9.39) | 5.13 (3.81 to 6.8) |
| Mongolia | 79.33 (60.32 to 102.57) | 68.66 (51.32 to 89.95) | 8.68 (6.15 to 12.18) | 6.29 (4.38 to 8.8) |
| Montenegro | 20.31 (15.58 to 26.29) | 9.41 (7.23 to 11.98) | 12.86 (9.27 to 17.81) | 8.63 (6.17 to 11.6) |
| Morocco | 339.53 (265.37 to 436.2) | 311.53 (239.98 to 405.57) | 3.42 (2.47 to 4.64) | 3.22 (2.25 to 4.43) |
| Mozambique | 263.74 (185.55 to 365.59) | 325.07 (225.57 to 444.9) | 4.07 (2.65 to 6.00) | 2.27 (1.46 to 3.28) |
| Myanmar | 599.67 (452.57 to 775.56) | 687.25 (504.54 to 909.33) | 4.07 (2.86 to 5.6) | 4.42 (3.13 to 6.12) |
| Namibia | 22.81 (16.15 to 31.13) | 25.02 (18.35 to 33.8) | 3.72 (2.44 to 5.43) | 3.05 (2.01 to 4.38) |
| Nauru | 0.12 (0.09 to 0.16) | 0.11 (0.08 to 0.15) | 2.83 (1.88 to 4.05) | 2.80 (1.85 to 4.04) |
| Nepal | 588.53 (441.61 to 773.29) | 491.32 (376.3 to 640.71) | 6.61 (4.68 to 9.13) | 5.41 (3.81 to 7.52) |
| Netherlands | 88.99 (68.83 to 114.61) | 244.6 (188.82 to 319.26) | 3.29 (2.38 to 4.48) | 9.36 (6.73 to 12.92) |
| New Zealand | 163.72 (120.9 to 213.57) | 314.12 (222.64 to 431.01) | 20.71 (14.59 to 28.98) | 32.84 (22.06 to 47.08) |
| Nicaragua | 64.89 (50.46 to 82.9) | 62.14 (46.72 to 81.98) | 3.53 (2.51 to 4.83) | 3.16 (2.22 to 4.32) |
| Niger | 175.91 (119.84 to 248.93) | 456.15 (318.67 to 626.86) | 4.07 (2.55 to 6.25) | 3.44 (2.22 to 5.16) |
| Nigeria | 2897.09 (2006.29 to 3988.07) | 5729.04 (4057.15 to 7643.52) | 6.91 (4.51 to 10.07) | 5.54 (3.62 to 7.95) |
| Niue | 0.02 (0.02 to 0.03) | 0.01 (0.01 to 0.02) | 2.89 (1.94 to 4.13) | 2.93 (1.97 to 4.18) |
| North Macedonia | 28.61 (22.19 to 35.79) | 17.32 (13.43 to 22.12) | 5.50 (4.06 to 7.38) | 5.33 (3.87 to 7.2) |
| Northern Mariana Islands | 0.4 (0.3 to 0.52) | 0.46 (0.35 to 0.61) | 3.26 (2.25 to 4.55) | 4.17 (2.90 to 5.76) |
| Norway | 91.83 (72.36 to 117.76) | 112.31 (84.57 to 147.6) | 11.64 (8.58 to 15.64) | 12.54 (8.90 to 17.17) |
| Oman | 55.28 (42.44 to 72.54) | 67.99 (52.19 to 88.64) | 6.29 (4.57 to 8.61) | 5.53 (3.97 to 7.65) |
| Pakistan | 3422.06 (2587.26 to 4431.28) | 4892.63 (3637.84 to 6451.13) | 6.72 (4.70 to 9.24) | 5.73 (4.01 to 8.07) |
| Palau | 0.18 (0.13 to 0.23) | 0.12 (0.09 to 0.16) | 3.86 (2.69 to 5.35) | 3.7 (2.55 to 5.14) |
| Palestine | 32.22 (25.03 to 40.84) | 60.47 (47.1 to 78.25) | 3.19 (2.28 to 4.33) | 3.28 (2.35 to 4.50) |
| Panama | 22.95 (17.45 to 30.23) | 46.24 (34.67 to 60.94) | 2.76 (1.93 to 3.76) | 4.04 (2.85 to 5.59) |
| Papua New Guinea | 49.28 (35.83 to 66.44) | 110.58 (79.53 to 149.98) | 2.89 (1.92 to 4.16) | 2.82 (1.87 to 4.06) |
| Paraguay | 66.6 (50.92 to 85.81) | 67.48 (50.33 to 88.91) | 3.94 (2.80 to 5.35) | 3.38 (2.35 to 4.70) |
| Peru | 251.67 (189.25 to 328.37) | 222.52 (161.77 to 299.89) | 3.03 (2.09 to 4.21) | 2.34 (1.57 to 3.28) |
| Philippines | 1167.01 (872.09 to 1515.93) | 1442.73 (1062.62 to 1911.58) | 4.61 (3.27 to 6.30) | 4.26 (2.97 to 5.91) |
| Poland | 1316.62 (999.98 to 1707.7) | 814.71 (600.55 to 1079.33) | 14.06 (10.09 to 18.88) | 14.07 (9.82 to 19.51) |
| Portugal | 53.8 (39.24 to 72.65) | 83.68 (62.55 to 108.77) | 2.64 (1.83 to 3.70) | 6.35 (4.53 to 8.75) |
| Puerto Rico | 27.44 (20.32 to 36.24) | 23.43 (17.1 to 31.22) | 2.78 (1.95 to 3.81) | 5.63 (3.88 to 8.03) |
| Qatar | 5.27 (4.1 to 6.67) | 23.64 (17.96 to 30.6) | 4.07 (2.94 to 5.47) | 4.70 (3.34 to 6.43) |
| Republic of Korea | 1750.78 (1293.08 to 2356.63) | 1208.5 (866.94 to 1623.52) | 15.69 (10.83 to 22.61) | 20.54 (14.14 to 29.34) |
| Republic of Moldova | 65.44 (50.79 to 82.96) | 20.15 (15.21 to 26.15) | 5.29 (3.79 to 7.13) | 3.87 (2.73 to 5.17) |
| Romania | 895 (659.44 to 1212.35) | 773.95 (561.26 to 1067.48) | 16.59 (11.26 to 24.43) | 26.33 (17.79 to 38.33) |
| Russian Federation | 4056.63 (3104.48 to 5243.65) | 1918 (1425.97 to 2471.1) | 11.73 (8.43 to 15.95) | 7.42 (5.33 to 10.2) |
| Rwanda | 169.96 (116.77 to 238.27) | 128.78 (92.5 to 174.24) | 4.73 (3.07 to 7.00) | 2.59 (1.72 to 3.70) |
| Saint Kitts and Nevis | 0.39 (0.29 to 0.51) | 0.3 (0.22 to 0.39) | 2.76 (1.91 to 3.78) | 3.03 (2.13 to 4.17) |
| Saint Lucia | 5.69 (4.24 to 7.59) | 3.56 (2.69 to 4.76) | 11.11 (7.70 to 15.79) | 12.55 (8.72 to 17.76) |
| Saint Vincent and the Grenadines | 1.92 (1.43 to 2.5) | 2.08 (1.54 to 2.77) | 4.76 (3.31 to 6.58) | 8.73 (6.05 to 12.37) |
| Samoa | 2.08 (1.49 to 2.79) | 2.25 (1.61 to 3.03) | 2.91 (1.95 to 4.14) | 2.82 (1.88 to 4.06) |
| San Marino | 0.47 (0.37 to 0.6) | 0.62 (0.47 to 0.82) | 12.65 (9.22 to 17.16) | 15.34 (10.87 to 21.58) |
| Sao Tome and Principe | 2.28 (1.57 to 3.2) | 2.57 (1.76 to 3.46) | 3.98 (2.51 to 6.01) | 3.38 (2.16 to 5.08) |
| Saudi Arabia | 309.62 (241.2 to 398.4) | 254.82 (193.76 to 327.06) | 4.63 (3.30 to 6.27) | 3.43 (2.43 to 4.64) |
| Senegal | 108.45 (76.02 to 145.98) | 154.91 (108.73 to 207.79) | 2.87 (1.84 to 4.32) | 2.43 (1.56 to 3.52) |
| Serbia | 112.71 (88.22 to 141.41) | 53.41 (41.43 to 67.4) | 5.29 (3.84 to 7.08) | 4.12 (3.02 to 5.46) |
| Seychelles | 1.95 (1.51 to 2.49) | 1.59 (1.22 to 2.03) | 8.30 (5.98 to 11.27) | 6.88 (4.94 to 9.40) |
| Sierra Leone | 73.99 (50.62 to 103.78) | 116.96 (82.69 to 157.96) | 3.81 (2.41 to 5.78) | 3.21 (2.07 to 4.76) |
| Singapore | 222.76 (160.14 to 301.27) | 459.58 (329.95 to 634.2) | 35.01 (23.7 to 51.05) | 55.58 (36.68 to 80.88) |
| Slovakia | 72.24 (55.83 to 91.11) | 53.06 (40.05 to 67.63) | 5.55 (4.04 to 7.45) | 6.26 (4.45 to 8.58) |
| Slovenia | 10.53 (7.75 to 13.97) | 11.81 (9.23 to 15.28) | 2.57 (1.81 to 3.56) | 3.82 (2.80 to 5.10) |
| Solomon Islands | 4.14 (2.93 to 5.63) | 6.76 (4.76 to 9.32) | 2.66 (1.74 to 3.87) | 2.60 (1.69 to 3.83) |
| Somalia | 204.92 (141.74 to 290.48) | 323.93 (233.52 to 434.34) | 4.97 (3.23 to 7.44) | 3.05 (1.99 to 4.40) |
| South Africa | 548.22 (387.83 to 733.71) | 418.56 (290.85 to 568.26) | 4.00 (2.67 to 5.74) | 2.75 (1.82 to 3.92) |
| South Sudan | 148.54 (101.98 to 212.28) | 151.81 (109.38 to 205.01) | 5.38 (3.44 to 8.03) | 3.50 (2.32 to 5.12) |
| Spain | 182.88 (133.93 to 248.24) | 484.18 (365.64 to 629.31) | 2.43 (1.68 to 3.43) | 7.94 (5.67 to 10.94) |
| Sri Lanka | 1174.9 (866.47 to 1573.48) | 597.27 (440.28 to 802.25) | 21.79 (15.12 to 31.07) | 11.96 (8.32 to 17.23) |
| Sudan | 236.1 (182 to 300.8) | 366.91 (270.34 to 488.38) | 2.57 (1.79 to 3.59) | 2.22 (1.53 to 3.13) |
| Suriname | 6.28 (4.82 to 8.21) | 6.44 (4.91 to 8.35) | 4.84 (3.40 to 6.64) | 4.59 (3.26 to 6.32) |
| Sweden | 528.52 (401.76 to 681.23) | 553.64 (399.42 to 731.7) | 34.54 (24.99 to 46.79) | 30.89 (21.68 to 43.34) |
| Switzerland | 31.45 (24.12 to 40.29) | 68.37 (52.26 to 88.13) | 2.73 (1.94 to 3.67) | 5.19 (3.76 to 7.11) |
| Syrian Arab Republic | 167.41 (127.5 to 216.33) | 92.39 (68.62 to 122.53) | 2.80 (2.00 to 3.84) | 2.59 (1.81 to 3.58) |
| Taiwan (Province of China) | 237.36 (182.79 to 310.11) | 415.55 (299.88 to 569.83) | 4.40 (3.14 to 5.89) | 14.35 (9.81 to 20.8) |
| Tajikistan | 48.33 (32.98 to 69.63) | 72.49 (48.27 to 106.2) | 2.09 (1.30 to 3.13) | 2.03 (1.25 to 3.08) |
| Thailand | 835 (632.22 to 1078.01) | 602.82 (445.93 to 784.2) | 5.04 (3.58 to 6.81) | 6.29 (4.37 to 8.71) |
| Timor-Leste | 14.77 (11.38 to 18.87) | 22.87 (17.45 to 30.04) | 4.29 (3.00 to 5.88) | 4.40 (3.10 to 6.12) |
| Togo | 71.51 (48.92 to 100.49) | 110.18 (77.12 to 151.39) | 3.90 (2.43 to 5.93) | 3.32 (2.14 to 4.94) |
| Tokelau | 0.02 (0.01 to 0.02) | 0.01 (0.01 to 0.02) | 2.97 (2.00 to 4.22) | 2.88 (1.93 to 4.13) |
| Tonga | 1.29 (0.95 to 1.73) | 1.17 (0.84 to 1.55) | 3.08 (2.09 to 4.33) | 2.99 (2.03 to 4.25) |
| Trinidad and Tobago | 42.11 (30.8 to 56.39) | 30.35 (21.87 to 40.98) | 10.36 (7.22 to 14.67) | 11.42 (7.79 to 16.50) |
| Tunisia | 78.81 (60.1 to 101) | 71.79 (53.85 to 93.89) | 2.54 (1.79 to 3.47) | 2.62 (1.82 to 3.60) |
| Turkey | 440.62 (329.59 to 591.71) | 403.11 (292.73 to 540.08) | 2.16 (1.50 to 2.99) | 2.20 (1.51 to 3.04) |
| Turkmenistan | 96.47 (74.84 to 125.05) | 108.91 (82.55 to 141.14) | 6.30 (4.51 to 8.74) | 7.13 (5.03 to 9.98) |
| Tuvalu | 0.1 (0.07 to 0.13) | 0.1 (0.07 to 0.14) | 2.86 (1.91 to 4.09) | 2.72 (1.79 to 3.96) |
| Uganda | 346.14 (239.67 to 480.04) | 470.93 (333.73 to 637.27) | 3.81 (2.48 to 5.67) | 2.36 (1.52 to 3.42) |
| Ukraine | 795.82 (605.28 to 1016.14) | 469.76 (347.93 to 622.52) | 7.00 (5.05 to 9.48) | 7.53 (5.42 to 10.4) |
| United Arab Emirates | 37.94 (29.24 to 49.76) | 72.58 (56.07 to 91.95) | 6.17 (4.47 to 8.59) | 5.49 (3.93 to 7.46) |
| United Kingdom | 2070.11 (1605.46 to 2683.52) | 1932.5 (1452.1 to 2545.18) | 18.97 (13.77 to 26.17) | 16.83 (12.04 to 23.16) |
| United Republic of Tanzania | 604.36 (420.85 to 834.41) | 655.42 (474.74 to 881.11) | 4.75 (3.06 to 6.97) | 2.67 (1.76 to 3.81) |
| United States of America | 14965.17 (11293.44 to 19833.95) | 18474.28 (13570.44 to 24768.27) | 26.76 (19.19 to 36.84) | 32.39 (22.36 to 45.11) |
| United States Virgin Islands | 4.06 (3.07 to 5.32) | 1.83 (1.35 to 2.46) | 12.88 (9.00 to 18.12) | 14.28 (9.96 to 20.31) |
| Uruguay | 58.98 (45.28 to 75.11) | 37.09 (27.94 to 48.38) | 7.35 (5.25 to 10.10) | 5.81 (4.11 to 8.09) |
| Uzbekistan | 527.33 (405.95 to 676.18) | 364.95 (277.67 to 475.89) | 6.07 (4.28 to 8.43) | 3.62 (2.57 to 4.92) |
| Vanuatu | 1.86 (1.33 to 2.51) | 3.11 (2.19 to 4.26) | 2.73 (1.80 to 3.96) | 2.68 (1.75 to 3.91) |
| Venezuela (Bolivarian Republic of) | 297.74 (232.31 to 378.43) | 279.2 (215.06 to 361.17) | 4.19 (2.99 to 5.72) | 4.27 (3.05 to 5.80) |
| Viet Nam | 1572.6 (1186.93 to 2017.04) | 1654.03 (1217.92 to 2174.93) | 5.90 (4.19 to 8.00) | 6.73 (4.71 to 9.46) |
| Yemen | 173.39 (132.24 to 224.6) | 305.54 (229.28 to 405.36) | 2.38 (1.63 to 3.31) | 2.22 (1.53 to 3.14) |
| Zambia | 199.17 (137.41 to 277.02) | 203.43 (143.02 to 275.92) | 4.99 (3.22 to 7.41) | 2.46 (1.60 to 3.52) |
| Zimbabwe | 265.39 (188.51 to 355.51) | 329.05 (232.42 to 446.14) | 5.44 (3.71 to 7.89) | 5.22 (3.43 to 7.76) |

ASR: age-standardized rate**,** DALY: disability-adjusted life years, SDI: sociodemographic index, UI: uncertain interval.

*Number of cases for countries or territories is actual data not divided by 1000.

**Table S3: Number cases and ASR of deaths for childhood myocarditis from 1990 to 2021 by SDI level, region, sex and countries/territories**

| **Characteristics** | **Number of cases (95% UI)** | | **ASR (per 100, 000) (95% UI)** | |
| --- | --- | --- | --- | --- |
|  | **1990** | **2021** | **1990** | **2021** |
| **Global** | 6.34 (4.33 to 8.94) | 2.59 (2.07 to 3.29) | 0.36 (0.24 to 0.51) | 0.13 (0.10 to 0.18) |
| **Age (years)** |  |  |  |  |
| <5 | 5.35 (3.46 to 7.69) | 1.92 (1.47 to 2.54) |  |  |
| 5-9 | 0.59 (0.47 to 0.74) | 0.35 (0.29 to 0.44) |  |  |
| 10-14 | 0.4 (0.32 to 0.51) | 0.32 (0.27 to 0.41) |  |  |
| **Sex** |  |  |  |  |
| Male | 3.47 (2.05 to 5.39) | 1.48 (1.14 to 1.99) | 0.38 (0.22 to 0.60) | 0.15 (0.11 to 0.21) |
| Female | 2.88 (1.77 to 4.43) | 1.11 (0.83 to 1.49) | 0.34 (0.21 to 0.53) | 0.12 (0.09 to 0.16) |
| **SDI level** |  |  |  |  |
| High | 0.38 (0.35 to 0.42) | 0.16 (0.14 to 0.17) | 0.21 (0.19 to 0.23) | 0.10 (0.09 to 0.11) |
| High-middle | 1.16 (0.89 to 1.53) | 0.26 (0.2 to 0.32) | 0.43 (0.33 to 0.59) | 0.12 (0.09 to 0.15) |
| Middle | 3.03 (2.03 to 4.15) | 0.8 (0.62 to 1) | 0.53 (0.35 to 0.72) | 0.15 (0.11 to 0.19) |
| Low-middle | 1.03 (0.57 to 2.1) | 0.74 (0.54 to 1.03) | 0.21 (0.11 to 0.43) | 0.13 (0.10 to 0.19) |
| Low | 0.74 (0.31 to 1.42) | 0.64 (0.43 to 1.04) | 0.29 (0.12 to 0.56) | 0.14 (0.09 to 0.23) |
| **Region** |  |  |  |  |
| Andean Latin America | 0.02 (0.01 to 0.03) | 0 (0 to 0.01) | 0.10 (0.05 to 0.20) | 0.02 (0.01 to 0.03) |
| Australasia | 0.02 (0.01 to 0.02) | 0 (0 to 0.01) | 0.34 (0.30 to 0.38) | 0.09 (0.07 to 0.11) |
| Caribbean | 0.04 (0.02 to 0.09) | 0.04 (0.02 to 0.06) | 0.32 (0.16 to 0.79) | 0.34 (0.17 to 0.60) |
| Central Asia | 0.03 (0.02 to 0.03) | 0.02 (0.02 to 0.02) | 0.10 (0.08 to 0.13) | 0.07 (0.05 to 0.08) |
| Central Europe | 0.09 (0.08 to 0.1) | 0.01 (0.01 to 0.02) | 0.33 (0.29 to 0.37) | 0.08 (0.07 to 0.10) |
| Central Latin America | 0.05 (0.04 to 0.05) | 0.05 (0.04 to 0.07) | 0.07 (0.06 to 0.08) | 0.08 (0.06 to 0.11) |
| Central Sub-Saharan Africa | 0.08 (0.03 to 0.17) | 0.06 (0.03 to 0.1) | 0.29 (0.10 to 0.61) | 0.10 (0.05 to 0.18) |
| East Asia | 3.38 (2.34 to 4.68) | 0.66 (0.46 to 0.84) | 1.02 (0.68 to 1.44) | 0.26 (0.18 to 0.35) |
| Eastern Europe | 0.04 (0.04 to 0.05) | 0.01 (0.01 to 0.01) | 0.09 (0.08 to 0.09) | 0.04 (0.03 to 0.05) |
| Eastern Sub-Saharan Africa | 0.27 (0.07 to 0.54) | 0.15 (0.08 to 0.22) | 0.27 (0.07 to 0.54) | 0.08 (0.04 to 0.13) |
| High-income Asia Pacific | 0.07 (0.06 to 0.08) | 0.02 (0.02 to 0.02) | 0.21 (0.17 to 0.27) | 0.10 (0.09 to 0.12) |
| High-income North America | 0.18 (0.17 to 0.19) | 0.1 (0.09 to 0.11) | 0.29 (0.27 to 0.30) | 0.16 (0.14 to 0.18) |
| North Africa and Middle East | 0.33 (0.16 to 0.8) | 0.17 (0.08 to 0.36) | 0.23 (0.11 to 0.56) | 0.09 (0.05 to 0.22) |
| Oceania | 0.01 (0 to 0.01) | 0.01 (0.01 to 0.03) | 0.27 (0.13 to 0.53) | 0.27 (0.12 to 0.53) |
| South Asia | 0.92 (0.41 to 1.74) | 0.68 (0.45 to 0.91) | 0.21 (0.09 to 0.39) | 0.15 (0.09 to 0.20) |
| South-East Asia Region | 0.29 (0.18 to 0.6) | 0.19 (0.14 to 0.28) | 0.17 (0.11 to 0.37) | 0.11 (0.08 to 0.17) |
| Southern Latin America | 0.03 (0.03 to 0.04) | 0 (0 to 0.01) | 0.23 (0.20 to 0.28) | 0.04 (0.03 to 0.05) |
| Southern Sub-Saharan Africa | 0.03 (0.02 to 0.04) | 0.02 (0.02 to 0.03) | 0.15 (0.08 to 0.21) | 0.10 (0.07 to 0.15) |
| Tropical Latin America | 0.1 (0.08 to 0.11) | 0.05 (0.04 to 0.06) | 0.19 (0.16 to 0.23) | 0.10 (0.07 to 0.12) |
| Western Europe | 0.06 (0.06 to 0.07) | 0.02 (0.02 to 0.02) | 0.09 (0.08 to 0.10) | 0.03 (0.03 to 0.04) |
| Western Sub-Saharan Africa | 0.32 (0.17 to 0.73) | 0.33 (0.2 to 0.64) | 0.32 (0.17 to 0.74) | 0.14 (0.09 to 0.28) |
| **Countries or territories*** |  |  |  |  |
| Afghanistan | 12.78 (1.79 to 48.19) | 23.78 (3.54 to 79) | 0.27 (0.04 to 1.03) | 0.16 (0.02 to 0.51) |
| Albania | 4.77 (2.26 to 7.27) | 1.05 (0.58 to 1.6) | 0.42 (0.19 to 0.65) | 0.25 (0.13 to 0.40) |
| Algeria | 21.18 (3.83 to 73.68) | 8.34 (1.48 to 27.12) | 0.20 (0.03 to 0.7) | 0.06 (0.01 to 0.22) |
| American Samoa | 0.01 (0 to 0.01) | 0 (0 to 0) | 0.04 (0.02 to 0.07) | 0.02 (0.01 to 0.04) |
| Andorra | 0.02 (0.01 to 0.03) | 0 (0 to 0.01) | 0.23 (0.12 to 0.41) | 0.04 (0.02 to 0.07) |
| Angola | 15.84 (4.07 to 44.92) | 16.47 (9.3 to 27.49) | 0.29 (0.07 to 0.86) | 0.10 (0.05 to 0.20) |
| Antigua and Barbuda | 0.04 (0.03 to 0.05) | 0.02 (0.02 to 0.03) | 0.23 (0.18 to 0.30) | 0.15 (0.12 to 0.19) |
| Argentina | 26.64 (22.44 to 31.65) | 3.79 (3 to 4.64) | 0.27 (0.22 to 0.33) | 0.04 (0.03 to 0.05) |
| Armenia | 0.55 (0.34 to 0.82) | 0.06 (0.04 to 0.07) | 0.05 (0.03 to 0.08) | 0.01 (0.01 to 0.01) |
| Australia | 11.84 (10.64 to 12.98) | 3.29 (2.65 to 4.06) | 0.32 (0.28 to 0.37) | 0.07 (0.06 to 0.09) |
| Austria | 0.22 (0.19 to 0.25) | 0.29 (0.23 to 0.36) | 0.02 (0.01 to 0.02) | 0.02 (0.02 to 0.03) |
| Azerbaijan | 4.69 (1.95 to 7.63) | 2.48 (1.52 to 3.63) | 0.19 (0.07 to 0.34) | 0.11 (0.06 to 0.17) |
| Bahamas | 0.07 (0.06 to 0.09) | 0.03 (0.02 to 0.05) | 0.09 (0.06 to 0.13) | 0.05 (0.03 to 0.07) |
| Bahrain | 0.12 (0.08 to 0.2) | 0.05 (0.03 to 0.09) | 0.07 (0.04 to 0.12) | 0.02 (0.01 to 0.03) |
| Bangladesh | 91.56 (31.41 to 224.18) | 45.63 (29.1 to 68.14) | 0.17 (0.05 to 0.44) | 0.11 (0.06 to 0.18) |
| Barbados | 0.05 (0.04 to 0.07) | 0.03 (0.02 to 0.04) | 0.09 (0.07 to 0.12) | 0.07 (0.05 to 0.11) |
| Belarus | 1.76 (1.42 to 2.2) | 0.19 (0.14 to 0.26) | 0.07 (0.06 to 0.10) | 0.01 (0.01 to 0.02) |
| Belgium | 0.38 (0.33 to 0.43) | 0.42 (0.33 to 0.52) | 0.02 (0.02 to 0.03) | 0.02 (0.02 to 0.03) |
| Belize | 0.17 (0.12 to 0.24) | 0.06 (0.05 to 0.08) | 0.20 (0.13 to 0.29) | 0.06 (0.04 to 0.08) |
| Benin | 7.64 (3.51 to 16.21) | 8.55 (3.9 to 16.46) | 0.28 (0.12 to 0.60) | 0.13 (0.05 to 0.26) |
| Bermuda | 0.01 (0 to 0.01) | 0 (0 to 0.01) | 0.06 (0.03 to 0.09) | 0.05 (0.03 to 0.08) |
| Bhutan | 0.58 (0.17 to 1.15) | 0.26 (0.16 to 0.41) | 0.21 (0.06 to 0.44) | 0.15 (0.08 to 0.24) |
| Bolivia (Plurinational State of) | 3.51 (1.32 to 8.93) | 1.43 (0.82 to 2.3) | 0.12 (0.04 to 0.32) | 0.04 (0.02 to 0.07) |
| Bosnia and Herzegovina | 1.59 (0.86 to 2.43) | 0.32 (0.16 to 0.52) | 0.15 (0.08 to 0.25) | 0.07 (0.03 to 0.12) |
| Botswana | 0.82 (0.5 to 1.31) | 0.69 (0.39 to 1.12) | 0.14 (0.08 to 0.23) | 0.10 (0.05 to 0.18) |
| Brazil | 94.25 (78.83 to 113.37) | 46.26 (36.58 to 57.65) | 0.20 (0.16 to 0.24) | 0.10 (0.07 to 0.12) |
| Brunei Darussalam | 0.54 (0.38 to 0.74) | 0.28 (0.2 to 0.39) | 0.58 (0.35 to 0.89) | 0.31 (0.20 to 0.47) |
| Bulgaria | 2.92 (2.55 to 3.35) | 1.1 (0.85 to 1.39) | 0.18 (0.15 to 0.22) | 0.12 (0.09 to 0.16) |
| Burkina Faso | 17.36 (7.23 to 41.13) | 20.26 (9.27 to 42.52) | 0.33 (0.13 to 0.79) | 0.18 (0.08 to 0.37) |
| Burundi | 11.41 (3.15 to 25.2) | 5.63 (2.73 to 10.09) | 0.38 (0.10 to 0.86) | 0.09 (0.04 to 0.18) |
| Cabo Verde | 0.18 (0.06 to 0.48) | 0.06 (0.01 to 0.15) | 0.11 (0.03 to 0.30) | 0.05 (0.01 to 0.12) |
| Cambodia | 7.26 (2.77 to 20.24) | 5.33 (3.27 to 8.74) | 0.14 (0.05 to 0.42) | 0.11 (0.06 to 0.19) |
| Cameroon | 13.68 (6.28 to 25.97) | 18.49 (8.61 to 34.63) | 0.25 (0.11 to 0.47) | 0.13 (0.06 to 0.26) |
| Canada | 8.54 (7.67 to 9.44) | 4.75 (4.06 to 5.49) | 0.15 (0.13 to 0.18) | 0.08 (0.07 to 0.10) |
| Central African Republic | 4.94 (1.8 to 10.91) | 5 (2.24 to 9.27) | 0.35 (0.12 to 0.81) | 0.21 (0.09 to 0.43) |
| Chad | 9.97 (3.86 to 24.95) | 16.92 (5.88 to 46.23) | 0.29 (0.10 to 0.74) | 0.17 (0.05 to 0.45) |
| Chile | 5.32 (4.69 to 6.02) | 0.83 (0.6 to 1.11) | 0.13 (0.11 to 0.15) | 0.03 (0.02 to 0.04) |
| China | 3333.62 (2292.59 to 4634.4) | 640.12 (450.93 to 816.43) | 1.05 (0.70 to 1.48) | 0.26 (0.19 to 0.35) |
| Colombia | 11.07 (9.3 to 13.2) | 12.71 (9.23 to 17.27) | 0.09 (0.07 to 0.12) | 0.12 (0.09 to 0.18) |
| Comoros | 0.71 (0.21 to 1.49) | 0.19 (0.11 to 0.32) | 0.31 (0.08 to 0.66) | 0.08 (0.04 to 0.15) |
| Congo | 2.49 (1.11 to 4.48) | 1.42 (0.81 to 2.31) | 0.22 (0.09 to 0.42) | 0.08 (0.04 to 0.14) |
| Cook Islands | 0 (0 to 0) | 0 (0 to 0) | 0.01 (0 to 0.02) | 0 (0 to 0.01) |
| Costa Rica | 1.3 (1.11 to 1.49) | 0.72 (0.59 to 0.88) | 0.11 (0.09 to 0.14) | 0.07 (0.06 to 0.10) |
| Cote d’Ivoire | 18.24 (8.39 to 39.99) | 17.78 (7.9 to 31.38) | 0.28 (0.12 to 0.63) | 0.15 (0.06 to 0.27) |
| Croatia | 1.87 (1.69 to 2.06) | 0.48 (0.4 to 0.58) | 0.21 (0.18 to 0.24) | 0.09 (0.07 to 0.11) |
| Cuba | 2.85 (2.27 to 3.37) | 1.11 (0.9 to 1.36) | 0.11 (0.08 to 0.14) | 0.07 (0.05 to 0.09) |
| Cyprus | 0.16 (0.08 to 0.28) | 0.03 (0.02 to 0.05) | 0.09 (0.04 to 0.16) | 0.01 (0.01 to 0.03) |
| Czechia | 0.87 (0.77 to 0.98) | 0.3 (0.22 to 0.39) | 0.04 (0.04 to 0.05) | 0.02 (0.01 to 0.02) |
| Democratic People's Republic of Korea | 37.35 (24.39 to 54.92) | 12.79 (6.81 to 21.75) | 0.58 (0.36 to 0.90) | 0.28 (0.14 to 0.49) |
| Democratic Republic of the Congo | 58.34 (22.24 to 123.49) | 37.53 (18.46 to 67.93) | 0.29 (0.10 to 0.64) | 0.1 (0.04 to 0.19) |
| Denmark | 0.42 (0.31 to 0.57) | 0.27 (0.22 to 0.32) | 0.05 (0.03 to 0.07) | 0.03 (0.02 to 0.04) |
| Djibouti | 0.45 (0.12 to 0.94) | 0.29 (0.16 to 0.49) | 0.24 (0.06 to 0.53) | 0.07 (0.03 to 0.12) |
| Dominica | 0.05 (0.04 to 0.08) | 0.03 (0.02 to 0.04) | 0.21 (0.13 to 0.32) | 0.24 (0.14 to 0.39) |
| Dominican Republic | 3.75 (1.84 to 6.92) | 2.48 (1.33 to 4.32) | 0.13 (0.06 to 0.26) | 0.08 (0.04 to 0.16) |
| Ecuador | 3.38 (2.74 to 4.26) | 0.99 (0.72 to 1.32) | 0.09 (0.07 to 0.12) | 0.02 (0.01 to 0.03) |
| Egypt | 6.9 (0.65 to 30.26) | 1.91 (0.25 to 6.75) | 0.03 (0 to 0.13) | 0.01 (0 to 0.02) |
| El Salvador | 1.73 (1.05 to 2.66) | 0.55 (0.29 to 0.99) | 0.08 (0.04 to 0.14) | 0.03 (0.01 to 0.06) |
| Equatorial Guinea | 0.6 (0.22 to 1.25) | 0.36 (0.16 to 0.71) | 0.27 (0.09 to 0.59) | 0.07 (0.03 to 0.14) |
| Eritrea | 5.17 (1.53 to 11.23) | 2.67 (1.4 to 4.44) | 0.29 (0.08 to 0.66) | 0.10 (0.05 to 0.18) |
| Estonia | 0.12 (0.1 to 0.14) | 0 (0 to 0) | 0.03 (0.03 to 0.04) | 0 (0 to 0) |
| Eswatini | 0.71 (0.4 to 1.12) | 0.48 (0.25 to 0.82) | 0.17 (0.09 to 0.30) | 0.12 (0.05 to 0.22) |
| Ethiopia | 69.99 (20.03 to 162.53) | 27.86 (15.93 to 44.35) | 0.26 (0.07 to 0.61) | 0.06 (0.03 to 0.11) |
| Fiji | 0.43 (0.29 to 0.64) | 0.5 (0.3 to 0.79) | 0.16 (0.10 to 0.25) | 0.19 (0.11 to 0.32) |
| Finland | 1.19 (0.95 to 1.47) | 0.51 (0.42 to 0.61) | 0.13 (0.10 to 0.17) | 0.06 (0.05 to 0.08) |
| France | 0.84 (0.73 to 0.96) | 1.65 (1.37 to 1.97) | 0.01 (0.01 to 0.01) | 0.02 (0.01 to 0.02) |
| Gabon | 0.66 (0.28 to 1.23) | 0.37 (0.2 to 0.61) | 0.15 (0.06 to 0.30) | 0.06 (0.03 to 0.11) |
| Gambia | 1.14 (0.51 to 2.4) | 1.18 (0.38 to 2.36) | 0.22 (0.09 to 0.49) | 0.12 (0.04 to 0.25) |
| Georgia | 1.3 (0.82 to 1.87) | 0.16 (0.12 to 0.21) | 0.1 (0.06 to 0.14) | 0.02 (0.01 to 0.03) |
| Germany | 5.44 (4.35 to 7.32) | 4.78 (3.96 to 5.78) | 0.04 (0.03 to 0.06) | 0.04 (0.03 to 0.05) |
| Ghana | 23.38 (12.9 to 39.66) | 19.79 (9.54 to 37.49) | 0.32 (0.16 to 0.56) | 0.15 (0.06 to 0.30) |
| Greece | 0.16 (0.14 to 0.18) | 0.27 (0.21 to 0.35) | 0.01 (0.01 to 0.01) | 0.02 (0.01 to 0.03) |
| Greenland | 0.07 (0.03 to 0.13) | 0.03 (0.01 to 0.05) | 0.48 (0.18 to 0.9) | 0.26 (0.09 to 0.45) |
| Grenada | 0.07 (0.06 to 0.1) | 0.03 (0.02 to 0.03) | 0.22 (0.16 to 0.32) | 0.13 (0.10 to 0.17) |
| Guam | 0.11 (0.08 to 0.15) | 0.06 (0.04 to 0.09) | 0.26 (0.17 to 0.38) | 0.17 (0.11 to 0.27) |
| Guatemala | 6.83 (4.52 to 9.26) | 9.6 (7.09 to 13.49) | 0.16 (0.10 to 0.22) | 0.21 (0.14 to 0.31) |
| Guinea | 12.19 (4.8 to 28.92) | 11.03 (5.47 to 21.61) | 0.38 (0.14 to 0.91) | 0.17 (0.08 to 0.35) |
| Guinea-Bissau | 1.91 (0.87 to 4.28) | 1.38 (0.53 to 2.78) | 0.36 (0.16 to 0.83) | 0.15 (0.05 to 0.31) |
| Guyana | 2.33 (1.87 to 2.86) | 1.21 (0.87 to 1.58) | 0.76 (0.57 to 0.99) | 0.57 (0.40 to 0.78) |
| Haiti | 23.44 (7.49 to 70.56) | 30.34 (13.36 to 54.87) | 0.79 (0.23 to 2.46) | 0.68 (0.27 to 1.34) |
| Honduras | 2.18 (1.29 to 3.68) | 1.49 (0.6 to 3.4) | 0.10 (0.05 to 0.18) | 0.05 (0.02 to 0.11) |
| Hungary | 1.58 (1.33 to 2.1) | 0.37 (0.28 to 0.48) | 0.09 (0.07 to 0.11) | 0.03 (0.02 to 0.04) |
| Iceland | 0.02 (0.02 to 0.03) | 0.02 (0.02 to 0.03) | 0.04 (0.03 to 0.05) | 0.03 (0.03 to 0.04) |
| India | 643.67 (278.44 to 1204.32) | 394.89 (261.16 to 539.09) | 0.19 (0.08 to 0.36) | 0.12 (0.08 to 0.17) |
| Indonesia | 98.41 (45.44 to 264.82) | 83.03 (54.41 to 133.94) | 0.15 (0.07 to 0.42) | 0.13 (0.08 to 0.22) |
| Iran (Islamic Republic of) | 30.07 (9.92 to 61.1) | 3.96 (2.14 to 6.13) | 0.12 (0.04 to 0.26) | 0.02 (0.01 to 0.04) |
| Iraq | 83.65 (39.39 to 133.61) | 50.53 (27.8 to 79.4) | 0.95 (0.43 to 1.64) | 0.40 (0.2 to 0.66) |
| Ireland | 0.38 (0.34 to 0.44) | 0.41 (0.34 to 0.48) | 0.04 (0.03 to 0.05) | 0.04 (0.03 to 0.06) |
| Israel | 0.45 (0.36 to 0.54) | 0.4 (0.31 to 0.52) | 0.03 (0.02 to 0.04) | 0.02 (0.01 to 0.02) |
| Italy | 14.8 (13.89 to 15.62) | 0.79 (0.65 to 0.98) | 0.17 (0.16 to 0.19) | 0.01 (0.01 to 0.01) |
| Jamaica | 0.83 (0.53 to 1.09) | 0.34 (0.25 to 0.47) | 0.10 (0.06 to 0.14) | 0.06 (0.04 to 0.09) |
| Japan | 26.97 (25.9 to 28.08) | 14.61 (13.19 to 16.14) | 0.13 (0.12 to 0.14) | 0.10 (0.09 to 0.11) |
| Jordan | 1.6 (0.97 to 2.44) | 1.23 (0.81 to 1.7) | 0.09 (0.05 to 0.16) | 0.04 (0.02 to 0.06) |
| Kazakhstan | 1.17 (0.79 to 1.61) | 5.62 (4 to 7.71) | 0.02 (0.01 to 0.03) | 0.10 (0.07 to 0.14) |
| Kenya | 10.91 (4 to 19.73) | 5.55 (3.07 to 10.25) | 0.09 (0.03 to 0.16) | 0.03 (0.02 to 0.06) |
| Kiribati | 0.1 (0.05 to 0.21) | 0.11 (0.05 to 0.19) | 0.32 (0.14 to 0.68) | 0.28 (0.11 to 0.49) |
| Kuwait | 1.3 (1.01 to 1.65) | 1.28 (1.03 to 1.61) | 0.23 (0.16 to 0.30) | 0.16 (0.12 to 0.21) |
| Kyrgyzstan | 3.52 (2.76 to 4.21) | 2.63 (2.32 to 2.98) | 0.20 (0.15 to 0.24) | 0.12 (0.10 to 0.14) |
| Lao People's Democratic Republic | 4.86 (1.43 to 19.74) | 4.42 (2.37 to 8.3) | 0.25 (0.06 to 1.00) | 0.19 (0.09 to 0.39) |
| Latvia | 0.12 (0.09 to 0.15) | 0 (0 to 0) | 0.02 (0.01 to 0.03) | 0 |
| Lebanon | 1.47 (0.73 to 2.56) | 0.7 (0.44 to 1.08) | 0.13 (0.06 to 0.25) | 0.06 (0.03 to 0.10) |
| Lesotho | 0.88 (0.56 to 1.32) | 0.67 (0.41 to 1.06) | 0.13 (0.07 to 0.21) | 0.11 (0.05 to 0.20) |
| Liberia | 5.71 (2.06 to 15.56) | 3.16 (1.25 to 6.18) | 0.44 (0.14 to 1.22) | 0.14 (0.05 to 0.29) |
| Libya | 8.59 (1.9 to 26.5) | 2.8 (0.47 to 9.87) | 0.47 (0.1 to 1.5) | 0.22 (0.04 to 0.83) |
| Lithuania | 0.15 (0.13 to 0.17) | 0.01 (0 to 0.01) | 0.02 (0.01 to 0.02) | 0 |
| Luxembourg | 0.02 (0.02 to 0.02) | 0.02 (0.02 to 0.03) | 0.03 (0.02 to 0.04) | 0.02 (0.02 to 0.03) |
| Madagascar | 26.18 (6.99 to 55.47) | 16.99 (8.38 to 29.07) | 0.43 (0.11 to 0.93) | 0.15 (0.06 to 0.27) |
| Malawi | 14.58 (3.14 to 32.33) | 5.05 (2.66 to 8.46) | 0.27 (0.06 to 0.63) | 0.06 (0.03 to 0.12) |
| Malaysia | 10.85 (6.96 to 15.98) | 4.9 (3.44 to 6.65) | 0.16 (0.09 to 0.27) | 0.07 (0.04 to 0.11) |
| Maldives | 0.25 (0.1 to 0.54) | 0.09 (0.06 to 0.14) | 0.22 (0.08 to 0.51) | 0.09 (0.05 to 0.16) |
| Mali | 12.32 (4.92 to 30.19) | 13.6 (5.84 to 29.85) | 0.26 (0.10 to 0.64) | 0.11 (0.04 to 0.24) |
| Malta | 0.11 (0.09 to 0.13) | 0.1 (0.07 to 0.13) | 0.13 (0.10 to 0.16) | 0.15 (0.11 to 0.21) |
| Marshall Islands | 0.03 (0.01 to 0.06) | 0.02 (0.01 to 0.04) | 0.15 (0.07 to 0.30) | 0.13 (0.06 to 0.27) |
| Mauritania | 2.14 (1.06 to 4.2) | 1.69 (0.6 to 3.16) | 0.21 (0.10 to 0.42) | 0.09 (0.03 to 0.18) |
| Mauritius | 0.04 (0.03 to 0.05) | 0.09 (0.07 to 0.12) | 0.01 (0.01 to 0.02) | 0.05 (0.03 to 0.07) |
| Mexico | 12.79 (11.35 to 14.48) | 19.45 (14.71 to 25.58) | 0.04 (0.03 to 0.04) | 0.06 (0.05 to 0.09) |
| Micronesia (Federated States of) | 0.08 (0.04 to 0.15) | 0.03 (0.01 to 0.05) | 0.18 (0.08 to 0.35) | 0.10 (0.04 to 0.20) |
| Monaco | 0 (0 to 0.01) | 0 (0 to 0) | 0.1 (0.05 to 0.19) | 0.03 (0.02 to 0.06) |
| Mongolia | 3 (1.87 to 4.51) | 0.76 (0.48 to 1.16) | 0.32 (0.16 to 0.53) | 0.07 (0.04 to 0.12) |
| Montenegro | 0.37 (0.24 to 0.55) | 0.06 (0.03 to 0.1) | 0.24 (0.14 to 0.39) | 0.05 (0.02 to 0.10) |
| Morocco | 30.5 (4.96 to 107.11) | 9.47 (1.18 to 30.87) | 0.3 (0.05 to 1.10) | 0.10 (0.01 to 0.35) |
| Mozambique | 13.31 (2.14 to 32.72) | 7.42 (3.46 to 13.55) | 0.19 (0.03 to 0.48) | 0.05 (0.02 to 0.1) |
| Myanmar | 38.88 (13.45 to 128.81) | 32.7 (19.63 to 58.52) | 0.27 (0.08 to 0.92) | 0.21 (0.11 to 0.41) |
| Namibia | 0.79 (0.48 to 1.17) | 0.72 (0.37 to 1.24) | 0.13 (0.07 to 0.21) | 0.09 (0.04 to 0.17) |
| Nauru | 0.01 (0 to 0.01) | 0.01 (0 to 0.01) | 0.17 (0.07 to 0.35) | 0.16 (0.07 to 0.30) |
| Nepal | 23.42 (7.96 to 52.96) | 11.82 (6.97 to 18.27) | 0.25 (0.08 to 0.59) | 0.13 (0.07 to 0.21) |
| Netherlands | 0.75 (0.64 to 0.86) | 0.68 (0.57 to 0.82) | 0.03 (0.02 to 0.03) | 0.03 (0.02 to 0.03) |
| New Zealand | 3.18 (2.88 to 3.53) | 1.4 (1.2 to 1.58) | 0.40 (0.35 to 0.45) | 0.15 (0.12 to 0.18) |
| Nicaragua | 2.04 (0.88 to 3.37) | 0.81 (0.46 to 1.31) | 0.11 (0.04 to 0.19) | 0.04 (0.02 to 0.07) |
| Niger | 15.18 (5.01 to 39.62) | 17.34 (7.87 to 33) | 0.32 (0.1 to 0.87) | 0.12 (0.05 to 0.24) |
| Nigeria | 152.64 (78.3 to 372.25) | 153.67 (89.46 to 315.93) | 0.35 (0.17 to 0.84) | 0.15 (0.08 to 0.30) |
| Niue | 0 (0 to 0) | 0 (0 to 0) | 0.15 (0.07 to 0.27) | 0.40 (0.18 to 0.74) |
| North Macedonia | 1.06 (0.53 to 1.97) | 0.1 (0.05 to 0.19) | 0.21 (0.10 to 0.42) | 0.03 (0.01 to 0.06) |
| Northern Mariana Islands | 0.01 (0.01 to 0.01) | 0.01 (0 to 0.01) | 0.06 (0.03 to 0.10) | 0.06 (0.03 to 0.09) |
| Norway | 0.96 (0.89 to 1.03) | 0.21 (0.18 to 0.24) | 0.12 (0.11 to 0.13) | 0.02 (0.02 to 0.03) |
| Oman | 2.42 (1.04 to 4.73) | 1.11 (0.65 to 1.71) | 0.27 (0.11 to 0.55) | 0.09 (0.05 to 0.15) |
| Pakistan | 163.57 (73.59 to 294.62) | 223.92 (124.84 to 349.66) | 0.31 (0.13 to 0.58) | 0.26 (0.14 to 0.44) |
| Palau | 0.01 (0 to 0.01) | 0 (0 to 0) | 0.15 (0.06 to 0.27) | 0.09 (0.04 to 0.15) |
| Palestine | 1.53 (0.76 to 2.76) | 1.36 (0.65 to 2.11) | 0.14 (0.06 to 0.28) | 0.08 (0.03 to 0.13) |
| Panama | 0.48 (0.39 to 0.6) | 0.67 (0.52 to 0.86) | 0.06 (0.04 to 0.08) | 0.06 (0.04 to 0.08) |
| Papua New Guinea | 5.96 (2.57 to 11.93) | 12.79 (5.48 to 23.29) | 0.33 (0.14 to 0.68) | 0.30 (0.12 to 0.61) |
| Paraguay | 1.8 (1.11 to 2.48) | 0.9 (0.55 to 1.52) | 0.10 (0.06 to 0.15) | 0.05 (0.03 to 0.08) |
| Peru | 8.53 (3.31 to 17.27) | 1.63 (0.91 to 2.7) | 0.10 (0.03 to 0.21) | 0.02 (0.01 to 0.03) |
| Philippines | 31.24 (14.79 to 52.42) | 25.31 (18.42 to 34.74) | 0.12 (0.06 to 0.21) | 0.08 (0.05 to 0.11) |
| Poland | 28.25 (26.23 to 30.48) | 2.72 (2.24 to 3.33) | 0.33 (0.31 to 0.36) | 0.05 (0.04 to 0.06) |
| Portugal | 0.41 (0.35 to 0.47) | 0.2 (0.16 to 0.25) | 0.02 (0.02 to 0.03) | 0.02 (0.01 to 0.02) |
| Puerto Rico | 0.17 (0.14 to 0.21) | 0.08 (0.06 to 0.1) | 0.02 (0.01 to 0.02) | 0.02 (0.02 to 0.03) |
| Qatar | 0.16 (0.09 to 0.27) | 0.16 (0.1 to 0.29) | 0.11 (0.06 to 0.21) | 0.03 (0.02 to 0.06) |
| Republic of Korea | 32.78 (21.56 to 46.97) | 5.2 (3.65 to 7.03) | 0.31 (0.18 to 0.48) | 0.09 (0.06 to 0.14) |
| Republic of Moldova | 0.86 (0.68 to 1.07) | 0.06 (0.05 to 0.09) | 0.07 (0.05 to 0.09) | 0.01 (0.01 to 0.02) |
| Romania | 39.23 (30.48 to 46.42) | 6.71 (5.77 to 7.86) | 0.76 (0.57 to 0.94) | 0.24 (0.19 to 0.29) |
| Russian Federation | 34.16 (32.32 to 36.25) | 9.84 (8.26 to 11.44) | 0.1 (0.09 to 0.11) | 0.04 (0.03 to 0.05) |
| Rwanda | 13.69 (3.92 to 31.7) | 3.58 (1.95 to 5.79) | 0.36 (0.10 to 0.85) | 0.07 (0.04 to 0.13) |
| Saint Kitts and Nevis | 0.01 (0 to 0.01) | 0.01 (0 to 0.01) | 0.05 (0.03 to 0.07) | 0.05 (0.03 to 0.09) |
| Saint Lucia | 0.14 (0.11 to 0.17) | 0.05 (0.04 to 0.06) | 0.28 (0.21 to 0.36) | 0.17 (0.12 to 0.24) |
| Saint Vincent and the Grenadines | 0.07 (0.05 to 0.09) | 0.04 (0.03 to 0.05) | 0.18 (0.14 to 0.24) | 0.15 (0.11 to 0.21) |
| Samoa | 0.11 (0.06 to 0.19) | 0.08 (0.04 to 0.13) | 0.16 (0.08 to 0.28) | 0.10 (0.05 to 0.18) |
| San Marino | 0 (0 to 0.01) | 0 (0 to 0) | 0.1 (0.05 to 0.18) | 0.03 (0.02 to 0.06) |
| Sao Tome and Principe | 0.16 (0.07 to 0.35) | 0.07 (0.01 to 0.2) | 0.28 (0.11 to 0.61) | 0.10 (0.02 to 0.28) |
| Saudi Arabia | 26.7 (12.92 to 47.34) | 4.05 (1.82 to 9.19) | 0.39 (0.18 to 0.72) | 0.06 (0.02 to 0.13) |
| Senegal | 12.92 (6.88 to 28.16) | 9.19 (3.2 to 18.01) | 0.32 (0.15 to 0.69) | 0.14 (0.05 to 0.28) |
| Serbia | 2.91 (1.56 to 4.86) | 0.28 (0.12 to 0.65) | 0.14 (0.07 to 0.25) | 0.02 (0.01 to 0.06) |
| Seychelles | 0.04 (0.03 to 0.06) | 0.02 (0.02 to 0.03) | 0.18 (0.12 to 0.27) | 0.10 (0.06 to 0.14) |
| Sierra Leone | 10.55 (3.77 to 27.85) | 8.34 (3.32 to 18.49) | 0.49 (0.16 to 1.31) | 0.22 (0.08 to 0.50) |
| Singapore | 7.63 (6.87 to 8.45) | 1.46 (1.26 to 1.72) | 1.24 (1.06 to 1.44) | 0.18 (0.15 to 0.22) |
| Slovakia | 0.92 (0.68 to 1.24) | 0.29 (0.2 to 0.42) | 0.08 (0.05 to 0.11) | 0.04 (0.02 to 0.05) |
| Slovenia | 0.04 (0.04 to 0.05) | 0.01 (0.01 to 0.02) | 0.01 (0.01 to 0.01) | 0 (0 to 0.01) |
| Solomon Islands | 0.23 (0.1 to 0.42) | 0.26 (0.12 to 0.47) | 0.14 (0.06 to 0.28) | 0.10 (0.04 to 0.20) |
| Somalia | 16.77 (4.7 to 38.4) | 16.11 (7.26 to 30.54) | 0.38 (0.10 to 0.90) | 0.14 (0.06 to 0.28) |
| South Africa | 22.27 (10.87 to 34.34) | 9.33 (6.94 to 13.7) | 0.16 (0.07 to 0.25) | 0.06 (0.04 to 0.10) |
| South Sudan | 11.55 (1.72 to 26.35) | 9.73 (2.79 to 24.02) | 0.4 (0.06 to 0.93) | 0.22 (0.06 to 0.55) |
| Spain | 0.99 (0.88 to 1.12) | 1.51 (1.2 to 1.9) | 0.01 (0.01 to 0.02) | 0.03 (0.02 to 0.03) |
| Sri Lanka | 55.6 (31.74 to 71.37) | 8.25 (5.48 to 12.01) | 1.05 (0.59 to 1.45) | 0.17 (0.1 to 0.26) |
| Sudan | 35.61 (4.24 to 164.04) | 24.43 (3.96 to 82.53) | 0.36 (0.04 to 1.70) | 0.15 (0.02 to 0.53) |
| Suriname | 0.21 (0.1 to 0.33) | 0.15 (0.09 to 0.23) | 0.17 (0.07 to 0.29) | 0.11 (0.06 to 0.19) |
| Sweden | 3.57 (3.13 to 4.06) | 0.91 (0.79 to 1.05) | 0.23 (0.19 to 0.27) | 0.05 (0.04 to 0.06) |
| Switzerland | 0.27 (0.21 to 0.36) | 0.17 (0.14 to 0.21) | 0.02 (0.02 to 0.03) | 0.01 (0.01 to 0.02) |
| Syrian Arab Republic | 18.93 (3.95 to 55.13) | 3.15 (0.41 to 13.44) | 0.31 (0.06 to 0.93) | 0.1 (0.01 to 0.41) |
| Taiwan (Province of China) | 4.09 (3.64 to 4.61) | 3.44 (2.94 to 4.01) | 0.08 (0.07 to 0.1) | 0.12 (0.09 to 0.15) |
| Tajikistan | 0.07 (0.02 to 0.15) | 0.08 (0.04 to 0.15) | 0 (0 to 0.01) | 0 |
| Thailand | 16.56 (9.89 to 28.43) | 7.27 (4.74 to 9.64) | 0.10 (0.05 to 0.2) | 0.08 (0.04 to 0.12) |
| Timor-Leste | 0.81 (0.28 to 2.98) | 0.99 (0.55 to 1.82) | 0.21 (0.07 to 0.78) | 0.19 (0.09 to 0.38) |
| Togo | 5.14 (2.53 to 9.83) | 3.72 (1.63 to 6.56) | 0.27 (0.12 to 0.54) | 0.11 (0.04 to 0.21) |
| Tokelau | 0 (0 to 0) | 0 (0 to 0) | 0.13 (0.05 to 0.27) | 0.50 (0.20 to 1.00) |
| Tonga | 0.04 (0.02 to 0.07) | 0.03 (0.01 to 0.05) | 0.10 (0.05 to 0.17) | 0.07 (0.03 to 0.14) |
| Trinidad and Tobago | 1.36 (1.15 to 1.59) | 0.53 (0.41 to 0.69) | 0.35 (0.26 to 0.43) | 0.21 (0.16 to 0.29) |
| Tunisia | 5.59 (1.07 to 19.25) | 1.18 (0.16 to 4.04) | 0.18 (0.03 to 0.65) | 0.04 (0.01 to 0.16) |
| Turkey | 17.23 (6.14 to 38.17) | 4.46 (2.1 to 7.18) | 0.09 (0.03 to 0.20) | 0.03 (0.01 to 0.05) |
| Turkmenistan | 2.84 (2 to 3.7) | 2.54 (2.05 to 3.2) | 0.18 (0.12 to 0.24) | 0.17 (0.12 to 0.22) |
| Tuvalu | 0.01 (0 to 0.02) | 0 (0 to 0.01) | 0.21 (0.09 to 0.41) | 0.10 (0.05 to 0.19) |
| Uganda | 32.03 (7.75 to 73.53) | 18.47 (9.21 to 34.96) | 0.32 (0.07 to 0.74) | 0.09 (0.04 to 0.18) |
| Ukraine | 5.74 (4.47 to 7.21) | 2.67 (2.13 to 3.38) | 0.05 (0.04 to 0.07) | 0.05 (0.03 to 0.06) |
| United Arab Emirates | 1.52 (0.74 to 2.86) | 0.78 (0.5 to 1.37) | 0.24 (0.11 to 0.47) | 0.06 (0.03 to 0.11) |
| United Kingdom | 28.92 (26.39 to 32.27) | 7.6 (6.51 to 8.73) | 0.26 (0.24 to 0.30) | 0.07 (0.06 to 0.08) |
| United Republic of Tanzania | 34.97 (8.83 to 74.67) | 20.67 (11.23 to 34.01) | 0.26 (0.06 to 0.57) | 0.08 (0.04 to 0.15) |
| United States of America | 168.02 (158.32 to 178.55) | 93.16 (83.51 to 103.84) | 0.30 (0.28 to 0.32) | 0.17 (0.15 to 0.19) |
| United States Virgin Islands | 0.08 (0.06 to 0.13) | 0.01 (0.01 to 0.02) | 0.26 (0.15 to 0.43) | 0.10 (0.05 to 0.18) |
| Uruguay | 2.44 (2.12 to 2.84) | 0.33 (0.24 to 0.43) | 0.31 (0.26 to 0.37) | 0.06 (0.04 to 0.08) |
| Uzbekistan | 9.4 (6.32 to 12.18) | 4.56 (3.46 to 6) | 0.10 (0.06 to 0.14) | 0.04 (0.03 to 0.06) |
| Vanuatu | 0.13 (0.07 to 0.23) | 0.16 (0.08 to 0.28) | 0.18 (0.09 to 0.33) | 0.14 (0.06 to 0.26) |
| Venezuela (Bolivarian Republic of) | 7.65 (6.55 to 9.5) | 4.86 (3.5 to 6.6) | 0.11 (0.08 to 0.14) | 0.08 (0.05 to 0.11) |
| Viet Nam | 26.85 (16.15 to 45.18) | 15.45 (8.14 to 28.67) | 0.10 (0.05 to 0.18) | 0.06 (0.03 to 0.13) |
| Yemen | 22.17 (3.02 to 98.07) | 21.23 (3.38 to 70.91) | 0.28 (0.04 to 1.28) | 0.16 (0.02 to 0.54) |
| Zambia | 10.48 (2.24 to 26.71) | 4.69 (2.42 to 7.81) | 0.25 (0.05 to 0.64) | 0.06 (0.02 to 0.10) |
| Zimbabwe | 5.82 (3.73 to 8.76) | 11.38 (6.25 to 17.82) | 0.12 (0.07 to 0.20) | 0.18 (0.09 to 0.31) |

ASR: age-standardized rate**,** SDI: sociodemographic index, UI: uncertain interval.

*Number of cases for countries or territories is actual data not divided by 1000.

**Table S4: Number cases and ASR of DALYs for childhood myocarditis from 1990 to 2021 by SDI level, region, sex and countries/territories**

| **Characteristics** | **Number of cases (95% UI)** | | **ASR (per 100, 000) (95% UI)** | |
| --- | --- | --- | --- | --- |
|  | **1990** | **2021** | **1990** | **2021** |
| **Global** | 566.23 (388.29 to 791.17) | 236 (192.4 to 299.99) | 32.1 (21.65 to 45.06) | 12.24 (9.68 to 15.97) |
| **Age (years)** |  |  |  |  |
| <5 | 481.71 (313 to 689.23) | 176.17 (136.61 to 232.62) |  |  |
| 5-9 | 51.85 (42.52 to 65.16) | 32.71 (27.41 to 40.11) |  |  |
| 10-14 | 32.67 (26.07 to 40.94) | 27.13 (22.88 to 34.71) |  |  |
| **Sex** |  |  |  |  |
| Male | 309.53 (184.19 to 479.85) | 134.07 (104.68 to 179.16) | 34.05 (20.08 to 53.01) | 13.49 (10.2 to 18.5) |
| Female | 256.71 (158.57 to 394.53) | 101.93 (77.35 to 134.76) | 30.02 (18.41 to 47.18) | 10.91 (8.15 to 14.57) |
| **SDI level** |  |  |  |  |
| High | 34.98 (32.25 to 38.67) | 16.98 (15.18 to 19) | 19.45 (17.71 to 21.7) | 10.41 (9.15 to 11.95) |
| High-middle | 103.03 (79.68 to 136.11) | 23.62 (18.62 to 28.87) | 38.56 (29.29 to 52.62) | 10.95 (8.43 to 13.66) |
| Middle | 268.57 (180.18 to 365.76) | 71.55 (55.61 to 88.67) | 46.8 (30.84 to 64.01) | 13.42 (10.23 to 16.9) |
| Low-middle | 93.28 (52.33 to 187.12) | 66.3 (49.69 to 92.1) | 18.99 (10.4 to 37.93) | 11.92 (8.75 to 16.89) |
| Low | 66.01 (27.93 to 126.48) | 57.31 (39.22 to 92.42) | 25.91 (10.79 to 49.58) | 12.18 (8.14 to 20.03) |
| **Region** |  |  |  |  |
| Andean Latin America | 1.38 (0.71 to 2.51) | 0.38 (0.27 to 0.51) | 9.18 (4.47 to 17.26) | 2.12 (1.44 to 3.02) |
| Australasia | 1.4 (1.28 to 1.51) | 0.53 (0.44 to 0.62) | 31.27 (27.78 to 35.27) | 9.72 (7.9 to 12.00) |
| Caribbean | 3.29 (1.76 to 8.02) | 3.37 (1.87 to 5.58) | 28.13 (14.37 to 69.95) | 30.17 (15.70 to 53.37) |
| Central Asia | 2.41 (2.01 to 2.87) | 1.73 (1.43 to 2.09) | 9.18 (7.37 to 11.27) | 6.17 (5.01 to 7.60) |
| Central Europe | 7.96 (7.14 to 8.74) | 1.41 (1.22 to 1.65) | 29.71 (26.19 to 33.20) | 8.42 (7.01 to 10.13) |
| Central Latin America | 4.16 (3.73 to 4.74) | 4.59 (3.58 to 5.99) | 6.38 (5.64 to 7.37) | 7.63 (5.79 to 10.07) |
| Central Sub-Saharan Africa | 7.36 (2.82 to 14.99) | 5.4 (2.98 to 8.78) | 25.59 (9.02 to 53.54) | 9.05 (4.71 to 16.03) |
| East Asia | 297.8 (205.94 to 413.09) | 58.48 (41.84 to 74.27) | 90.19 (60.45 to 127.09) | 23.54 (16.7 to 30.69) |
| Eastern Europe | 4.12 (3.85 to 4.49) | 1.27 (1.08 to 1.48) | 8.16 (7.51 to 8.95) | 3.81 (3.16 to 4.53) |
| Eastern Sub-Saharan Africa | 24.49 (6.87 to 48.38) | 13.07 (7.56 to 19.77) | 24.08 (6.65 to 47.89) | 7.20 (3.94 to 11.45) |
| High-income Asia Pacific | 6.13 (5.19 to 7.37) | 2.35 (2.04 to 2.68) | 19.06 (15.41 to 23.83) | 11.21 (9.51 to 13.24) |
| High-income North America | 16.76 (15.66 to 17.87) | 10.28 (9.17 to 11.59) | 27.10 (25.16 to 29.20) | 16.71 (14.58 to 19.20) |
| North Africa and Middle East | 29.64 (14.38 to 70.72) | 15.1 (7.77 to 31.78) | 20.41 (9.62 to 49.22) | 8.53 (4.27 to 19.17) |
| Oceania | 0.69 (0.35 to 1.28) | 1.31 (0.61 to 2.29) | 24.46 (11.91 to 46.71) | 24.11 (10.77 to 46.42) |
| South Asia | 83.58 (38.05 to 154.73) | 61.15 (41.26 to 81.77) | 18.73 (8.41 to 34.62) | 13.15 (8.62 to 18.1) |
| South-East Asia Region | 25.98 (16.66 to 53.16) | 16.8 (12.6 to 24.43) | 15.43 (9.67 to 32.31) | 10.06 (7.19 to 15.51) |
| Southern Latin America | 3.1 (2.68 to 3.59) | 0.47 (0.38 to 0.58) | 21.01 (17.77 to 24.8) | 3.63 (2.81 to 4.62) |
| Southern Sub-Saharan Africa | 2.78 (1.67 to 3.89) | 2.06 (1.47 to 3.04) | 13.18 (7.62 to 18.82) | 8.76 (5.91 to 13.51) |
| Tropical Latin America | 8.64 (7.28 to 10.32) | 4.35 (3.44 to 5.32) | 17.38 (14.41 to 20.99) | 8.79 (6.85 to 10.97) |
| Western Europe | 5.66 (5.23 to 6.13) | 2.43 (2.11 to 2.81) | 8.33 (7.64 to 9.13) | 3.74 (3.17 to 4.46) |
| Western Sub-Saharan Africa | 28.91 (15.63 to 65.3) | 29.48 (18.2 to 56.71) | 29.01 (15.19 to 66.11) | 13.06 (7.92 to 25.31) |
| **Countries or territories*** |  |  |  |  |
| Afghanistan | 1136.53 (163.77 to 4277.79) | 2115.03 (334.47 to 6925.76) | 23.67 (3.30 to 91.13) | 13.78 (2.06 to 44.88) |
| Albania | 429.03 (207 to 649.67) | 96 (54.43 to 143.85) | 37.42 (17.43 to 57.59) | 23.09 (12.39 to 36.23) |
| Algeria | 1886.02 (355.3 to 6509.74) | 750.4 (152.86 to 2397.08) | 17.65 (3.22 to 61.81) | 5.60 (1.09 to 19.22) |
| American Samoa | 0.7 (0.38 to 1.2) | 0.26 (0.14 to 0.42) | 3.30 (1.66 to 5.97) | 2.21 (1.03 to 3.86) |
| Andorra | 1.75 (1.06 to 2.85) | 0.43 (0.3 to 0.6) | 20.94 (11.32 to 36.71) | 4.76 (3.05 to 7.09) |
| Angola | 1408.29 (368.98 to 3972.68) | 1456.81 (826.35 to 2419.79) | 26.08 (6.56 to 75.06) | 9.21 (4.63 to 17.19) |
| Antigua and Barbuda | 3.74 (3.04 to 4.56) | 2.19 (1.88 to 2.57) | 21.06 (16.37 to 26.81) | 13.82 (11.19 to 16.98) |
| Argentina | 2390.68 (2016.8 to 2839.47) | 355.3 (287.71 to 431.67) | 24.24 (19.79 to 29.71) | 3.91 (2.95 to 5.02) |
| Armenia | 51.5 (32.95 to 74.58) | 5.86 (4.56 to 7.53) | 4.80 (3.04 to 6.98) | 1.02 (0.77 to 1.36) |
| Australia | 1105.12 (995.94 to 1221.61) | 376.33 (302.13 to 458.34) | 30.11 (26.1 to 34.83) | 8.39 (6.45 to 10.68) |
| Austria | 21.51 (18.76 to 24.53) | 32.98 (27.27 to 40.31) | 1.64 (1.35 to 2.01) | 2.60 (2.01 to 3.28) |
| Azerbaijan | 415.5 (178.84 to 676.5) | 221.05 (138.68 to 321.54) | 16.65 (6.87 to 29.38) | 9.64 (5.55 to 15.11) |
| Bahamas | 6.5 (5.16 to 8.19) | 3.23 (2.38 to 4.31) | 8.54 (5.98 to 11.72) | 4.66 (3.20 to 6.64) |
| Bahrain | 10.67 (6.91 to 17.7) | 4.91 (3.1 to 8.35) | 6.19 (3.65 to 11.02) | 1.73 (0.99 to 3.07) |
| Bangladesh | 8235.4 (2925.77 to 19891.65) | 4089.27 (2674.15 to 6017.15) | 15.56 (5.05 to 38.92) | 9.51 (5.48 to 15.7) |
| Barbados | 4.89 (3.96 to 6.02) | 3.04 (2.24 to 4.1) | 8.26 (6.13 to 10.94) | 7.09 (4.86 to 10.03) |
| Belarus | 166.63 (136.97 to 203.86) | 22.5 (17.18 to 29.02) | 7.08 (5.47 to 9.02) | 1.55 (1.12 to 2.17) |
| Belgium | 35.99 (31.42 to 41.2) | 48.71 (39.72 to 60.59) | 2.06 (1.70 to 2.52) | 2.67 (2.07 to 3.47) |
| Belize | 15.35 (10.84 to 21.11) | 5.97 (4.67 to 7.73) | 18.32 (12.19 to 26.15) | 5.23 (3.87 to 7.09) |
| Benin | 685.24 (318.46 to 1442.43) | 770.49 (357.4 to 1470.89) | 24.79 (10.8 to 53.23) | 11.7 (4.79 to 23.34) |
| Bermuda | 0.69 (0.47 to 0.96) | 0.4 (0.28 to 0.57) | 5.73 (3.54 to 8.34) | 5.14 (3.50 to 7.84) |
| Bhutan | 52.29 (16.43 to 102.79) | 23.52 (14.33 to 36.22) | 19.26 (5.82 to 39.48) | 13.23 (7.29 to 21.45) |
| Bolivia (Plurinational State of) | 312.35 (119.86 to 788.97) | 129.03 (75.89 to 204.16) | 11.00 (3.71 to 28.38) | 3.76 (2.04 to 6.46) |
| Bosnia and Herzegovina | 140.44 (77.93 to 212.56) | 29.11 (15.11 to 45.98) | 13.45 (7.08 to 21.6) | 6.28 (3.10 to 10.56) |
| Botswana | 72.23 (45.19 to 115.34) | 61.32 (34.94 to 98.38) | 12.02 (6.75 to 20.25) | 8.99 (4.45 to 15.98) |
| Brazil | 8476.08 (7125.57 to 10171.78) | 4263.96 (3373.85 to 5233.39) | 17.67 (14.58 to 21.45) | 8.97 (6.98 to 11.2) |
| Brunei Darussalam | 47.69 (34.24 to 64.9) | 26.21 (18.71 to 36.04) | 50.99 (31.98 to 77.09) | 28.89 (18.67 to 42.58) |
| Bulgaria | 262.78 (229.96 to 300.57) | 104.08 (81.6 to 129.78) | 16.58 (13.97 to 19.64) | 11.40 (8.58 to 14.93) |
| Burkina Faso | 1556.46 (652.09 to 3668.16) | 1825.38 (847.74 to 3794.47) | 29.47 (11.69 to 70.48) | 15.81 (7.00 to 33.3) |
| Burundi | 1016.25 (287.31 to 2237.07) | 504.86 (247.82 to 898.84) | 33.70 (9.04 to 75.98) | 8.27 (3.75 to 15.89) |
| Cabo Verde | 17.29 (6.54 to 44) | 6 (1.68 to 13.81) | 10.27 (3.69 to 26.93) | 4.60 (1.15 to 11.21) |
| Cambodia | 645.83 (252.39 to 1799.68) | 473.81 (296.14 to 774.9) | 12.79 (4.55 to 36.79) | 9.39 (5.16 to 16.6) |
| Cameroon | 1224.68 (571.55 to 2307.02) | 1660.82 (788.75 to 3079.76) | 21.95 (9.78 to 41.84) | 12.02 (5.37 to 22.76) |
| Canada | 823.48 (743.61 to 916.11) | 584.04 (483.24 to 708.69) | 14.69 (12.48 to 17.11) | 10.03 (7.84 to 13.01) |
| Central African Republic | 437.92 (160.07 to 965.39) | 439.59 (198.47 to 814.68) | 31.35 (10.71 to 71.08) | 18.60 (7.74 to 37.39) |
| Chad | 890.96 (351.15 to 2221.89) | 1517.08 (541.23 to 4099.17) | 25.89 (9.20 to 65.31) | 15.00 (4.92 to 39.68) |
| Chile | 484.42 (426.86 to 546.69) | 84.4 (63.14 to 108.54) | 11.86 (10.16 to 13.84) | 2.56 (1.83 to 3.49) |
| China | 294138.77 (201842.13 to 408935.49) | 57042.39 (40816.18 to 72392.72) | 92.23 (61.53 to 130.07) | 23.66 (16.74 to 30.79) |
| Colombia | 993.87 (840.8 to 1180.4) | 1153.71 (847.4 to 1557.64) | 8.36 (6.70 to 10.41) | 11.33 (7.94 to 15.82) |
| Comoros | 63.72 (18.76 to 132.66) | 17.45 (9.92 to 28.85) | 27.39 (7.55 to 58.1) | 7.46 (3.90 to 12.92) |
| Congo | 221.25 (99.11 to 397.22) | 125.75 (72.01 to 202.8) | 19.91 (8.35 to 37.22) | 6.78 (3.56 to 11.9) |
| Cook Islands | 0.05 (0.02 to 0.09) | 0.01 (0.01 to 0.02) | 0.75 (0.29 to 1.57) | 0.33 (0.14 to 0.77) |
| Costa Rica | 119.43 (102.97 to 137.59) | 68.26 (57.53 to 82.41) | 10.43 (8.51 to 12.55) | 7.09 (5.47 to 8.94) |
| Cote d’Ivoire | 1631.67 (757.11 to 3565.11) | 1595.6 (718.93 to 2794.54) | 25.29 (10.95 to 56.22) | 13.01 (5.45 to 24.25) |
| Croatia | 171.59 (156.42 to 189.44) | 53.2 (44.66 to 64.41) | 18.89 (16.45 to 21.72) | 9.41 (7.42 to 11.92) |
| Cuba | 258.36 (208.09 to 303.36) | 106.55 (86.77 to 130.86) | 10.2 (7.77 to 12.63) | 6.54 (5.08 to 8.30) |
| Cyprus | 15.25 (7.92 to 25.67) | 3.57 (2.41 to 5.52) | 8.10 (3.76 to 14.96) | 1.64 (0.98 to 2.70) |
| Czechia | 85.85 (76.07 to 96.66) | 42.82 (33.46 to 55.21) | 4.41 (3.75 to 5.17) | 2.57 (1.91 to 3.53) |
| Democratic People's Republic of Korea | 3294.41 (2153.91 to 4839.06) | 1115.28 (610.29 to 1867.63) | 51.06 (31.64 to 78.74) | 24.52 (12.34 to 42.29) |
| Democratic Republic of the Congo | 5184.9 (1995.09 to 10954.86) | 3315.87 (1660.15 to 5938.56) | 25.65 (9.02 to 56.84) | 8.62 (3.99 to 16.85) |
| Denmark | 39.12 (29.15 to 51.88) | 31.19 (25.96 to 37.34) | 4.62 (3.26 to 6.39) | 3.40 (2.67 to 4.31) |
| Djibouti | 40.06 (11.15 to 83.71) | 26 (14.2 to 43.35) | 21.85 (5.74 to 47.33) | 6.26 (3.18 to 10.87) |
| Dominica | 4.67 (3.35 to 6.64) | 2.45 (1.57 to 3.57) | 18.87 (12.07 to 28.35) | 21.61 (12.68 to 34.66) |
| Dominican Republic | 336.46 (166.74 to 616.65) | 223.77 (123.99 to 385.56) | 11.98 (5.32 to 22.9) | 7.60 (3.69 to 14.22) |
| Ecuador | 300.89 (245.48 to 375.87) | 92.06 (70.27 to 119.6) | 7.81 (6.07 to 10.17) | 1.87 (1.28 to 2.65) |
| Egypt | 863.57 (275.94 to 2940.37) | 392.69 (177.63 to 811.06) | 3.63 (1.10 to 12.28) | 1.06 (0.45 to 2.31) |
| El Salvador | 154.31 (94.14 to 235.95) | 50.02 (27.84 to 87.73) | 7.07 (3.66 to 11.97) | 2.81 (1.38 to 5.42) |
| Equatorial Guinea | 53.56 (20 to 109.89) | 32.42 (14.44 to 61.49) | 23.71 (8.02 to 51.67) | 5.87 (2.44 to 12.2) |
| Eritrea | 461.07 (140.33 to 992.46) | 237.88 (126.08 to 393.67) | 26.27 (7.58 to 57.67) | 9.14 (4.59 to 16.16) |
| Estonia | 11.29 (9.44 to 13.24) | 0.55 (0.4 to 0.76) | 3.26 (2.61 to 3.99) | 0.26 (0.18 to 0.37) |
| Eswatini | 62.45 (35.99 to 99.1) | 42.33 (22.85 to 71.19) | 15.41 (8.03 to 26.32) | 10.43 (4.84 to 18.85) |
| Ethiopia | 6381.25 (1988.72 to 14630.58) | 2562.76 (1501.49 to 4007.98) | 23.5 (7.09 to 54.41) | 5.66 (3.20 to 9.49) |
| Fiji | 37.75 (25.89 to 55.88) | 44.14 (26.7 to 68.58) | 13.85 (8.69 to 21.58) | 16.69 (9.41 to 28.14) |
| Finland | 110.02 (90.35 to 135.34) | 59.19 (49.64 to 71.94) | 11.84 (9.14 to 15.26) | 7.44 (5.79 to 9.40) |
| France | 93.33 (80.64 to 107.52) | 228.05 (187.34 to 279.85) | 0.83 (0.68 to 0.99) | 2.12 (1.65 to 2.72) |
| Gabon | 59.08 (25.2 to 109.29) | 32.72 (18.32 to 53.78) | 13.54 (5.62 to 26.35) | 5.25 (2.54 to 9.53) |
| Gambia | 101.82 (46.64 to 213.35) | 105.44 (35.96 to 208.46) | 19.75 (8.47 to 43) | 10.39 (3.33 to 21.67) |
| Georgia | 121.89 (79.62 to 171.87) | 16.38 (12.63 to 20.98) | 9.02 (5.52 to 12.99) | 2.27 (1.64 to 3.12) |
| Germany | 512.13 (416.77 to 676.88) | 513.02 (429.32 to 605.53) | 3.98 (3.00 to 5.58) | 4.34 (3.43 to 5.49) |
| Ghana | 2077.89 (1159.76 to 3505.24) | 1758.66 (848.34 to 3331.78) | 28.23 (14.81 to 49.23) | 13.35 (5.74 to 26.64) |
| Greece | 16.25 (14.14 to 18.81) | 28.88 (23.32 to 36.07) | 0.92 (0.76 to 1.10) | 2.21 (1.70 to 2.93) |
| Greenland | 6.67 (2.75 to 11.83) | 2.82 (1.08 to 4.7) | 43.14 (16.97 to 80.42) | 24.36 (9.00 to 41.48) |
| Grenada | 6.66 (5.07 to 9.2) | 2.41 (1.94 to 2.99) | 19.63 (13.98 to 28.31) | 11.66 (8.78 to 15.31) |
| Guam | 10 (7.36 to 13.25) | 5.69 (3.74 to 8.43) | 22.55 (15.37 to 33.14) | 15.52 (9.92 to 24.06) |
| Guatemala | 616.04 (413.68 to 823.9) | 864.15 (638.88 to 1206.52) | 14.34 (9.43 to 19.78) | 18.88 (13.19 to 27.41) |
| Guinea | 1089.89 (432.29 to 2571.77) | 989.43 (498.16 to 1929.35) | 33.66 (12.81 to 80.58) | 15.54 (7.15 to 31.5) |
| Guinea-Bissau | 169.98 (77.88 to 379.03) | 123.11 (48.59 to 247.24) | 32.21 (14.08 to 73.77) | 13.11 (4.88 to 27.38) |
| Guyana | 206.45 (165.25 to 253.69) | 107.22 (77.94 to 139.51) | 66.86 (50.38 to 87.23) | 50.31 (35.67 to 68.44) |
| Haiti | 2076.3 (673.88 to 6245.39) | 2684.66 (1197.49 to 4859.29) | 70.16 (21.01 to 216.95) | 60.3 (24.69 to 117.18) |
| Honduras | 193.47 (117.07 to 324.4) | 135.23 (58.23 to 300.01) | 8.46 (4.54 to 15.33) | 4.23 (1.65 to 9.68) |
| Hungary | 144.64 (122.6 to 189.89) | 39.95 (31.05 to 50.52) | 7.79 (6.38 to 10.21) | 2.98 (2.18 to 3.95) |
| Iceland | 2.27 (1.92 to 2.65) | 2.42 (2.02 to 2.93) | 3.66 (2.81 to 4.68) | 3.66 (2.81 to 4.76) |
| India | 58390.11 (26263.64 to 107509.73) | 35946.17 (24372.6 to 48406.88) | 17.62 (7.78 to 32.33) | 10.96 (7.18 to 15.12) |
| Indonesia | 8796.01 (4263.7 to 23539.59) | 7390.05 (4915 to 11757.1) | 13.47 (6.14 to 37.09) | 11.39 (7.05 to 19.57) |
| Iran (Islamic Republic of) | 2687.3 (890.06 to 5440.35) | 365.61 (211.29 to 549.03) | 10.68 (3.34 to 22.75) | 1.91 (0.88 to 3.32) |
| Iraq | 7404.76 (3495.96 to 11814.38) | 4516.81 (2484.94 to 7056.26) | 84.36 (38.16 to 144.23) | 35.97 (18.51 to 58.35) |
| Ireland | 35.4 (31.4 to 40.14) | 51.8 (42.78 to 63.74) | 3.92 (3.20 to 4.78) | 5.55 (4.26 to 7.29) |
| Israel | 42.46 (34.99 to 50.7) | 48.11 (38.25 to 59.2) | 2.84 (2.12 to 3.76) | 1.83 (1.39 to 2.39) |
| Italy | 1360.16 (1274.07 to 1447.27) | 108.43 (86.93 to 134.94) | 16.03 (14.73 to 17.35) | 1.51 (1.17 to 1.98) |
| Jamaica | 74.67 (49.25 to 97.64) | 32.35 (24.34 to 43.02) | 9.19 (5.64 to 12.89) | 6.08 (4.36 to 8.52) |
| Japan | 2471.89 (2355.76 to 2601.52) | 1614.14 (1411.67 to 1858.93) | 11.84 (11.12 to 12.67) | 11.08 (9.41 to 13.2) |
| Jordan | 143.66 (88.02 to 217.93) | 114.58 (77.28 to 155.63) | 8.49 (4.78 to 13.93) | 3.48 (2.17 to 5.19) |
| Kazakhstan | 113.95 (79.38 to 153.35) | 517.08 (376.11 to 694.47) | 2.15 (1.4 to 3.00) | 9.42 (6.71 to 13.04) |
| Kenya | 988.22 (380.3 to 1761.53) | 510.66 (294.49 to 907.37) | 8.18 (3.06 to 14.62) | 2.91 (1.64 to 5.18) |
| Kiribati | 9.06 (4.37 to 18.59) | 9.89 (4.33 to 16.46) | 28.17 (12.11 to 59.34) | 23.87 (9.74 to 42.08) |
| Kuwait | 116.29 (91.18 to 146.4) | 119.85 (97.24 to 149.6) | 20.27 (14.94 to 26.68) | 15.17 (11.54 to 19.84) |
| Kyrgyzstan | 318.22 (250.47 to 381.42) | 241.55 (215.01 to 270.56) | 17.88 (13.76 to 21.95) | 10.65 (9.00 to 12.54) |
| Lao People's Democratic Republic | 429.51 (129.34 to 1750.15) | 386.77 (208.46 to 728.81) | 21.82 (5.75 to 87.85) | 16.58 (7.76 to 33.75) |
| Latvia | 11.49 (8.99 to 14.06) | 0.72 (0.52 to 1.01) | 2.00 (1.49 to 2.66) | 0.25 (0.18 to 0.35) |
| Lebanon | 134.1 (67.93 to 230.38) | 67.25 (44.92 to 102.75) | 12.26 (5.74 to 22.38) | 5.60 (3.36 to 9.28) |
| Lesotho | 77.99 (49.92 to 116.07) | 59.09 (36.01 to 91.98) | 11.20 (6.38 to 18.57) | 9.79 (4.92 to 17.7) |
| Liberia | 509.77 (185.44 to 1384.94) | 283.48 (116.08 to 552.05) | 39.28 (12.74 to 108.35) | 12.95 (5.01 to 25.91) |
| Libya | 768.84 (173.5 to 2359.95) | 250.19 (46.98 to 872.86) | 42.27 (9.43 to 133.62) | 19.94 (3.62 to 73.63) |
| Lithuania | 14.5 (12.63 to 16.77) | 1.18 (0.83 to 1.6) | 1.75 (1.39 to 2.20) | 0.29 (0.20 to 0.42) |
| Luxembourg | 1.94 (1.69 to 2.24) | 3.32 (2.63 to 4.29) | 2.96 (2.40 to 3.65) | 3.40 (2.56 to 4.60) |
| Madagascar | 2332.41 (648.37 to 4922.37) | 1508.52 (741.27 to 2578.81) | 38.35 (10.26 to 82.21) | 12.9 (5.74 to 23.42) |
| Malawi | 1309.07 (296.41 to 2889.49) | 454.61 (244.52 to 757.02) | 24.52 (5.46 to 55.68) | 5.78 (2.86 to 10.35) |
| Malaysia | 977.06 (638.26 to 1416.04) | 460.24 (336.62 to 615.1) | 14.59 (8.55 to 23.62) | 6.24 (3.84 to 9.72) |
| Maldives | 22.32 (8.98 to 47.59) | 8 (5.34 to 12.16) | 19.83 (7.07 to 44.73) | 8.37 (4.84 to 13.66) |
| Mali | 1103.65 (447.77 to 2685.6) | 1228.08 (540.82 to 2667.88) | 22.97 (8.89 to 56.47) | 9.64 (3.89 to 21.51) |
| Malta | 9.68 (8.11 to 11.44) | 9.68 (7.45 to 12.43) | 11.59 (9.14 to 14.38) | 15.19 (11.12 to 20.16) |
| Marshall Islands | 2.8 (1.36 to 5.24) | 1.95 (0.92 to 3.65) | 13.17 (5.96 to 25.70) | 11.56 (5.12 to 23.12) |
| Mauritania | 191.37 (95.76 to 373.84) | 153.16 (56.31 to 281.61) | 18.52 (8.78 to 37.36) | 8.18 (2.86 to 15.94) |
| Mauritius | 3.75 (3.23 to 4.36) | 8.46 (6.87 to 10.4) | 1.19 (0.92 to 1.52) | 4.25 (3.04 to 6.03) |
| Mexico | 1173.07 (1045.87 to 1335.67) | 1742.43 (1341.8 to 2279.81) | 3.50 (3.07 to 4.02) | 5.75 (4.24 to 7.69) |
| Micronesia (Federated States of) | 7.15 (3.46 to 12.97) | 2.58 (1.32 to 4.59) | 15.87 (7.05 to 30.86) | 8.83 (4.05 to 17.36) |
| Monaco | 0.32 (0.17 to 0.52) | 0.16 (0.1 to 0.23) | 9.31 (4.45 to 17.00) | 3.23 (1.89 to 5.26) |
| Mongolia | 266.32 (165.49 to 399.25) | 69.92 (45.25 to 104.63) | 28.35 (14.96 to 46.36) | 6.42 (3.75 to 10.67) |
| Montenegro | 33.96 (22.44 to 49.24) | 5.56 (3.14 to 9.62) | 22.11 (13.49 to 34.33) | 5.06 (2.57 to 9.35) |
| Morocco | 2722.42 (468.71 to 9455.07) | 853.87 (125.43 to 2710.06) | 26.93 (4.47 to 97.67) | 9.08 (1.30 to 30.35) |
| Mozambique | 1195.56 (206.19 to 2914.23) | 672.72 (323.59 to 1216.56) | 17.51 (2.78 to 43.05) | 4.58 (2.03 to 8.84) |
| Myanmar | 3421.01 (1196.94 to 11329.89) | 2862.2 (1730.69 to 5092.22) | 23.55 (7.14 to 80.97) | 18.78 (9.42 to 36.09) |
| Namibia | 69.88 (43.56 to 102.4) | 63.5 (34.15 to 108.73) | 11.10 (6.32 to 18.23) | 7.86 (3.69 to 14.50) |
| Nauru | 0.68 (0.28 to 1.23) | 0.56 (0.29 to 0.93) | 15.29 (6.10 to 30.47) | 14.07 (6.45 to 26.33) |
| Nepal | 2118.99 (757.85 to 4726.87) | 1070.32 (653.38 to 1634.14) | 22.90 (7.65 to 52.53) | 11.92 (6.77 to 19.16) |
| Netherlands | 70.51 (61.3 to 81.08) | 78.86 (65.68 to 94.75) | 2.62 (2.16 to 3.19) | 3.06 (2.42 to 3.87) |
| New Zealand | 291.64 (263.01 to 323.49) | 149.46 (129.22 to 172.23) | 36.6 (32.4 to 41.49) | 16.14 (13.21 to 19.41) |
| Nicaragua | 183.12 (79.91 to 301.81) | 74.64 (44.08 to 118.37) | 9.76 (3.88 to 16.79) | 3.93 (2.13 to 6.66) |
| Niger | 1355.83 (455.83 to 3514.95) | 1563.13 (725.68 to 2922.69) | 28.94 (9.06 to 76.75) | 11.02 (4.79 to 21.31) |
| Nigeria | 13738.01 (7180.14 to 33195.08) | 13990.56 (8341.05 to 28405.69) | 31.04 (15.79 to 74.71) | 13.31 (7.77 to 27.28) |
| Niue | 0.1 (0.05 to 0.16) | 0.12 (0.06 to 0.21) | 13.24 (6.49 to 23.88) | 34.05 (16.00 to 62.75) |
| North Macedonia | 95.64 (48.72 to 175.35) | 9.61 (5.68 to 17.17) | 19.36 (8.94 to 37.68) | 3.08 (1.62 to 5.86) |
| Northern Mariana Islands | 0.73 (0.47 to 1.06) | 0.53 (0.34 to 0.76) | 5.54 (3.16 to 9.23) | 5.06 (2.86 to 8.03) |
| Norway | 90.58 (83.64 to 97.49) | 27.02 (22.83 to 32.63) | 11.45 (10.37 to 12.69) | 3.01 (2.40 to 3.76) |
| Oman | 217.56 (96.29 to 421.78) | 102.02 (63.34 to 154.25) | 23.84 (10.14 to 48.91) | 8.4 (4.83 to 13.63) |
| Pakistan | 14781.36 (6787.99 to 26304.65) | 20024.11 (11338.2 to 31201.59) | 28.27 (12.41 to 51.82) | 23.54 (12.86 to 39.31) |
| Palau | 0.57 (0.26 to 0.99) | 0.23 (0.12 to 0.35) | 13.18 (5.94 to 23.47) | 7.76 (3.93 to 13.49) |
| Palestine | 137.53 (69.66 to 246.67) | 123.15 (60.44 to 189.2) | 12.75 (5.76 to 24.85) | 6.88 (3.26 to 11.42) |
| Panama | 43.56 (35.43 to 54.11) | 61.56 (48.48 to 78.83) | 5.30 (4.05 to 6.89) | 5.61 (4.15 to 7.62) |
| Papua New Guinea | 531.98 (231.56 to 1059.73) | 1135.76 (485.36 to 2079.5) | 29.11 (12.41 to 60.29) | 26.72 (10.84 to 53.70) |
| Paraguay | 162.4 (100.97 to 223.72) | 83.12 (52.39 to 137.25) | 9.32 (5.55 to 13.74) | 4.36 (2.43 to 7.53) |
| Peru | 764.7 (306.18 to 1534.88) | 155.47 (93.64 to 250.69) | 9.17 (3.24 to 18.98) | 1.64 (0.85 to 2.95) |
| Philippines | 2775.75 (1363.2 to 4616.71) | 2244.33 (1643.45 to 3085.4) | 10.74 (5.10 to 18.82) | 6.74 (4.62 to 9.92) |
| Poland | 2600.97 (2422.06 to 2817.52) | 302.5 (252.33 to 368.11) | 30.44 (28.06 to 33.16) | 5.33 (4.26 to 6.81) |
| Portugal | 38.35 (33.44 to 44.49) | 23.97 (19.57 to 29.18) | 2.13 (1.76 to 2.58) | 1.85 (1.41 to 2.36) |
| Puerto Rico | 16.56 (14.1 to 19.79) | 8.72 (6.96 to 10.78) | 1.74 (1.36 to 2.27) | 2.54 (1.96 to 3.31) |
| Qatar | 14.25 (8.12 to 24.47) | 16.19 (10.41 to 27.02) | 10.32 (5.33 to 18.79) | 3.17 (1.85 to 5.43) |
| Republic of Korea | 2940.48 (1980.77 to 4146.58) | 544.21 (389.06 to 698.77) | 27.93 (16.81 to 42.21) | 9.90 (6.67 to 13.76) |
| Republic of Moldova | 80.46 (64.09 to 98.63) | 6.86 (5.18 to 9.06) | 6.53 (5.01 to 8.31) | 1.39 (0.97 to 1.94) |
| Romania | 3514.64 (2739.52 to 4152.31) | 649.54 (558.76 to 751.54) | 68.10 (51.00 to 83.71) | 23.04 (18.80 to 28.28) |
| Russian Federation | 3280.67 (3059.91 to 3533.99) | 979.7 (823.66 to 1144.48) | 9.66 (8.92 to 10.51) | 3.96 (3.26 to 4.69) |
| Rwanda | 1216.77 (355.7 to 2800.39) | 322.33 (178.02 to 516.39) | 32.14 (8.77 to 75.08) | 6.46 (3.32 to 11.12) |
| Saint Kitts and Nevis | 0.56 (0.44 to 0.78) | 0.45 (0.32 to 0.64) | 4.08 (2.98 to 6.17) | 4.79 (3.06 to 7.67) |
| Saint Lucia | 12.68 (10.27 to 15.4) | 4.32 (3.33 to 5.65) | 24.95 (19.22 to 32.07) | 15.46 (11.07 to 21.7) |
| Saint Vincent and the Grenadines | 6.16 (4.86 to 7.64) | 3.22 (2.58 to 4.11) | 16.24 (12.23 to 21.31) | 13.82 (10.12 to 18.85) |
| Samoa | 10.05 (5.37 to 16.67) | 7.02 (3.76 to 11.86) | 13.82 (6.81 to 24.62) | 8.56 (4.16 to 15.82) |
| San Marino | 0.35 (0.21 to 0.55) | 0.17 (0.11 to 0.24) | 9.56 (5.17 to 16.41) | 4.32 (2.65 to 6.70) |
| Sao Tome and Principe | 14.71 (6.55 to 30.59) | 6.26 (1.33 to 17.48) | 25.28 (10.31 to 53.84) | 8.61 (1.75 to 24.62) |
| Saudi Arabia | 2368.74 (1153.84 to 4187.06) | 366.23 (174.49 to 808.48) | 34.71 (16.18 to 63.62) | 5.04 (2.10 to 11.24) |
| Senegal | 1148.33 (612.33 to 2491.71) | 818.64 (292.15 to 1589.89) | 28.09 (13.63 to 61.33) | 12.66 (4.18 to 25.01) |
| Serbia | 261.79 (143.06 to 433.18) | 28.18 (13.71 to 59.84) | 13.04 (6.64 to 22.28) | 2.39 (1.04 to 5.36) |
| Seychelles | 3.8 (2.77 to 5.12) | 2.08 (1.56 to 2.72) | 16.19 (10.78 to 23.37) | 9.15 (6.16 to 12.88) |
| Sierra Leone | 941.97 (335.9 to 2476.25) | 746.2 (299.81 to 1646.08) | 43.64 (14.05 to 115.70) | 19.64 (7.45 to 44.48) |
| Singapore | 672.89 (605.35 to 749.07) | 165.62 (138.06 to 201.63) | 109.34 (94.23 to 126.56) | 20.43 (16.22 to 26.07) |
| Slovakia | 85.55 (64.66 to 113.5) | 29.56 (21.46 to 41.24) | 7.06 (5.01 to 9.68) | 3.56 (2.44 to 5.17) |
| Slovenia | 4.53 (3.93 to 5.19) | 1.84 (1.42 to 2.4) | 1.20 (0.98 to 1.46) | 0.60 (0.45 to 0.81) |
| Solomon Islands | 20.76 (9.38 to 37.16) | 22.85 (10.72 to 41.15) | 12.43 (5.34 to 24.41) | 8.53 (3.67 to 17.26) |
| Somalia | 1497.36 (429.67 to 3409.11) | 1444.57 (656.61 to 2708.48) | 34.27 (9.26 to 79.56) | 12.49 (5.32 to 24.75) |
| South Africa | 1971.65 (963.76 to 3019.55) | 826.92 (614.69 to 1211.34) | 14.27 (6.59 to 22.36) | 5.64 (3.99 to 8.55) |
| South Sudan | 1034.28 (169.9 to 2355.44) | 868.67 (259.04 to 2130.57) | 35.8 (5.72 to 82.58) | 19.56 (5.20 to 48.97) |
| Spain | 95.8 (84.25 to 107.74) | 169.47 (139.03 to 207.87) | 1.42 (1.18 to 1.70) | 2.90 (2.21 to 3.74) |
| Sri Lanka | 4860.91 (2838.52 to 6196.73) | 743.89 (507.97 to 1062.53) | 91.72 (52.68 to 126.05) | 15.07 (9.43 to 22.95) |
| Sudan | 3167.37 (396.69 to 14475.99) | 2166.54 (373.55 to 7234.05) | 32.34 (3.83 to 150.11) | 13.35 (2.22 to 46.42) |
| Suriname | 18.92 (9.38 to 29.38) | 13.37 (7.85 to 20.14) | 14.91 (6.43 to 25.44) | 10.05 (5.40 to 17.14) |
| Sweden | 354.93 (306.95 to 408.46) | 126.28 (103.73 to 157.46) | 22.54 (18.90 to 26.94) | 7.10 (5.55 to 9.42) |
| Switzerland | 25.51 (20.03 to 33.49) | 19.84 (16.3 to 23.84) | 2.22 (1.59 to 3.10) | 1.52 (1.18 to 1.94) |
| Syrian Arab Republic | 1684.67 (362.38 to 4875.2) | 273.92 (41.96 to 1145.72) | 27.52 (5.79 to 81.95) | 8.37 (1.25 to 34.65) |
| Taiwan (Province of China) | 365.88 (328.73 to 408.22) | 326.71 (281.96 to 375.5) | 7.25 (6.07 to 8.59) | 11.62 (9.25 to 14.37) |
| Tajikistan | 9 (4.06 to 15.75) | 11.13 (6.72 to 17.84) | 0.36 (0.16 to 0.67) | 0.30 (0.17 to 0.53) |
| Thailand | 1471.27 (899.51 to 2482.06) | 657.14 (441.95 to 856.46) | 9.21 (4.94 to 17.16) | 7.09 (4.28 to 10.22) |
| Timor-Leste | 72.07 (25.04 to 264.74) | 87.3 (48.97 to 161.18) | 19.01 (6.12 to 68.79) | 16.64 (8.35 to 33.48) |
| Togo | 458.95 (227.39 to 874.89) | 334.92 (148.96 to 586.68) | 23.85 (10.93 to 47.55) | 10.02 (4.15 to 18.27) |
| Tokelau | 0.06 (0.03 to 0.11) | 0.14 (0.06 to 0.26) | 11.56 (4.97 to 23.7) | 42.50 (17.55 to 84.68) |
| Tonga | 3.94 (2.16 to 6.19) | 2.55 (1.33 to 4.53) | 9.10 (4.69 to 15.43) | 6.32 (3.12 to 12.03) |
| Trinidad and Tobago | 120.93 (102.13 to 141.45) | 48.38 (37.85 to 62.1) | 30.69 (23.41 to 38.11) | 19.27 (14.45 to 25.86) |
| Tunisia | 498.15 (100.07 to 1700.51) | 107.71 (19.23 to 353.92) | 16.28 (3.17 to 57.19) | 4.10 (0.69 to 14.19) |
| Turkey | 1550.06 (561.5 to 3425.52) | 412.93 (204.95 to 651.37) | 7.77 (2.52 to 18.07) | 2.46 (1.13 to 4.19) |
| Turkmenistan | 254.86 (181.59 to 329.15) | 226.84 (184.55 to 282.22) | 15.82 (10.69 to 21.47) | 14.81 (10.98 to 19.65) |
| Tuvalu | 0.78 (0.34 to 1.41) | 0.34 (0.19 to 0.56) | 18.90 (7.77 to 36.14) | 9.07 (4.41 to 16.71) |
| Uganda | 2860.16 (710.77 to 6525.73) | 1651.3 (838.05 to 3105.72) | 28.35 (6.51 to 65.41) | 7.98 (3.73 to 15.72) |
| Ukraine | 550.36 (438.82 to 672.32) | 261.51 (211.41 to 322.6) | 4.92 (3.67 to 6.36) | 4.54 (3.40 to 5.90) |
| United Arab Emirates | 135.66 (67.4 to 254.12) | 73.62 (47.95 to 124.39) | 21.54 (10.30 to 41.40) | 5.76 (3.43 to 10.28) |
| United Kingdom | 2679.42 (2437.92 to 2992.75) | 814.71 (695.65 to 949.85) | 24.47 (22.12 to 27.48) | 7.27 (6.09 to 8.63) |
| United Republic of Tanzania | 3135.65 (818.77 to 6651.63) | 1854.75 (1017.01 to 3027.21) | 23.15 (5.74 to 50.28) | 7.39 (3.61 to 12.95) |
| United States of America | 15927.04 (14864.28 to 17001.03) | 9693.55 (8641.61 to 10888.83) | 28.33 (26.21 to 30.6) | 17.39 (15.18 to 19.91) |
| United States Virgin Islands | 7.53 (5.17 to 11.05) | 1.19 (0.75 to 1.95) | 23.69 (14.34 to 37.68) | 9.99 (5.80 to 17.38) |
| Uruguay | 220.4 (192.25 to 255.04) | 31.38 (23.85 to 40.21) | 28.06 (23.65 to 33.49) | 5.32 (3.82 to 7.19) |
| Uzbekistan | 854.78 (579.94 to 1105.02) | 419.05 (318.09 to 544.08) | 9.37 (5.90 to 12.85) | 3.98 (2.83 to 5.52) |
| Vanuatu | 11.93 (6.4 to 19.67) | 14.16 (7.52 to 24.35) | 15.94 (7.82 to 29.12) | 11.95 (5.71 to 22.92) |
| Venezuela (Bolivarian Republic of) | 687.99 (592.35 to 848.3) | 439.23 (322.82 to 591.79) | 9.58 (7.72 to 12.35) | 6.84 (4.67 to 9.72) |
| Viet Nam | 2463.24 (1499.96 to 4055.07) | 1451.27 (810.11 to 2639.18) | 9.20 (5.14 to 16.15) | 6.02 (3.09 to 11.53) |
| Yemen | 1979.38 (281.23 to 8715.48) | 1887.84 (318.94 to 6257.04) | 25.28 (3.48 to 113.38) | 13.99 (2.33 to 47.73) |
| Zambia | 941.73 (214.59 to 2377.5) | 419.91 (222.72 to 688.61) | 22.11 (4.89 to 56.6) | 5.04 (2.33 to 9.03) |
| Zimbabwe | 528.1 (347.03 to 795.36) | 1003.06 (574.07 to 1553.85) | 10.64 (6.26 to 17.50) | 15.91 (8.28 to 27.14) |

ASR: age-standardized rate**,** DALY: disability-adjusted life years, SDI: sociodemographic index, UI: uncertain interval.

*Number of cases for countries or territories is actual data not divided by 1000.

**Table S5: Estimated average percent change of ASR of incidence, prevalence, mortality and DALYs for childhood myocarditis from 1990 to 2021 by countries/territories**

| **Country or territories** | **EAPC, % (95% CI)** | | | |
| --- | --- | --- | --- | --- |
|  | **Incidence** | **Prevalence** | **Mortality** | **DALYs** |
| Afghanistan | -0.02 (-0.02 to -0.02) | -0.18 (-0.2 to -0.15) | -1.44 (-1.56 to -1.32) | -1.45 (-1.57 to -1.33) |
| Albania | 0.01 (0 to 0.01) | 0.47 (0.25 to 0.69) | -1.71 (-2.01 to -1.42) | -1.67 (-1.95 to -1.38) |
| Algeria | -0.01 (-0.01 to -0.01) | 0.01 (-0.02 to 0.04) | -3.44 (-3.61 to -3.27) | -3.40 (-3.57 to -3.23) |
| American Samoa | -0.01 (-0.01 to -0.01) | 0.09 (0.08 to 0.10) | -1.44 (-1.72 to -1.16) | -1.41 (-1.68 to -1.14) |
| Andorra | -0.02 (-0.03 to -0.01) | -0.69 (-0.75 to -0.62) | -4.93 (-5.16 to -4.71) | -4.45 (-4.65 to -4.24) |
| Angola | 0 | -1.87 (-1.95 to -1.79) | -3.39 (-3.65 to -3.14) | -3.41 (-3.67 to -3.16) |
| Antigua and Barbuda | 0.01 (0.01 to 0.01) | -0.24 (-0.34 to -0.14) | -1.16 (-1.29 to -1.03) | -1.08 (-1.21 to -0.96) |
| Argentina | 0 | -1.35 (-1.60 to -1.10) | -5.93 (-6.39 to -5.47) | -5.82 (-6.27 to -5.37) |
| Armenia | 0.05 (0.04 to 0.06) | -1.54 (-1.90 to -1.17) | -4.80 (-6.10 to -3.49) | -4.54 (-5.74 to -3.32) |
| Australia | 0 | -0.93 (-1.86 to 0.01) | -4.69 (-5.32 to -4.05) | -4.17 (-4.80 to -3.53) |
| Austria | 0.13 (-0.04 to 0.30) | 4.35 (2.59 to 6.15) | 2.17 (-0.21 to 4.61) | 2.65 (0.33 to 5.03) |
| Azerbaijan | 0.04 (0.04 to 0.05) | 0 (-0.05 to 0.05) | -1.94 (-2.49 to -1.39) | -1.90 (-2.42 to -1.37) |
| Bahamas | 0 | 0.25 (0.04 to 0.46) | -1.79 (-2.19 to -1.39) | -1.66 (-2.05 to -1.27) |
| Bahrain | 0.01 (0 to 0.02) | 0.14 (0.03 to 0.24) | -4.15 (-4.35 to -3.94) | -3.95 (-4.16 to -3.74) |
| Bangladesh | -0.02 (-0.02 to -0.01) | 0.05 (-0.01 to 0.11) | -1.25 (-1.41 to -1.09) | -1.26 (-1.42 to -1.10) |
| Barbados | 0.01 (0.01 to 0.01) | 1.18 (1.04 to 1.33) | 0.05 (-0.25 to 0.34) | 0.14 (-0.14 to 0.41) |
| Belarus | 0 | -1.38 (-1.66 to -1.10) | -6.01 (-6.96 to -5.05) | -5.37 (-6.22 to -4.52) |
| Belgium | -0.01 (-0.02 to 0) | 4.11 (2.39 to 5.85) | 0.53 (-1.50 to 2.60) | 1.22 (-0.76 to 3.23) |
| Belize | 0 | -0.60 (-0.89 to -0.31) | -3.80 (-4.31 to -3.29) | -3.69 (-4.19 to -3.20) |
| Benin | -0.02 (-0.02 to -0.02) | -0.45 (-0.51 to -0.39) | -2.60 (-2.80 to -2.40) | -2.58 (-2.77 to -2.38) |
| Bermuda | 0.01 (0 to 0.01) | 1.29 (1.10 to 1.49) | -0.62 (-1.23 to -0.01) | -0.37 (-0.93 to 0.19) |
| Bhutan | -0.01 (-0.02 to -0.01) | -0.43 (-0.51 to -0.36) | -1.49 (-1.95 to -1.02) | -1.51 (-1.96 to -1.06) |
| Bolivia (Plurinational State of) | 0 | -0.63 (-0.68 to -0.59) | -3.47 (-3.55 to -3.38) | -3.42 (-3.50 to -3.34) |
| Bosnia and Herzegovina | 0 (0 to 0.01) | -0.09 (-0.15 to -0.02) | -2.96 (-3.3 to -2.61) | -2.90 (-3.24 to -2.55) |
| Botswana | 0.01 (0.01 to 0.01) | 0.16 (-0.01 to 0.33) | -0.79 (-0.90 to -0.68) | -0.75 (-0.86 to -0.64) |
| Brazil | 0 | 0.39 (0.21 to 0.57) | -2.02 (-2.64 to -1.40) | -1.97 (-2.57 to -1.36) |
| Brunei Darussalam | 0.02 (0.01 to 0.03) | 0.11 (-0.01 to 0.23) | -1.71 (-1.88 to -1.54) | -1.57 (-1.73 to -1.41) |
| Bulgaria | 0 | 1.42 (1.15 to 1.69) | -2.23 (-3.09 to -1.36) | -2.08 (-2.91 to -1.24) |
| Burkina Faso | -0.03 (-0.03 to -0.03) | -0.41 (-0.48 to -0.35) | -2.07 (-2.31 to -1.83) | -2.05 (-2.29 to -1.82) |
| Burundi | 0 (-0.01 to 0) | -2.25 (-2.36 to -2.14) | -4.06 (-4.44 to -3.68) | -4.04 (-4.42 to -3.67) |
| Cabo Verde | 0 | -1.43 (-1.52 to -1.33) | -2.73 (-2.86 to -2.61) | -2.69 (-2.82 to -2.57) |
| Cambodia | 0.01 (0.01 to 0.01) | 0.38 (0.35 to 0.42) | -1.11 (-1.19 to -1.03) | -1.10 (-1.18 to -1.03) |
| Cameroon | 0 | -0.63 (-0.66 to -0.61) | -1.93 (-2.16 to -1.69) | -1.91 (-2.14 to -1.68) |
| Canada | 0 | 2.40 (1.61 to 3.20) | -1.63 (-2.33 to -0.92) | -0.91 (-1.59 to -0.22) |
| Central African Republic | 0 | -1.06 (-1.11 to -1.00) | -1.64 (-1.78 to -1.51) | -1.67 (-1.8 to -1.54) |
| Chad | 0 | -0.27 (-0.31 to -0.23) | -2.07 (-2.26 to -1.88) | -2.06 (-2.24 to -1.87) |
| Chile | -0.18 (-0.22 to -0.13) | -0.04 (-0.47 to 0.38) | -4.49 (-5.16 to -3.81) | -4.21 (-4.86 to -3.55) |
| China | -0.97 (-1.13 to -0.81) | 0.97 (0.83 to 1.10) | -4.64 (-5.03 to -4.25) | -4.61 (-4.99 to -4.22) |
| Colombia | 0.01 (0 to 0.01) | 2.02 (1.76 to 2.28) | 1.47 (1.01 to 1.93) | 1.48 (1.03 to 1.94) |
| Comoros | 0 | -1.58 (-1.63 to -1.54) | -4.45 (-4.60 to -4.31) | -4.43 (-4.57 to -4.29) |
| Congo | 0.01 (0.01 to 0.01) | -1.20 (-1.29 to -1.11) | -3.76 (-4.00 to -3.51) | -3.78 (-4.02 to -3.53) |
| Cook Islands | -0.01 (-0.02 to -0.01) | -0.02 (-0.03 to -0.02) | -5.28 (-5.84 to -4.71) | -3.91 (-4.30 to -3.52) |
| Costa Rica | 0 | 0.28 (0.04 to 0.52) | -1.39 (-1.73 to -1.05) | -1.30 (-1.63 to -0.97) |
| Coted'Ivoire | 0.01 (0 to 0.01) | -0.32 (-0.37 to -0.27) | -2.32 (-2.61 to -2.03) | -2.30 (-2.59 to -2.01) |
| Croatia | -0.07 (-0.26 to 0.12) | 1.29 (0.91 to 1.66) | -3.04 (-3.36 to -2.72) | -2.56 (-2.85 to -2.28) |
| Cuba | 0 | 1.37 (1.00 to 1.73) | -1.28 (-1.70 to -0.86) | -1.12 (-1.53 to -0.71) |
| Cyprus | 0.04 (0.02 to 0.07) | -0.75 (-0.93 to -0.58) | -6.38 (-6.94 to -5.81) | -5.59 (-6.06 to -5.12) |
| Czechia | -0.09 (-0.15 to -0.03) | 2.05 (1.90 to 2.21) | -3.34 (-3.69 to -2.99) | -2.12 (-2.42 to -1.81) |
| Democratic People's Republic of Korea | 0 | 0.23 (0.15 to 0.32) | -2.03 (-2.33 to -1.73) | -2.04 (-2.34 to -1.75) |
| Democratic Republic of the Congo | 0 | -1.24 (-1.34 to -1.14) | -3.14 (-3.37 to -2.91) | -3.16 (-3.39 to -2.93) |
| Denmark | 0 (-0.04 to 0.05) | 3.35 (1.96 to 4.77) | -1.71 (-2.92 to -0.49) | -0.93 (-2.14 to 0.28) |
| Djibouti | 0.02 (0.02 to 0.03) | -1.96 (-2.07 to -1.86) | -4.25 (-4.64 to -3.87) | -4.24 (-4.62 to -3.86) |
| Dominica | 0 | -0.39 (-0.50 to -0.28) | 0.54 (0.15 to 0.92) | 0.52 (0.14 to 0.90) |
| Dominican Republic | 0.01 (0.01 to 0.01) | 0.19 (0.07 to 0.31) | -1.35 (-1.55 to -1.15) | -1.32 (-1.52 to -1.13) |
| Ecuador | 0.09 (0.07 to 0.12) | -2.11 (-2.48 to -1.74) | -5.46 (-6.31 to -4.60) | -5.34 (-6.16 to -4.50) |
| Egypt | 0 (0 to 0.01) | -1.80 (-1.87 to -1.73) | -5.44 (-5.60 to -5.27) | -3.95 (-4.04 to -3.86) |
| El Salvador | 0.01 (0.01 to 0.01) | -0.22 (-0.26 to -0.18) | -2.99 (-3.37 to -2.61) | -2.95 (-3.31 to -2.58) |
| Equatorial Guinea | 0.06 (0.05 to 0.06) | -1.60 (-1.71 to -1.49) | -5.46 (-5.83 to -5.10) | -5.43 (-5.79 to -5.07) |
| Eritrea | 0 | -2.19 (-2.30 to -2.07) | -3.4 (-3.53 to -3.26) | -3.39 (-3.52 to -3.26) |
| Estonia | 0 | -1.62 (-1.87 to -1.37) | -10.94 (-12.07 to -9.81) | -8.59 (-9.36 to -7.82) |
| Eswatini | 0.02 (0.02 to 0.03) | -0.76 (-0.99 to -0.54) | -1.10 (-1.17 to -1.03) | -1.11 (-1.18 to -1.03) |
| Ethiopia | 0 | -3.29 (-3.47 to -3.12) | -4.99 (-5.22 to -4.75) | -4.97 (-5.20 to -4.74) |
| Fiji | 0 | 0.28 (0.26 to 0.31) | 1.10 (0.83 to 1.37) | 1.08 (0.81 to 1.35) |
| Finland | 0.13 (0.05 to 0.21) | 3.19 (1.86 to 4.53) | -2.40 (-3.64 to -1.14) | -1.51 (-2.71 to -0.30) |
| France | 0 | 4.38 (3.04 to 5.74) | 2.97 (0.99 to 4.98) | 3.61 (1.75 to 5.51) |
| Gabon | -0.01 (-0.01 to -0.01) | -1.00 (-1.05 to -0.96) | -2.66 (-2.89 to -2.43) | -2.67 (-2.89 to -2.44) |
| Gambia | 0 | -0.81 (-0.84 to -0.79) | -2.59 (-2.90 to -2.27) | -2.57 (-2.88 to -2.26) |
| Georgia | 0.02 (0.02 to 0.03) | -1.60 (-2.51 to -0.69) | -5.19 (-7.25 to -3.08) | -4.89 (-6.84 to -2.9) |
| Germany | -0.11 (-0.20 to -0.03) | 3.70 (2.14 to 5.28) | 0.41 (-1.38 to 2.23) | 0.94 (-0.82 to 2.74) |
| Ghana | -0.01 (-0.01 to -0.01) | -1.14 (-1.25 to -1.04) | -2.37 (-2.55 to -2.18) | -2.36 (-2.54 to -2.18) |
| Greece | 0.02 (0.02 to 0.02) | 3.68 (2.33 to 5.04) | 3.28 (1.13 to 5.48) | 3.49 (1.41 to 5.61) |
| Greenland | -0.01 (-0.02 to 0) | 2.13 (1.78 to 2.49) | -1.84 (-2.09 to -1.6) | -1.67 (-1.90 to -1.43) |
| Grenada | 0.02 (0.01 to 0.02) | -0.11 (-0.30 to 0.08) | -1.21 (-1.42 to -1.00) | -1.17 (-1.37 to -0.97) |
| Guam | 0.02 (0.01 to 0.02) | -0.19 (-0.41 to 0.03) | -0.25 (-0.66 to 0.17) | -0.22 (-0.60 to 0.17) |
| Guatemala | 0.01 (0.01 to 0.01) | -0.07 (-0.33 to 0.19) | 0.91 (0.46 to 1.37) | 0.90 (0.45 to 1.34) |
| Guinea | -0.02 (-0.02 to -0.01) | -0.59 (-0.62 to -0.56) | -2.47 (-2.61 to -2.34) | -2.46 (-2.59 to -2.33) |
| Guinea-Bissau | -0.01 (-0.01 to -0.01) | -0.87 (-0.91 to -0.82) | -2.95 (-3.20 to -2.70) | -2.94 (-3.19 to -2.69) |
| Guyana | 0.01 (0 to 0.03) | 0.81 (0.63 to 0.99) | 0.42 (0.01 to 0.84) | 0.40 (0 to 0.80) |
| Haiti | 0 (-0.01 to 0) | 0.26 (0.20 to 0.32) | -0.21 (-0.34 to -0.08) | -0.21 (-0.34 to -0.08) |
| Honduras | 0 | -0.30 (-0.34 to -0.26) | -1.96 (-2.09 to -1.83) | -1.90 (-2.03 to -1.78) |
| Hungary | 0 | 1.24 (1.05 to 1.43) | -3.57 (-3.92 to -3.21) | -3.11 (-3.41 to -2.81) |
| Iceland | 0 (-0.01 to 0) | 3.27 (1.92 to 4.63) | -0.35 (-1.90 to 1.22) | 0.15 (-1.36 to 1.69) |
| India | 0 | -0.57 (-0.62 to -0.52) | -1.29 (-1.46 to -1.11) | -1.28 (-1.45 to -1.11) |
| Indonesia | 0 | 0.04 (-0.03 to 0.10) | -0.52 (-0.63 to -0.42) | -0.54 (-0.63 to -0.44) |
| Iran (Islamic Republic of) | -0.01 (-0.01 to 0) | -0.22 (-0.25 to -0.18) | -3.59 (-4.51 to -2.67) | -3.54 (-4.43 to -2.64) |
| Iraq | -0.21 (-0.22 to -0.21) | 0.02 (-0.04 to 0.08) | -2.58 (-2.78 to -2.38) | -2.54 (-2.74 to -2.34) |
| Ireland | 0 (-0.01 to 0) | 5.59 (3.92 to 7.28) | 0.74 (-1.29 to 2.81) | 1.57 (-0.39 to 3.57) |
| Israel | 0 | 2.42 (0.96 to 3.89) | -1.23 (-3.21 to 0.8) | -0.66 (-2.57 to 1.29) |
| Italy | -0.92 (-1.14 to -0.7) | -2.44 (-3.11 to -1.76) | -9.57 (-10.71 to -8.42) | -8.55 (-9.56 to -7.54) |
| Jamaica | 0 (0 to 0.01) | 1.22 (1.12 to 1.31) | -0.75 (-1.19 to -0.31) | -0.62 (-1.04 to -0.20) |
| Japan | 0.34 (0.3 to 0.38) | 2.46 (1.75 to 3.18) | -1.76 (-2.7 to -0.81) | -1.22 (-2.12 to -0.32) |
| Jordan | 0 | 0.17 (0.10 to 0.25) | -3.29 (-3.52 to -3.05) | -3.17 (-3.39 to -2.95) |
| Kazakhstan | 0.01 (0.01 to 0.01) | 3.13 (2.98 to 3.27) | 6.08 (4.41 to 7.77) | 5.86 (4.29 to 7.45) |
| Kenya | 0.01 (0.01 to 0.01) | -1.06 (-1.12 to -1.00) | -2.93 (-3.1 to -2.75) | -2.91 (-3.07 to -2.74) |
| Kiribati | -0.02 (-0.02 to -0.01) | 0.11 (0.08 to 0.14) | -0.52 (-0.59 to -0.45) | -0.56 (-0.63 to -0.49) |
| Kuwait | 0 (-0.01 to 0) | 1.86 (1.29 to 2.43) | -1.45 (-2.07 to -0.84) | -1.31 (-1.91 to -0.69) |
| Kyrgyzstan | 0.01 (0 to 0.01) | 1.21 (0.59 to 1.83) | -1.34 (-2.55 to -0.10) | -1.29 (-2.48 to -0.08) |
| Lao People's Democratic Republic | 0 | 0.14 (0.12 to 0.15) | -0.81 (-0.89 to -0.73) | -0.84 (-0.91 to -0.76) |
| Latvia | -0.12 (-0.23 to -0.01) | -1.21 (-1.35 to -1.07) | -9.53 (-10.64 to -8.40) | -7.58 (-8.37 to -6.79) |
| Lebanon | -0.01 (-0.01 to -0.01) | 0.39 (0.30 to 0.48) | -2.68 (-2.97 to -2.40) | -2.52 (-2.78 to -2.26) |
| Lesotho | 0 | -0.28 (-0.37 to -0.19) | -0.12 (-0.29 to 0.05) | -0.15 (-0.32 to 0.02) |
| Liberia | -0.02 (-0.02 to -0.01) | -0.76 (-0.79 to -0.72) | -3.71 (-4.00 to -3.41) | -3.68 (-3.97 to -3.39) |
| Libya | -0.06 (-0.07 to -0.05) | 0.31 (0.25 to 0.38) | -2.28 (-2.49 to -2.07) | -2.28 (-2.48 to -2.07) |
| Lithuania | -0.09 (-0.14 to -0.05) | -0.97 (-1.23 to -0.70) | -8.69 (-9.89 to -7.48) | -6.77 (-7.64 to -5.90) |
| Luxembourg | -0.1 (-0.16 to -0.04) | 4.23 (3.23 to 5.24) | -1.06 (-1.97 to -0.13) | 0.30 (-0.57 to 1.18) |
| Madagascar | -0.01 (-0.01 to 0) | -1.80 (-1.86 to -1.75) | -3.28 (-3.38 to -3.18) | -3.29 (-3.39 to -3.19) |
| Malawi | 0 | -2.55 (-2.69 to -2.41) | -4.67 (-4.87 to -4.47) | -4.67 (-4.86 to -4.48) |
| Malaysia | 0 | -0.13 (-0.23 to -0.02) | -2.82 (-3.17 to -2.47) | -2.68 (-3.02 to -2.34) |
| Maldives | 0.01 (0.01 to 0.01) | -0.08 (-0.17 to 0.01) | -2.78 (-2.92 to -2.65) | -2.70 (-2.83 to -2.56) |
| Mali | 0 | -0.50 (-0.54 to -0.46) | -2.99 (-3.16 to -2.82) | -2.96 (-3.13 to -2.80) |
| Malta | 0.01 (0.01 to 0.01) | 4.15 (2.59 to 5.73) | 0.28 (-1.02 to 1.60) | 0.70 (-0.61 to 2.02) |
| Marshall Islands | -0.02 (-0.02 to -0.01) | -0.02 (-0.04 to 0) | -0.53 (-0.72 to -0.34) | -0.57 (-0.76 to -0.39) |
| Mauritania | -0.01 (-0.01 to -0.01) | -0.62 (-0.65 to -0.60) | -3.17 (-3.48 to -2.85) | -3.12 (-3.44 to -2.81) |
| Mauritius | 0 | 1.87 (1.45 to 2.29) | 7.36 (4.29 to 10.52) | 6.57 (3.88 to 9.33) |
| Mexico | 0 | 1.55 (1.36 to 1.74) | 3.33 (2.59 to 4.06) | 3.22 (2.51 to 3.93) |
| Micronesia (Federated States of) | -0.03 (-0.03 to -0.03) | -0.06 (-0.06 to -0.05) | -1.98 (-2.09 to -1.88) | -2.02 (-2.12 to -1.91) |
| Monaco | -0.04 (-0.04 to -0.03) | -1.11 (-1.21 to -1.02) | -4.80 (-5.47 to -4.12) | -4.44 (-5.06 to -3.82) |
| Mongolia | 0.01 (0.01 to 0.01) | -1.21 (-1.47 to -0.95) | -4.82 (-5.46 to -4.18) | -4.73 (-5.34 to -4.12) |
| Montenegro | 0.01 (0.01 to 0.01) | -1.52 (-1.69 to -1.35) | -5.02 (-5.47 to -4.58) | -4.83 (-5.23 to -4.42) |
| Morocco | -0.03 (-0.03 to -0.03) | -0.05 (-0.08 to -0.01) | -3.36 (-3.65 to -3.07) | -3.33 (-3.61 to -3.05) |
| Mozambique | 0 | -2.09 (-2.23 to -1.94) | -4.18 (-4.30 to -4.05) | -4.16 (-4.28 to -4.04) |
| Myanmar | 0.01 (0.01 to 0.01) | 0.36 (0.33 to 0.39) | -0.76 (-0.91 to -0.62) | -0.78 (-0.92 to -0.64) |
| Namibia | 0 | -0.40 (-0.57 to -0.23) | -0.90 (-1.09 to -0.72) | -0.89 (-1.08 to -0.70) |
| Nauru | 0 | -0.04 (-0.06 to -0.01) | -0.38 (-0.69 to -0.06) | -0.40 (-0.71 to -0.09) |
| Nepal | 0 | -0.60 (-0.63 to -0.57) | -1.77 (-2.09 to -1.45) | -1.77 (-2.08 to -1.46) |
| Netherlands | 0 | 4.17 (2.44 to 5.93) | 0.18 (-1.88 to 2.29) | 0.91 (-1.10 to 2.97) |
| New Zealand | -0.48 (-0.61 to -0.35) | 0.96 (-0.07 to 2.00) | -2.70 (-3.30 to -2.09) | -2.30 (-2.92 to -1.68) |
| Nicaragua | 0.01 (0.01 to 0.01) | -0.34 (-0.45 to -0.23) | -2.69 (-2.92 to -2.47) | -2.64 (-2.85 to -2.42) |
| Niger | 0 | -0.58 (-0.62 to -0.55) | -3.61 (-3.96 to -3.26) | -3.58 (-3.92 to -3.23) |
| Nigeria | 0 (-0.01 to 0) | -0.87 (-0.91 to -0.83) | -3.04 (-3.34 to -2.74) | -2.99 (-3.28 to -2.70) |
| Niue | -0.01 (-0.02 to 0.01) | 0.04 (0.02 to 0.06) | 0.33 (-0.67 to 1.33) | 0.29 (-0.68 to 1.28) |
| North Macedonia | 0 | -0.14 (-0.27 to -0.01) | -5.51 (-5.77 to -5.24) | -5.28 (-5.54 to -5.03) |
| Northern Mariana Islands | 0.01 (0 to 0.02) | 1.02 (0.93 to 1.12) | 0.5 (-0.03 to 1.02) | 0.52 (0.02 to 1.01) |
| Norway | -0.26 (-0.45 to -0.07) | 0.67 (0.05 to 1.29) | -4.33 (-5.07 to -3.59) | -3.43 (-4.09 to -2.75) |
| Oman | -0.01 (-0.01 to -0.01) | -0.29 (-0.58 to 0) | -2.44 (-3.05 to -1.83) | -2.38 (-2.99 to -1.77) |
| Pakistan | 0 | -0.42 (-0.49 to -0.35) | 0.62 (0.19 to 1.05) | 0.58 (0.15 to 1.01) |
| Palau | 0 (-0.01 to 0) | 0.04 (-0.02 to 0.10) | -1.65 (-1.76 to -1.53) | -1.62 (-1.74 to -1.51) |
| Palestine | -0.01 (-0.01 to -0.01) | 0.19 (0.08 to 0.29) | -1.91 (-2.05 to -1.77) | -1.88 (-2.01 to -1.74) |
| Panama | 0 | 0.95 (0.75 to 1.15) | -0.04 (-0.42 to 0.34) | 0 (-0.37 to 0.37) |
| Papua New Guinea | 0 (-0.01 to 0) | -0.08 (-0.09 to -0.07) | -0.16 (-0.2 to -0.11) | -0.18 (-0.22 to -0.13) |
| Paraguay | 0 | -0.54 (-0.62 to -0.47) | -2.76 (-3.02 to -2.51) | -2.71 (-2.96 to -2.46) |
| Peru | 0.01 (0.01 to 0.02) | -1.06 (-1.19 to -0.92) | -5.78 (-6.00 to -5.56) | -5.60 (-5.82 to -5.38) |
| Philippines | -0.01 (-0.01 to 0) | -0.16 (-0.23 to -0.10) | -1.13 (-1.28 to -0.98) | -1.15 (-1.29 to -1.02) |
| Poland | -0.05 (-0.1 to -0.01) | -0.57 (-0.86 to -0.29) | -5.94 (-6.19 to -5.69) | -5.45 (-5.66 to -5.25) |
| Portugal | 0 (0 to 0.01) | 3.46 (2.15 to 4.80) | -0.90 (-2.64 to 0.87) | -0.16 (-1.82 to 1.52) |
| Puerto Rico | 0 | 2.02 (1.19 to 2.87) | 0.58 (-0.95 to 2.12) | 0.88 (-0.56 to 2.35) |
| Qatar | -0.01 (-0.02 to -0.01) | 0.58 (0.42 to 0.74) | -3.89 (-4.13 to -3.65) | -3.67 (-3.89 to -3.45) |
| Republic of Korea | -0.01 (-0.02 to -0.01) | 1.27 (1.09 to 1.45) | -3.58 (-3.72 to -3.43) | -3.10 (-3.24 to -2.96) |
| Republic of Moldova | 0 | -1.14 (-1.41 to -0.87) | -5.87 (-6.80 to -4.94) | -5.44 (-6.28 to -4.59) |
| Romania | -0.13 (-0.18 to -0.07) | 1.35 (1.19 to 1.50) | -3.87 (-4.28 to -3.46) | -3.64 (-4.04 to -3.24) |
| Russian Federation | 0 | -1.67 (-1.91 to -1.44) | -3.50 (-4.49 to -2.50) | -3.38 (-4.28 to -2.46) |
| Rwanda | 0.01 (0.01 to 0.01) | -2.65 (-2.88 to -2.43) | -5.99 (-6.38 to -5.58) | -5.94 (-6.34 to -5.55) |
| Saint Kitts and Nevis | 0 | 0.54 (0.06 to 1.01) | 0.90 (0.54 to 1.26) | 0.87 (0.50 to 1.24) |
| Saint Lucia | 0.02 (0.01 to 0.02) | 0.21 (0.03 to 0.38) | -1.39 (-1.60 to -1.19) | -1.34 (-1.54 to -1.14) |
| Saint Vincent and the Grenadines | 0.01 (0 to 0.01) | 1.14 (0.64 to 1.64) | -0.91 (-1.32 to -0.49) | -0.88 (-1.30 to -0.46) |
| Samoa | -0.03 (-0.03 to -0.02) | -0.10 (-0.11 to -0.09) | -1.50 (-1.62 to -1.39) | -1.51 (-1.62 to -1.39) |
| San Marino | 0.01 (0.01 to 0.01) | 0.99 (0.60 to 1.37) | -2.60 (-2.79 to -2.4) | -1.96 (-2.15 to -1.76) |
| Sao Tome and Principe | -0.01 (-0.01 to -0.01) | -0.73 (-0.80 to -0.67) | -3.44 (-3.91 to -2.97) | -3.41 (-3.87 to -2.95) |
| Saudi Arabia | -0.02 (-0.03 to -0.01) | -1.30 (-1.43 to -1.17) | -6.95 (-7.38 to -6.51) | -6.88 (-7.31 to -6.45) |
| Senegal | 0.02 (0.02 to 0.02) | -0.67 (-0.70 to -0.63) | -2.53 (-2.78 to -2.28) | -2.52 (-2.76 to -2.27) |
| Serbia | -0.1 (-0.2 to -0.01) | -1.00 (-1.10 to -0.91) | -6.46 (-7.08 to -5.83) | -6.14 (-6.73 to -5.53) |
| Seychelles | 0 | -0.36 (-0.60 to -0.12) | -1.11 (-1.47 to -0.75) | -1.04 (-1.37 to -0.71) |
| Sierra Leone | -0.06 (-0.07 to -0.05) | -0.63 (-0.70 to -0.56) | -2.95 (-3.18 to -2.72) | -2.94 (-3.17 to -2.71) |
| Singapore | -0.02 (-0.03 to 0) | 0.64 (0.32 to 0.95) | -5.38 (-5.72 to -5.03) | -4.73 (-5.04 to -4.42) |
| Slovakia | -0.12 (-0.19 to -0.06) | 0.35 (0.30 to 0.41) | -2.11 (-2.36 to -1.85) | -1.88 (-2.11 to -1.65) |
| Slovenia | -0.07 (-0.16 to 0.03) | 0.95 (0.80 to 1.11) | -4.25 (-4.77 to -3.73) | -2.85 (-3.24 to -2.46) |
| Solomon Islands | -0.01 (-0.01 to -0.01) | -0.07 (-0.08 to -0.06) | -1.21 (-1.38 to -1.05) | -1.24 (-1.41 to -1.08) |
| Somalia | -0.02 (-0.02 to -0.02) | -1.78 (-1.85 to -1.70) | -3.01 (-3.28 to -2.75) | -3.00 (-3.26 to -2.74) |
| South Africa | 0.01 (0.01 to 0.01) | -1.22 (-1.46 to -0.99) | -3.09 (-3.24 to -2.94) | -3.04 (-3.19 to -2.90) |
| South Sudan | 0 | -1.50 (-1.75 to -1.24) | -1.90 (-2.51 to -1.29) | -1.91 (-2.51 to -1.30) |
| Spain | -0.25 (-0.30 to -0.20) | 3.78 (2.44 to 5.13) | 2.03 (-0.04 to 4.15) | 2.43 (0.43 to 4.47) |
| Sri Lanka | -0.04 (-0.05 to -0.02) | -2.79 (-3.06 to -2.52) | -7.37 (-8.18 to -6.55) | -7.27 (-8.07 to -6.46) |
| Sudan | -0.04 (-0.04 to -0.04) | -0.48 (-0.50 to -0.47) | -2.55 (-2.74 to -2.36) | -2.56 (-2.75 to -2.37) |
| Suriname | 0.02 (0.01 to 0.02) | 0.06 (-0.03 to 0.14) | -1.12 (-1.29 to -0.96) | -1.11 (-1.26 to -0.95) |
| Sweden | -0.07 (-0.14 to 0.01) | -0.78 (-1.16 to -0.40) | -4.31 (-4.97 to -3.65) | -3.50 (-4.03 to -2.98) |
| Switzerland | 0.03 (0.01 to 0.04) | 2.69 (1.29 to 4.10) | -1.77 (-3.48 to -0.03) | -1.01 (-2.68 to 0.68) |
| Syrian Arab Republic | -0.04 (-0.04 to -0.03) | -0.36 (-0.44 to -0.27) | -4.14 (-4.76 to -3.52) | -4.16 (-4.77 to -3.55) |
| Taiwan (Province of China) | 0.47 (0.35 to 0.59) | 5.47 (4.83 to 6.11) | 4.28 (3.27 to 5.30) | 4.41 (3.42 to 5.40) |
| Tajikistan | 0.02 (0.02 to 0.02) | -0.14 (-0.17 to -0.12) | -0.85 (-1.10 to -0.60) | -0.67 (-0.83 to -0.50) |
| Thailand | 0 | 0.92 (0.83 to 1.01) | -1.15 (-1.51 to -0.80) | -1.06 (-1.40 to -0.73) |
| Timor-Leste | -0.01 (-0.01 to 0) | 0.15 (0.12 to 0.19) | -0.42 (-0.59 to -0.24) | -0.45 (-0.62 to -0.27) |
| Togo | 0.01 (0.01 to 0.01) | -0.53 (-0.58 to -0.48) | -3.07 (-3.27 to -2.86) | -3.03 (-3.23 to -2.83) |
| Tokelau | 0.01 (0 to 0.03) | -0.07 (-0.09 to -0.05) | -0.16 (-1.80 to 1.50) | -0.20 (-1.82 to 1.45) |
| Tonga | -0.01 (-0.01 to -0.01) | -0.10 (-0.11 to -0.08) | -1.13 (-1.25 to -1.01) | -1.15 (-1.26 to -1.03) |
| Trinidad and Tobago | 0 | 0.39 (0.27 to 0.51) | -1.45 (-1.63 to -1.28) | -1.40 (-1.58 to -1.22) |
| Tunisia | 0 | 0.09 (0.01 to 0.16) | -4.29 (-4.36 to -4.22) | -4.22 (-4.29 to -4.16) |
| Turkey | -0.02 (-0.04 to -0.01) | 0.03 (-0.03 to 0.08) | -3.50 (-3.62 to -3.39) | -3.43 (-3.54 to -3.31) |
| Turkmenistan | 0.01 (0.01 to 0.01) | 0.53 (-0.05 to 1.11) | 0.12 (-1.24 to 1.51) | 0.11 (-1.24 to 1.47) |
| Tuvalu | -0.03 (-0.04 to -0.03) | -0.14 (-0.15 to -0.12) | -2.18 (-2.37 to -2.00) | -2.23 (-2.41 to -2.04) |
| Uganda | 0.01 (0.01 to 0.02) | -1.70 (-1.80 to -1.61) | -4.20 (-4.40 to -4.01) | -4.20 (-4.39 to -4.00) |
| Ukraine | 0 (0 to 0.01) | 0.45 (0.24 to 0.65) | 0.46 (-0.09 to 1.01) | 0.48 (-0.04 to 0.99) |
| United Arab Emirates | -0.02 (-0.02 to -0.01) | -0.31 (-0.39 to -0.22) | -3.17 (-3.57 to -2.78) | -3.06 (-3.44 to -2.68) |
| United Kingdom | 0.03 (-0.04 to 0.11) | 0.48 (-0.02 to 0.99) | -4.28 (-4.77 to -3.78) | -3.76 (-4.22 to -3.30) |
| United Republic of Tanzania | 0 | -2.09 (-2.22 to -1.96) | -3.59 (-3.73 to -3.45) | -3.59 (-3.73 to -3.46) |
| United States of America | -0.78 (-1.06 to -0.50) | 0.70 (0.26 to 1.14) | -1.93 (-2.37 to -1.50) | -1.64 (-2.06 to -1.22) |
| United States Virgin Islands | 0.01 (0.01 to 0.01) | 0.43 (0.17 to 0.69) | -2.32 (-2.64 to -1.99) | -2.06 (-2.36 to -1.76) |
| Uruguay | 0 (0 to 0.01) | -1.27 (-1.60 to -0.94) | -6.12 (-6.64 to -5.60) | -5.94 (-6.44 to -5.44) |
| Uzbekistan | 0.02 (0.02 to 0.02) | -1.44 (-1.83 to -1.06) | -1.71 (-2.60 to -0.81) | -1.70 (-2.57 to -0.82) |
| Vanuatu | -0.02 (-0.02 to -0.02) | -0.06 (-0.07 to -0.05) | -0.87 (-0.99 to -0.75) | -0.90 (-1.02 to -0.79) |
| Venezuela (Bolivarian Republic of) | 0 (0 to 0.01) | -0.17 (-0.27 to -0.08) | -1.42 (-1.88 to -0.96) | -1.39 (-1.83 to -0.94) |
| Viet Nam | 0.01 (0.01 to 0.01) | 0.50 (0.46 to 0.54) | -1.10 (-1.23 to -0.96) | -1.04 (-1.17 to -0.91) |
| Yemen | -0.04 (-0.04 to -0.03) | -0.16 (-0.20 to -0.13) | -1.87 (-1.97 to -1.77) | -1.87 (-1.98 to -1.77) |
| Zambia | 0 | -2.67 (-2.78 to -2.56) | -4.77 (-5.03 to -4.51) | -4.8 (-5.05 to -4.54) |
| Zimbabwe | 0 | 0.35 (0.20 to 0.51) | 2.28 (1.83 to 2.74) | 2.17 (1.74 to 2.60) |

ASR: age-standardized rate**,** DALY: disability-adjusted life years, EAPC: estimated average percent change, SDI: sociodemographic index, CI: confidence interval.

**Table S6: Main risk factors for estimated average percent change of ASR of DALYs for childhood myocarditis from 1990 to 2021**

| **Risk factors by SDI** | **Age standardized DALYs (per 100,000) (95%UI)** | | **EAPC, % (95% CI)** |
| --- | --- | --- | --- |
|  | **1990** | **2021** |  |
| **Low temperature** |  |  |  |
| Global | 1.91 (1.23 to 2.87) | 0.55 (0.35 to 0.83) | -3.98 (-4.17 to -3.79) |
| High | 1.36 (1.07 to 1.69) | 0.58 (0.45 to 0.71) | -2.91 (-3.42 to -2.39) |
| High-middle | 2.74 (1.91 to 3.9) | 0.62 (0.37 to 0.83) | -5.1 (-5.38 to -4.82) |
| Middle | 3.09 (1.94 to 4.58) | 0.67 (0.43 to 0.94) | -4.96 (-5.16 to -4.75) |
| Low-middle | 0.89 (0.27 to 2.13) | 0.6 (0.19 to 1.16) | -0.89 (-1.21 to -0.57) |
| Low | 0.73 (0.2 to 1.66) | 0.31 (0.13 to 0.58) | -2.79 (-3.11 to -2.47) |
| **High temperature** |  |  |  |
| Global | 0.56 (-0.08 to 1.41) | 0.32 (-0.05 to 0.74) | -1.06 (-1.46 to -0.67) |
| High | 0.26 (-0.05 to 0.64) | 0.16 (-0.01 to 0.34) | -1.09 (-1.7 to -0.47) |
| High-middle | 0.51 (-0.06 to 1.2) | 0.21 (-0.03 to 0.49) | -3.03 (-3.56 to -2.49) |
| Middle | 0.93 (0.03 to 1.99) | 0.37 (0.02 to 0.74) | -2.55 (-2.92 to -2.19) |
| Low-middle | 0.49 (-0.13 to 1.57) | 0.5 (-0.07 to 1.27) | 1.23 (0.64 to 1.83) |
| Low | 0.14 (-0.31 to 0.84) | 0.15 (-0.18 to 0.6) | 0.93 (0.01 to 1.86) |
| **Non-optimal temperature** |  |  |  |
| Global | 2.42 (1.29 to 4.09) | 0.84 (0.3 to 1.48) | -3.2 (-3.37 to -3.03) |
| High | 1.6 (1.12 to 2.24) | 0.73 (0.52 to 0.97) | -2.59 (-3.09 to -2.1) |
| High-middle | 3.21 (2.12 to 4.87) | 0.82 (0.49 to 1.25) | -4.72 (-5.01 to -4.44) |
| Middle | 3.93 (2.23 to 6.25) | 1 (0.53 to 1.57) | -4.35 (-4.53 to -4.17) |
| Low-middle | 1.32 (0.17 to 3.56) | 1.04 (0.12 to 2.24) | -0.08 (-0.39 to 0.23) |
| Low | 0.86 (0.06 to 2.41) | 0.45 (0 to 1.14) | -1.84 (-2.18 to -1.51) |

ASR: age-standardized rate**,** DALY: disability-adjusted life years, EAPC: estimated average percent change, SDI: sociodemographic index, CI: confidence interval. UI: uncertain interval.

**Table S7: Main risk factors for estimated average percent change of ASR of mortality for childhood myocarditis from 1990 to 2021**

| **Risk factors by SDI** | **Age standardized mortality (per 100,000) (95%UI)** | | **EAPC, % (95% CI)** |
| --- | --- | --- | --- |
|  | **1990** | **2021** |  |
| **Low temperature** |  |  |  |
| Global | 2.18 (1.41 to 3.28) | 0.63 (0.4 to 0.95) | -3.96 (-4.15 to -3.77) |
| High | 1.57 (1.23 to 1.95) | 0.68 (0.52 to 0.82) | -2.89 (-3.4 to -2.37) |
| High-middle | 3.13 (2.18 to 4.45) | 0.72 (0.43 to 0.97) | -5.05 (-5.33 to -4.77) |
| Middle | 3.52 (2.21 to 5.22) | 0.77 (0.49 to 1.09) | -4.92 (-5.12 to -4.71) |
| Low-middle | 1.01 (0.31 to 2.41) | 0.68 (0.22 to 1.32) | -0.88 (-1.2 to -0.56) |
| Low | 0.83 (0.23 to 1.89) | 0.35 (0.15 to 0.66) | -2.78 (-3.09 to -2.46) |
| **High temperature** |  |  |  |
| Global | 0.64 (-0.09 to 1.61) | 0.36 (-0.06 to 0.85) | -1.05 (-1.44 to -0.66) |
| High | 0.3 (-0.06 to 0.73) | 0.18 (-0.01 to 0.39) | -1.05 (-1.67 to -0.44) |
| High-middle | 0.58 (-0.07 to 1.38) | 0.25 (-0.04 to 0.57) | -2.97 (-3.5 to -2.44) |
| Middle | 1.06 (0.03 to 2.26) | 0.42 (0.03 to 0.85) | -2.53 (-2.89 to -2.16) |
| Low-middle | 0.55 (-0.15 to 1.78) | 0.57 (-0.08 to 1.44) | 1.26 (0.66 to 1.85) |
| Low | 0.16 (-0.35 to 0.95) | 0.17 (-0.2 to 0.68) | 0.94 (0.02 to 1.87) |
| **Non-optimal temperature** |  |  |  |
| Global | 2.76 (1.47 to 4.66) | 0.96 (0.35 to 1.7) | -3.18 (-3.35 to -3.01) |
| High | 1.85 (1.29 to 2.58) | 0.84 (0.6 to 1.13) | -2.57 (-3.07 to -2.07) |
| High-middle | 3.66 (2.42 to 5.57) | 0.95 (0.57 to 1.45) | -4.67 (-4.96 to -4.38) |
| Middle | 4.48 (2.54 to 7.12) | 1.15 (0.62 to 1.81) | -4.32 (-4.5 to -4.14) |
| Low-middle | 1.5 (0.19 to 4.03) | 1.18 (0.14 to 2.56) | -0.06 (-0.37 to 0.24) |
| Low | 0.97 (0.07 to 2.73) | 0.51 (0 to 1.3) | -1.83 (-2.17 to -1.5) |

ASR: age-standardized rate**,** DALY: disability-adjusted life years, EAPC: estimated average percent change, SDI: sociodemographic index, CI: confidence interval. UI: uncertain interval.

Hospital admission data/vital registration data worldwide (ICD code)

Disease Modelling Meta-Regression (DisMod-MR) or Cause of Death Ensemble model (CoDEm)

Global burden index including incidence, prevalence, mortality and DALY

Estimated annual percentage changes (EAPC)

Cross-country/region inequality analysis

Age-period-cohort effect analysis

Risk factors analysis

**Figure S1: Workflow diagram for global burden of childhood myocarditis**

A


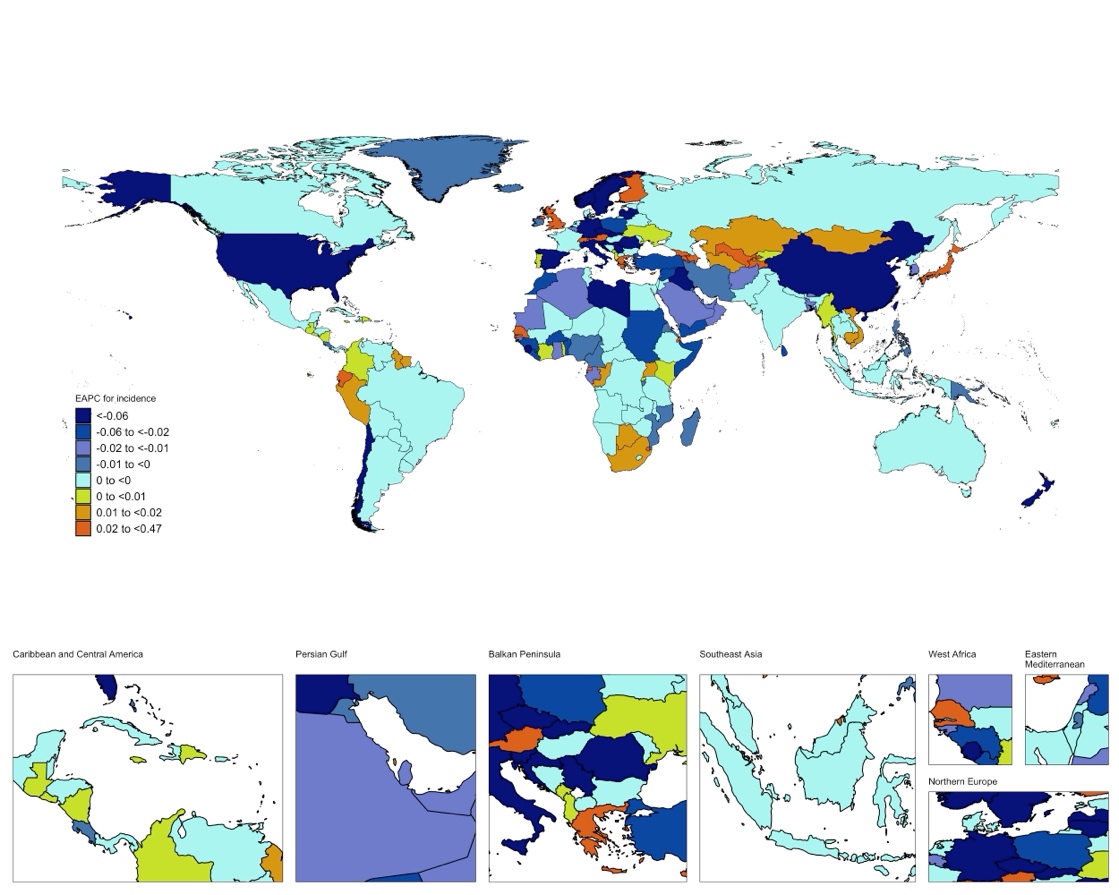


B

**
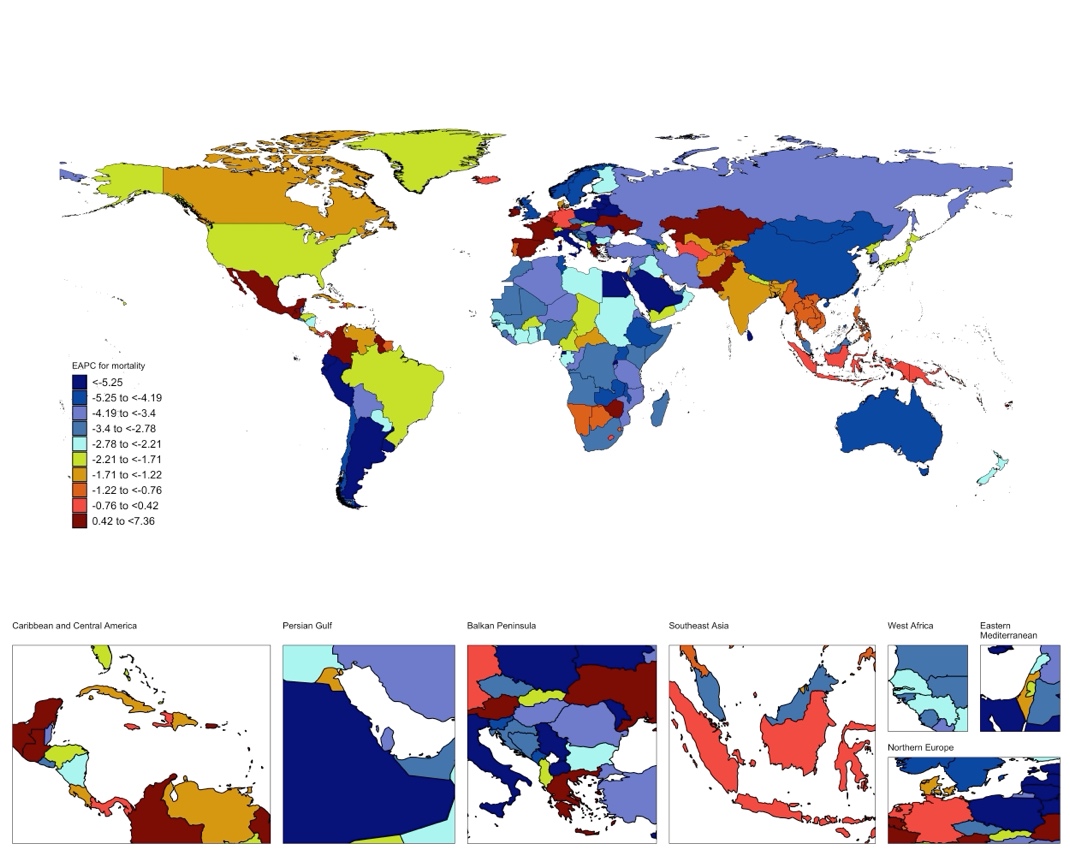
**

**Figure S2:** **Estimated average percent change of ASR of incidence (A) and mortality (B) for childhood myocarditis from 1990 to 2021 by country or territories** (ASR: age-standardized rate)

**
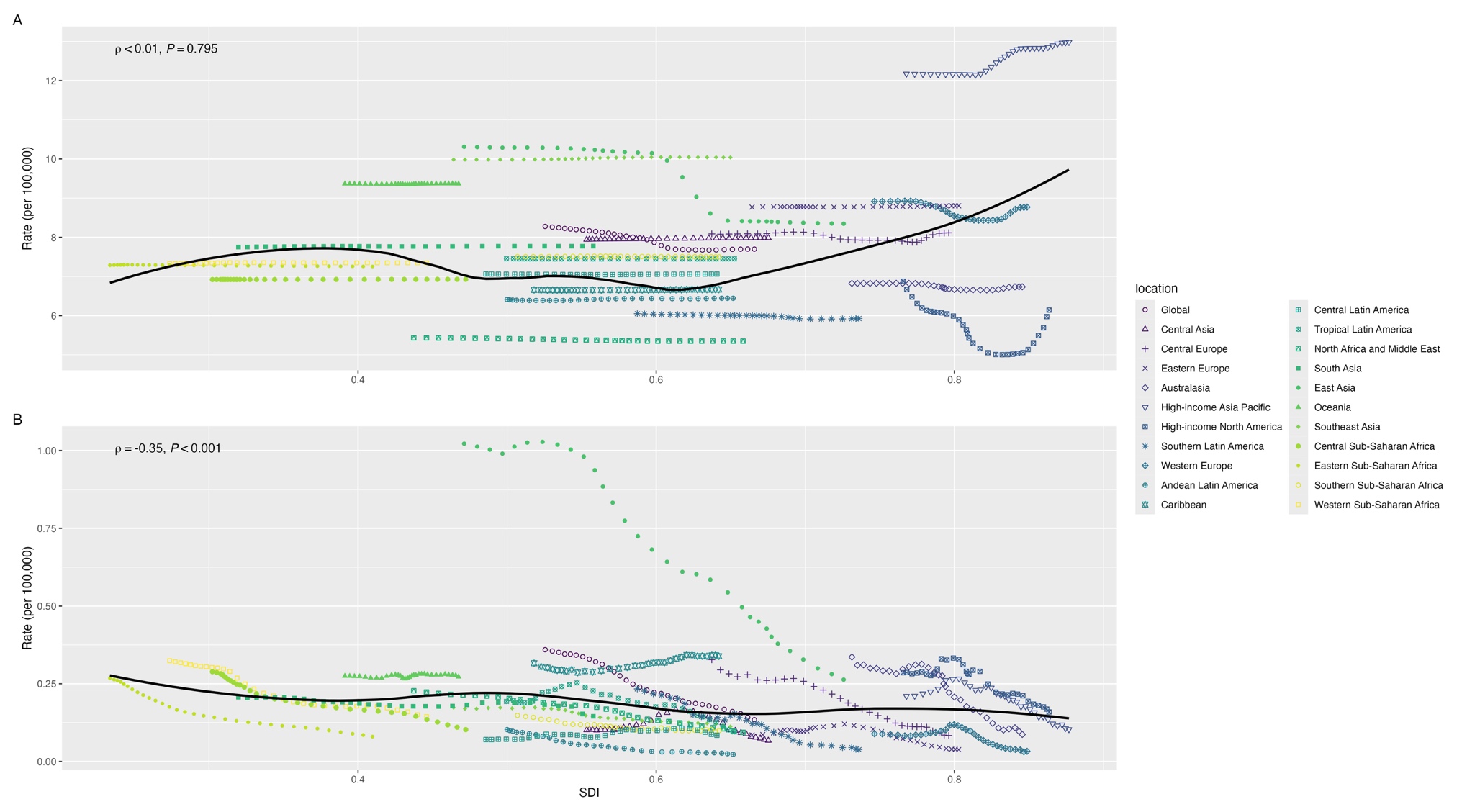
**

**Figure S3: The associations of SDI with ASR of incidence (A) and mortality (B) for childhood myocarditis from 1990 to 2021 by regions (**ASR: age-standardized rate**,** DALYs: disability-adjusted life years, SDI: sociodemographic index)

**
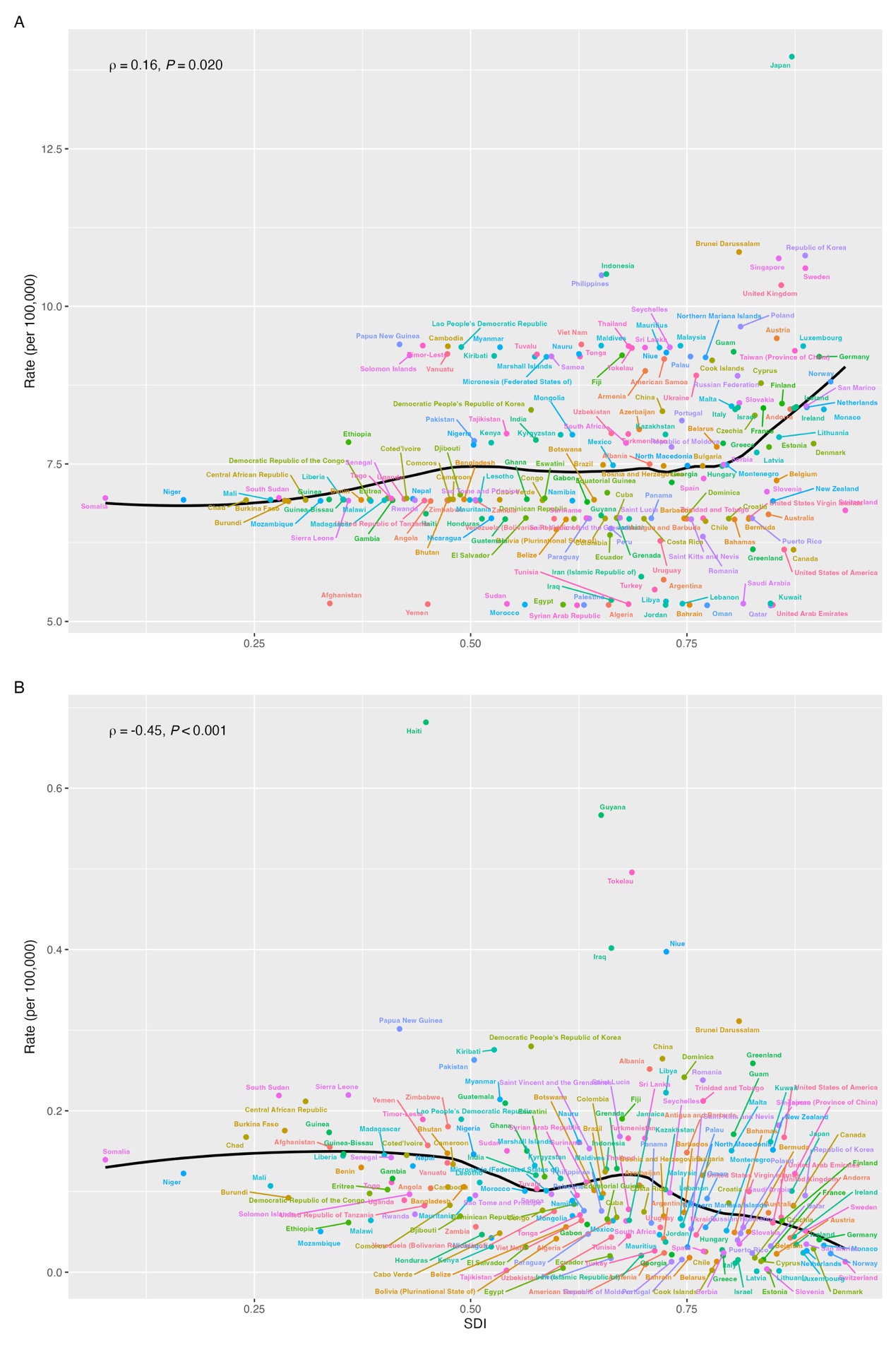
**

**Figure S4: The associations of SDI with ASR of incidence (A) and mortality (B) in 2021 for childhood myocarditis by country or territories (**ASR: age-standardized rate**,** DALYs: disability-adjusted life years, SDI: sociodemographic index)


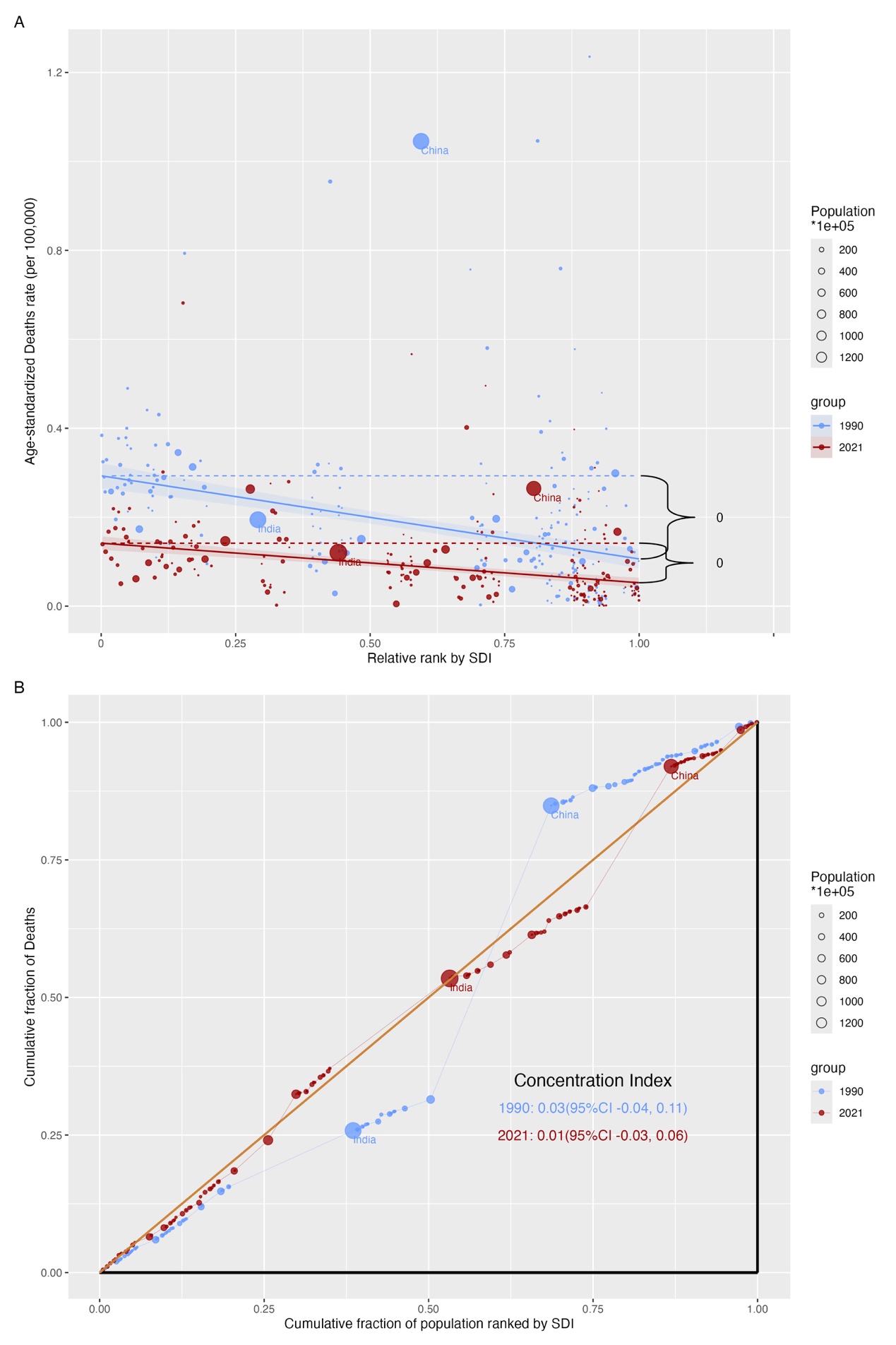


**Figure S5: SDI-related health inequality regression (A) and concentration (B) curves for ASR of mortality of childhood myocarditis from 1990 to 2021** (ASR: age-standardized rate**,** SDI: sociodemographic index).

**
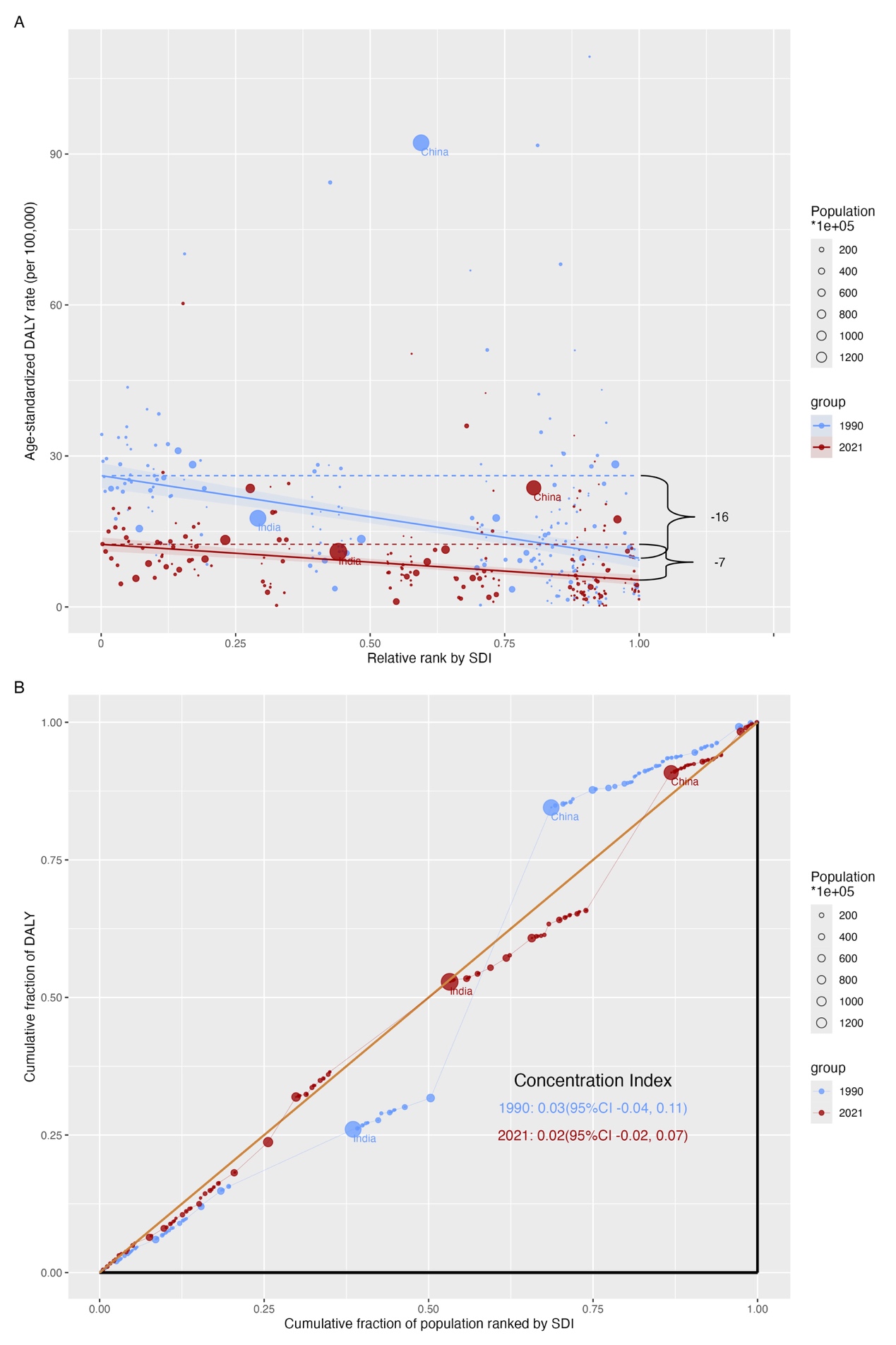
**

**Figure S6: SDI-related health inequality regression (A) and concentration (B) curves for ASR of DALYs of childhood myocarditis from 1990 to 2021** (ASR: age-standardized rate**,** SDI: sociodemographic index; DALYs, disability-adjusted life-years).


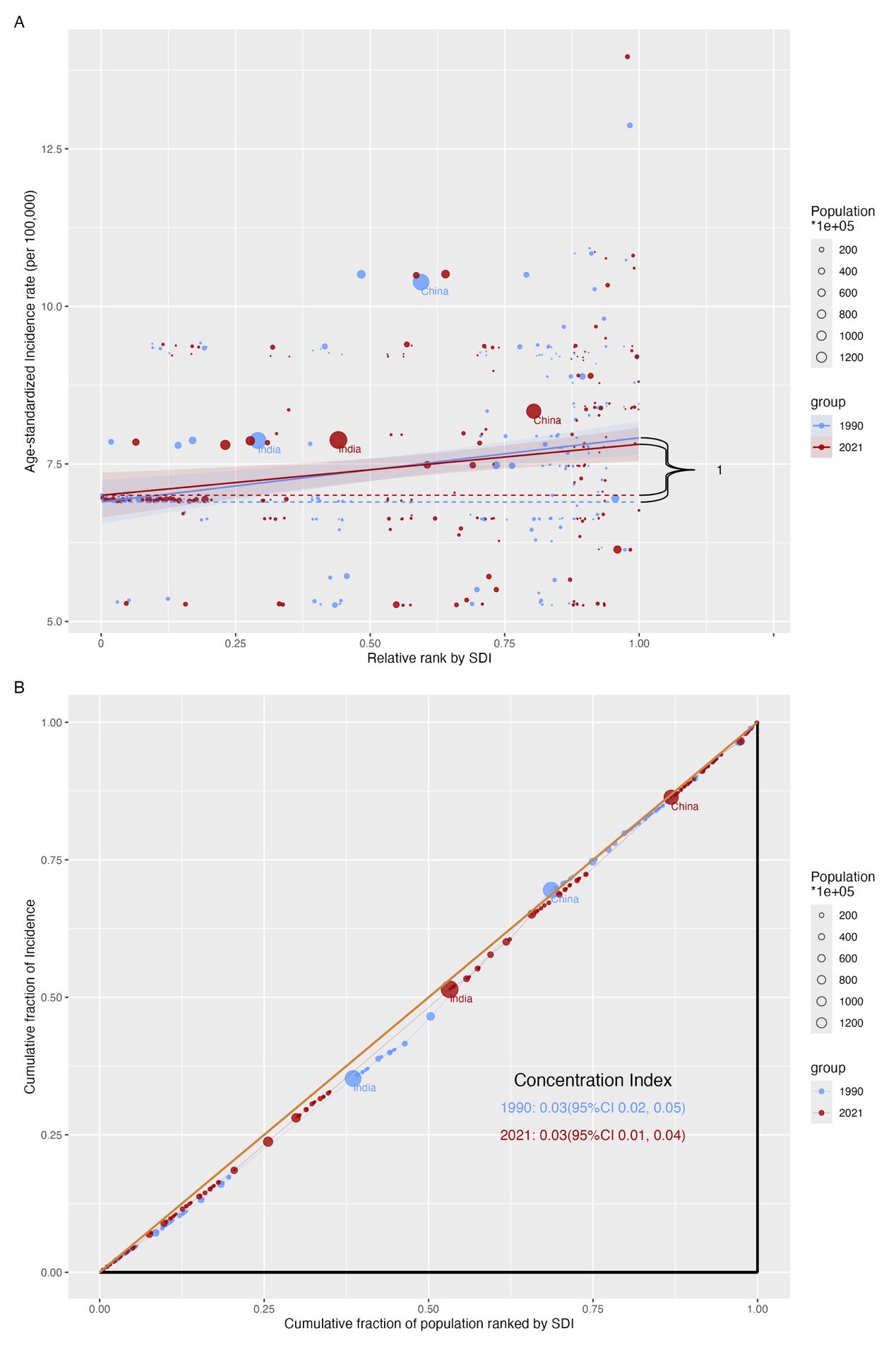


**Figure S7: SDI-related health inequality regression (A) and concentration (B) curves for ASR of incidence of childhood myocarditis from 1990 to 2021** (ASR: age-standardized rate**,** SDI: sociodemographic index).


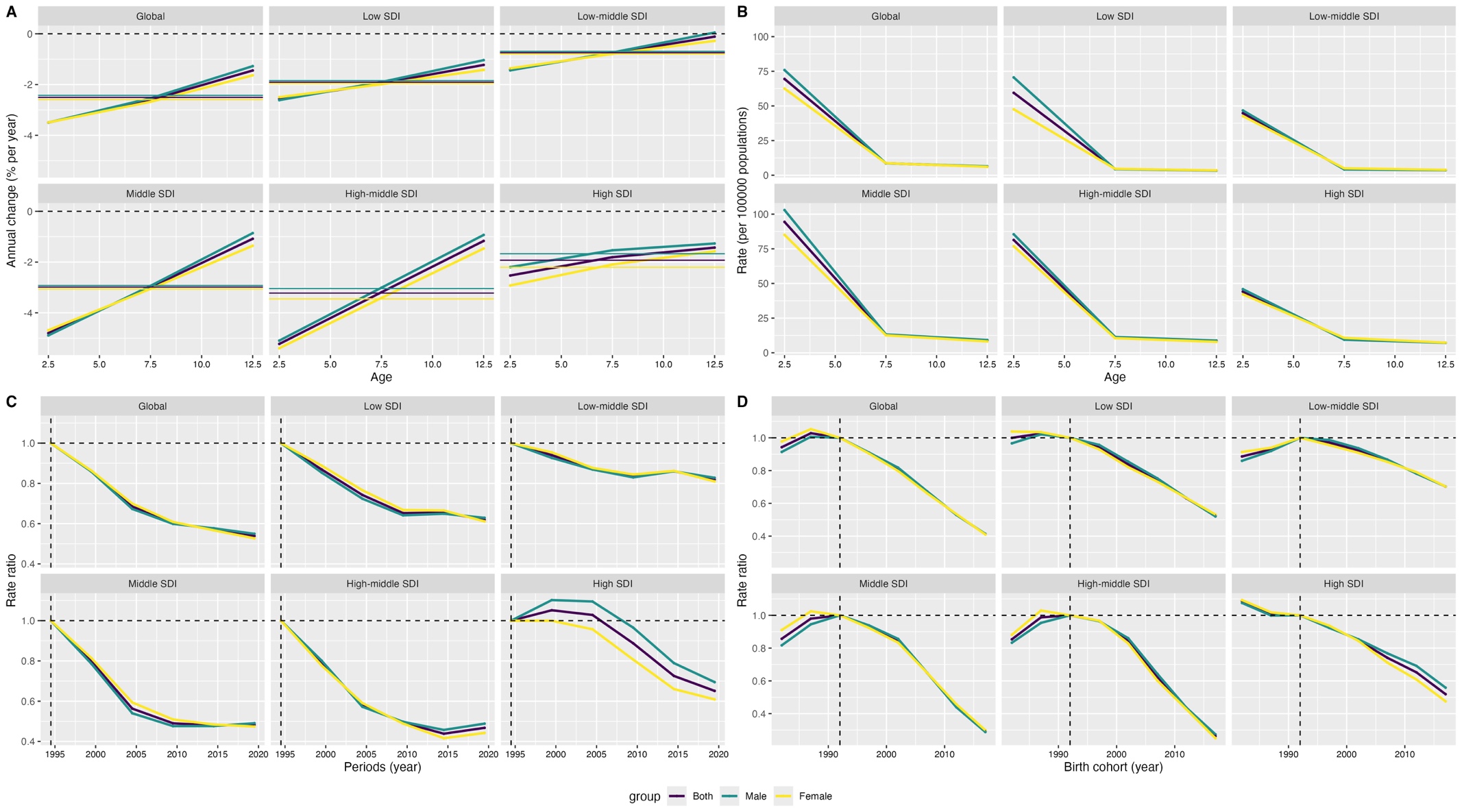


**Figure S8: Local drifts (A), age (B), period (C) and cohort (D) effects on DALYs rate of childhood myocarditis from 1990 to 2021** (SDI: sociodemographic index; DALYs, disability-adjusted life-years)

**
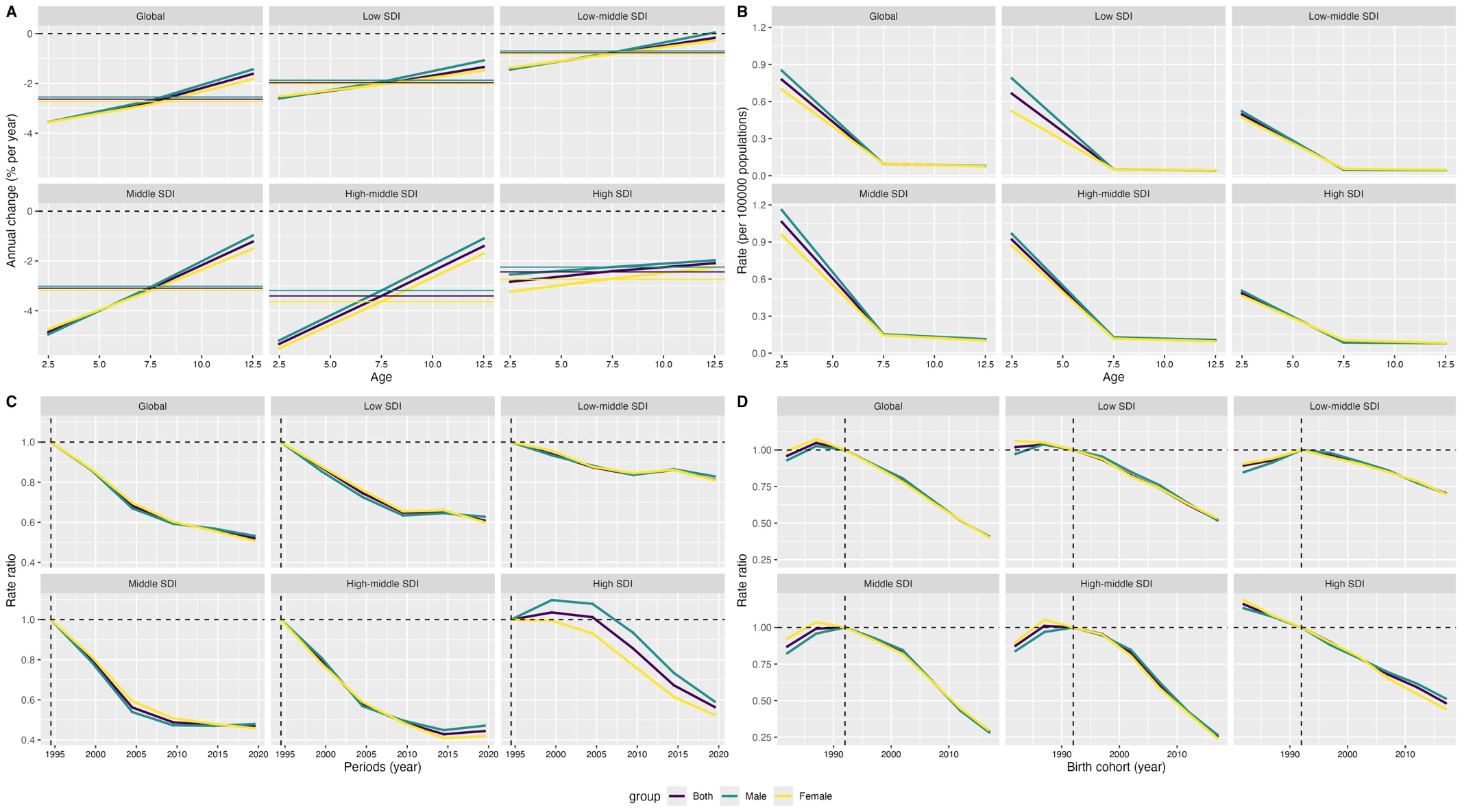
**

**Figure S9: Local drifts (A), age (B), period (C) and cohort (D) effects on mortality rate of childhood myocarditis from 1990 to 2021** (SDI: sociodemographic index)

**
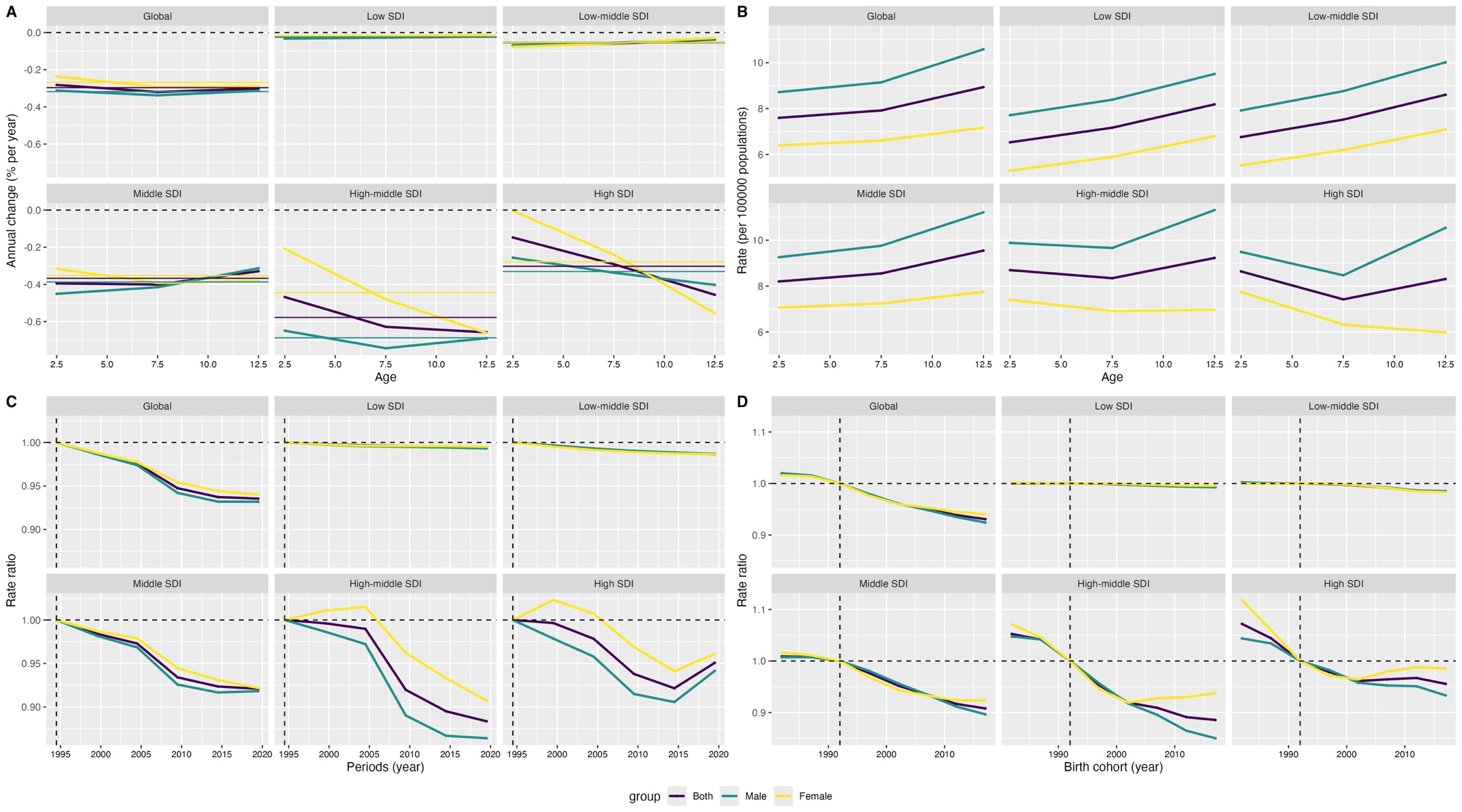
**

**Figure S10: Local drifts (A), age (B), period (C) and cohort (D) effects on incidence rate of childhood myocarditis from 1990 to 2021** (SDI: sociodemographic index)
